# Photoredox-catalyzed silyldifluoromethylation of silyl enol ethers

**DOI:** 10.3762/bjoc.16.126

**Published:** 2020-06-29

**Authors:** Vyacheslav I Supranovich, Vitalij V Levin, Alexander D Dilman

**Affiliations:** 1 N. D. Zelinsky Institute of Organic Chemistry, Leninsky prosp. 47, 119991 Moscow, Russian Federation

**Keywords:** difluoroalkylation, organofluorine compounds, photocatalysis, radical addition, silicon reagents

## Abstract

A method for the light-mediated fluoroalkylation of silyl enol ethers with (bromodifluoromethyl)trimethylsilane followed by a reduction of the primary products with sodium borohydride is described. An 18 W, 375 nm LED was used as the light source. The reaction is performed in the presence of a gold photocatalyst, which effects the generation of a (trimethylsilyl)difluoromethyl radical via cleavage of the carbon–bromine bond.

## Findings

Fluorinated silicon reagents have found widespread use for the introduction of fluorinated fragments [1–5]. Typically, these reagents work under strongly basic conditions required to activate inert C–Si bonds with the generation of carbanionic species. On the other hand, radical reactions offer different synthetic opportunities originating from the neutral character of the intermediates [6–7] and, accordingly, radical fluoroalkylation processes have been extensively investigated over the last decade [8–13].

**Scheme 1:**
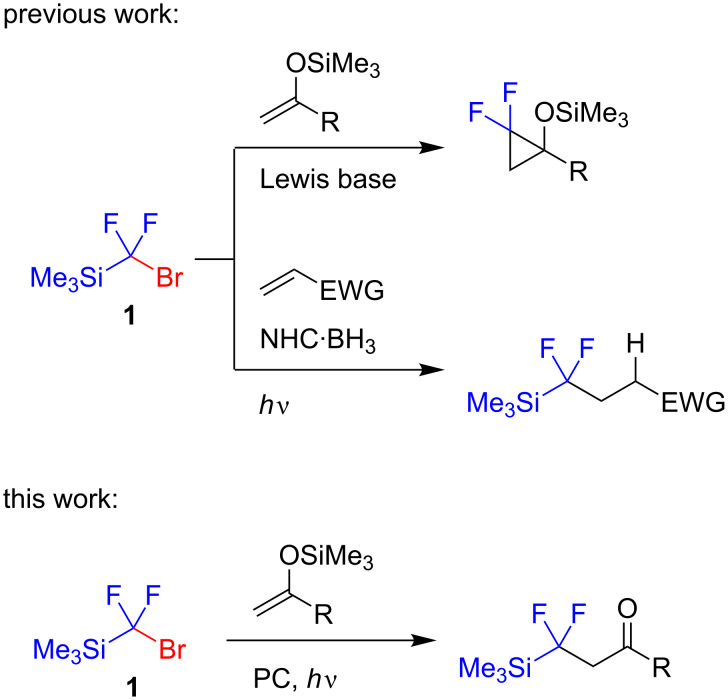
Reactions of (bromodifluoromethyl)trimethylsilane (**1**).

Recently, (bromodifluoromethyl)trimethylsilane (**1**) which can be readily obtained from the Ruppert–Prakash reagent [14–15], has been introduced as a reagent for the synthesis of various difluorinated compounds [16–18]. This silane is very sensitive to Lewis bases and accordingly it was used as a precursor of difluorocarbene, which can react with enol ethers [19–20] (Scheme 1). We showed that this silane could be involved in the radical chain hydrofluoroalkylation of electron-deficient alkenes, using a boron hydride as a source of hydrogen [21]. We thought that silane **1** could couple with silyl enol ethers in the presence of a photocatalyst affording fluoroalkylation products. Indeed, silyl enol ethers were found to be good acceptors of fluorinated radicals, and the resultant silyloxy-substituted radicals underwent single-electron oxidation thereby supporting a photoredox cycle [22–24].

**Scheme 2:**
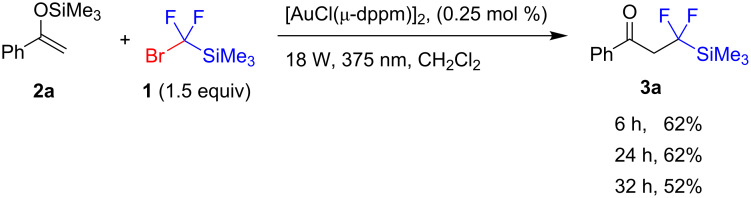
Optimization studies. Yield determined by ^19^F NMR spectroscopy using an internal standard.

The silyl enol ether **2a** derived from acetophenone was selected as a model substrate and the reaction with silane **1** (1.5 equiv) was evaluated (Scheme 2). The reactions were performed in dichloromethane, and reaction mixtures were analyzed by ^19^F NMR spectroscopy. A series of typical photocatalysts (for example, iridium catalysts) were ineffective in promoting the reaction. Rewardingly, a gold catalyst, [AuCl(μ-dppm)]_2_ [25–27], provided reasonable yields of **3a** after one day of irradiation along with a full conversion of the starting enol ether. Moreover, a further increase in reaction time was accompanied by a decrease in the product yield. Finally, GC monitoring suggested complete consumption of the silyl enol ether within 6 hours. It should also be pointed out that the addition of basic additives, which are frequently employed in photoredox reactions to scavenge acidic byproducts [28–29], could not be employed. Silane **1** is easily destroyed by bases (even by the amide group [30]) followed by the rapid addition of difluorocarbene to silyl enol ethers [19–20]. Disappointingly, we were unable to isolate ketone **3a** using flash column chromatography on silica gel, presumably, owing to facile β-elimination of hydrogen fluoride. To isolate a stable product, the reaction mixture was treated with sodium borohydride in ethanol, which effected the reduction of the keto group affording the corresponding alcohol **4a** in 52% yield.

**Figure 1:**
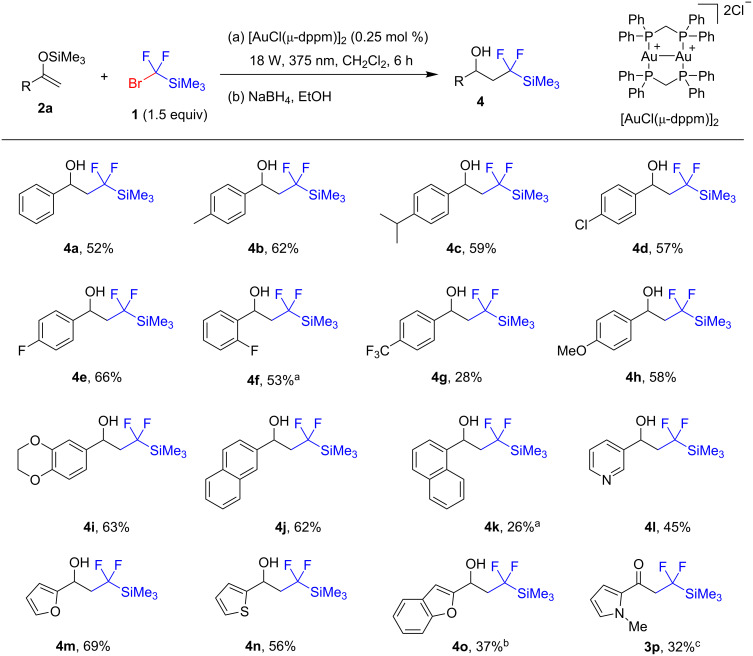
Reaction of silyl enol ethers. Yields refer to isolated yields. ^a^Reaction time 24 h; ^b^1.0 equiv of silane **1** was used; ^c^ketone was isolated.

Under the optimized conditions, a series of silyl enol ethers **2** were reacted with silane **1** (Figure 1). The reaction worked well with enol ethers derived from aromatic ketones, while those obtained from aliphatic ketones were ineffective. This may be ascribed to the decreased radical-stabilizing effect of the alkyl group compared to that of an aryl group, which either makes radical addition reversible or attenuates the reactivity of the starting enol ether. Similarly, an aromatic substrate bearing an *ortho* substituent gave lower yields, which may be associated with the ability of the *ortho* group to disfavor the planar conformation needed to stabilize an intermediate benzyl radical. The complete conversions of the enol ethers were usually achieved within 6 hours, though to obtain products **4f**,**k** a longer time of 24 h was needed. Generally, higher yields were observed with substrates containing electron-donating functional groups. Substrates containing pyridine, furan, and thiophene as heterocyclic fragments were also successfully converted into the corresponding alcohols **4**. However, in the reaction of the enol ether derived from 2-acetyl-*N*-methylpyrrole, ketone **3p** did not undergo reduction with sodium borohydride, and the decreased reactivity of this ketone allowed its isolation.

**Scheme 3:**
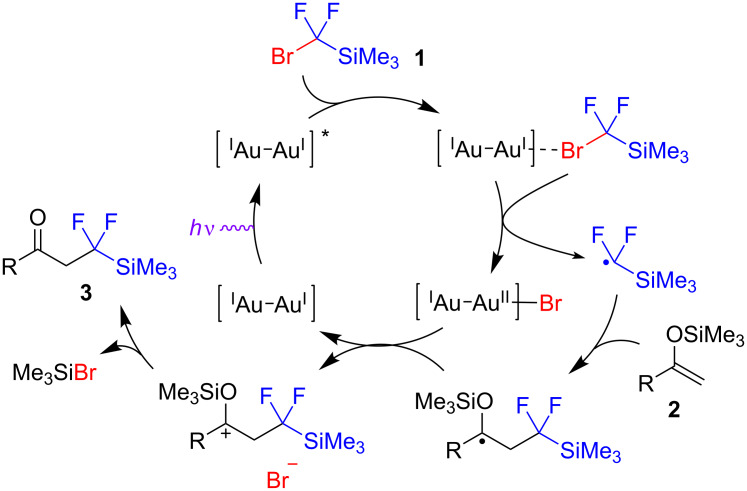
Proposed mechanism of the fluoroalkylation reaction.

A proposed mechanism for the photoredox fluoroalkylation reaction is shown in Scheme 3. The photoexcited catalyst converts silane **1** into difluoromethylsilyl-based radical. The efficiency of [AuCl(μ-dppm)]_2_ compared to other strongly reducing catalysts may be associated with the ability of gold to interact with the bromine atom of silane **1** followed by inner-sphere electron transfer [27]. The radical then attacks silyl enol ether **2**, and the subsequent silyloxy-substituted radical is oxidized by the photocatalyst to generate the product with concomitant production of TMSBr.

## Conclusion

In summary, a method for the introduction of the silyldifluoromethyl group was described by the coupling of a readily available silicon reagent with silyl enol ethers. The reaction is promoted by light and involves the generation of a fluorinated carbon-based radical via cleavage of the carbon–bromine bond by a gold photocatalyst.

## Supporting Information

File 1 Full experimental details, compound characterization, and copies of NMR spectra.
