# Supplementary material for: Photoredox-catalyzed silyldifluoromethylation of silyl enol ethers
Source: Beilstein J Org Chem. 2020 Jun 29;16:1550–3. doi: 10.3762/bjoc.16.126 (PMC7356207; doi:10.3762/bjoc.16.126)

BEILSTEIN JOURNAL OF ORGANIC CHEMISTRY

Supporting Information

for

Photoredox-catalyzed silyldifluoromethylation of silyl enol ethers

Vyacheslav I. Supranovich, Vitalij V. Levin and Alexander D. Dilman

Beilstein J. Org. Chem. **2020,** *16,* 1550–1553. doi:10.3762/bjoc.16.126

Full experimental details, compound characterization, and copies of NMR spectra

License and Terms: This is a supporting information file under the terms of the Creative Commons Attribution License (http://creativecommons.org/licenses/by/4.0). Please note that the reuse, redistribution and reproduction in particular requires that the authors and source are credited.
The license is subject to the *Beilstein Journal of Organic Chemistry* terms and conditions: (https://www.beilstein-journals.org/bjoc)

Table of contents

	Page
General Methods	S2
Procedures	S2
Compound Characterization Data	S3-S9
NMR Spectra	S10–S57

General Methods. Dichloromethane was distilled from CaH_2. Column chromatography was carried out employing silica gel (230–400 mesh). Precoated silica gel plates F-254 were used for thin-layer analytical chromatography visualizing with UV and/or acidic aq. $KMnO_4$ solution. High resolution mass spectra (HRMS) were measured using electrospray ionization (ESI) and a time-of-flight (TOF) mass analyzer. The measurements were done in a positive ion mode (interface capillary voltage – 4500 V) or in a negative ion mode (3200 V); mass range from *m/z* 50 to *m/z* 3000.

Reagents. Silyl enol ethers **2** were prepared according to a literature procedure and distilled under vacuum.[1] (Bromodifluoromethyl)trimethylsilane (**1**)[2] and photocatalyst $[AuCl(\mu\text{-dppm})]_2$[3] were prepared according to literature procedures.

Reaction of silyl enol ethers with silane 1 (general procedure).

(a) A tube (Duran, Roth cat. no K248.1, outside diameter = 12 mm) containing a stirring bar was charged with $[AuCl(\mu\text{-dppm})]_2$ (1.5 mg, 0.0012 mmol, 0.25 mol %), and then was evacuated and filled with argon. Dichloromethane (2 mL), silyl enol ether **2** (0.50 mmol), and silane **1** (for **4a–n** and **3p**, 152 mg, 0.75 mmol, 1.5 equiv; for **4o**, 102 mg, 0.50 mmol, 1.0 equiv) were added. The tube was closed tightly with a screw-cap and placed in a water bath. The reaction mixture was stirred, and the tube was irradiated by a 375 nm LED chip operated at 18 watt, distance between LED chip and the reaction tube was 1 cm. Irradiation time: for **4a–e**,**g–j**,**l–o** and **3p**, 6 hours; for **4f**,**k**, 24 h. During the reaction, the bath temperature was maintained in a range of 13–15 °C.

(b) For **4a–p**, the mixture was poured into a 100 mL flask containing $NaBH_4$ (76 mg, 2.0 mmol, 4.0 equiv) and ethanol (3 mL) cooled with ice/water. The reaction tube was rinsed with additional ethanol (2 mL) which was also poured into the flask, and the mixture was stirred for one hour at 0 to 5 °C. For the work-up, hydrochloric acid (1 M, 1 mL) was added followed by water (10 mL). For **4l** the solution was neutralized with solid NaOH (230 mg). The organic phase was separated, and the aqueous phase was extracted with dichloromethane (3 × 2 mL). The combined organic phases were dried with Na_2SO_4, concentrated under vacuum, and the residue was purified by column chromatography on silica gel.

(c) For **3p**, water (10 mL) was added, the organic phase was separated, and the aqueous phase was extracted with dichloromethane (3 × 2 mL). The combined organic phases were dried with Na_2SO_4, concentrated under vacuum, and the residue was purified by column chromatography on silica gel.

[1] Scherbinina, S. I.; Fedorov, O. V.; Levin, V. V.; Kokorekin, V. A.; Struchkova, M. I.; Dilman, A. D. *J. Org. Chem.* **2017,** *82*, 12967–12974.

[2] Kosobokov, M. D.; Dilman, A. D.; Levin, V. V.; Struchkova, M. I. *J. Org. Chem.* **2012,** *77*, 5850–5855.

[3] Massai, L.; Fernández-Gallardo, J.; Guerri, A.; Arcangeli, A.; Pillozzi, S.; Contel, M.; Messori, L. *Dalton Trans.* **2015,** *44*, 11067–11076.

3,3-Difluoro-1-phenyl-3-(trimethylsilyl)propan-1-ol (4a).

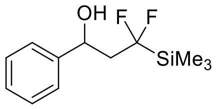

Yield 63 mg (52 %). Colorless oil.

Chromatography: hexanes/EtOAc, from 20/1 to 10/1. R_f 0.26 (hexanes/EtOAc, 20/1).

^{1}H NMR (300 MHz, $CDCl_3$) δ: 7.44 – 7.23 (m, 5H), 5.25 (d, *J* = 9.3 Hz, 1H), 2.50 (br s, 1H), 2.45 – 2.02 (m, 2H), 0.22 (s, 9H).

^{13}C {^{1}H} NMR (75 MHz, $CDCl_3$) δ: 144.1, 130.8 (t, *J* = 259.1 Hz), 128.7, 127.8, 125.8, 68.7 (dd, *J* = 7.9 , 5.9 Hz), 45.4 (dd, *J* = 19.4, 18.1 Hz), -4.5 (t, *J* = 2.4 Hz).

^{19}F NMR (282 MHz, $CDCl_3$) δ: -113.7 (ddd, *J* = 315.7, 31.3, 11.0 Hz, 1F), -115.0 (dddd, *J* = 315.7, 33.9, 12.8, 3.5 Hz, 1F).

HRMS (ESI): calcd for $C_{12}H_{22}F_2NO_2Si$ (M+NH_4) 262.1433, found 262.1434.

3,3-Difluoro-1-(*p*-tolyl)-3-(trimethylsilyl)propan-1-ol (4b).

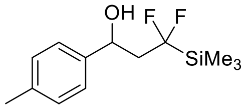

Yield 80 mg (62 %). Yellow oil.

Chromatography: hexanes/EtOAc, from 7/1 to 5/1. R_f 0.22 (hexanes/EtOAc, 7/1).

^{1}H NMR (300 MHz, $CDCl_3$) δ: 7.32 (d, *J* = 7.9 Hz, 2H), 7.21 (d, *J* = 7.9 Hz, 2H), 5.23 (d, *J* = 9.3 Hz, 1H), 2.48 (br s, 1H), 2.39 (s, 3H), 2.44-2.03 (m, 2H), 0.24 (s, 9H)

^{13}C {^{1}H} NMR (75 MHz, $CDCl_3$) δ: 141.2, 137.4, 130.8 (t, *J* = 258.8 Hz), 129.3, 125.7, 68.5 (dd, *J* = 8.0, 5.9 Hz), 45.3 (dd, *J* = 19.3, 18.3 Hz), 21.2, -4.5 (t, *J* = 2.3 Hz)

^{19}F NMR (282 MHz, $CDCl_3$) δ: -113.5 (ddd, *J* = 315.4, 29.3, 13.3 Hz, 1F), -115.2 (dddd, *J* = 315.4, 31.6, 13.7, 4.9 Hz, 1F)

HRMS (ESI): calcd for $C_{13}H_{20}F_2NaOSi$ (M+Na) 281.1144, found 281.1155.

3,3-Difluoro-1-(4-isopropylphenyl)-3-(trimethylsilyl)propan-1-ol (4c).

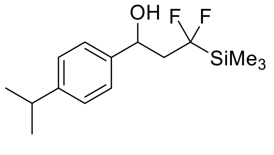

Yield 85 mg (59 %). Colorless oil.

Chromatography: hexanes/EtOAc, 10/1. R_f 0.28 (hexanes/EtOAc, 10/1).

^{1}H NMR (300 MHz, $CDCl_3$) δ: 7.35 (d, *J* = 8.0 Hz, 2H), 7.27 (d, *J* = 8.0 Hz, 2H), 5.24 (d, *J* = 9.2 Hz, 1H), 2.96 (sept, *J* = 7.0 Hz, 1H), 2.50 (br d, *J* = 5.3 Hz, 1H), 2.46 – 2.05 (m, 2H), 1.31 (d, *J* = 7.0 Hz, 6H), 0.25 (s, 9H).

^{13}C {^{1}H} NMR (75 MHz, $CDCl_3$) δ: 148.4, 141.5, 130.8 (dd, *J* = 259.6, 258.2 Hz), 126.7, 125.8, 68.5 (dd, *J* = 8.1, 5.9 Hz), 45.2 (dd, *J* = 19.3,18.2 Hz), 33.9, 24.1, -4.2 (t, *J* = 2.3 Hz).

^{19}F NMR (282 MHz, $CDCl_3$) δ: -113.5 (ddd, *J* = 315.4, 29.0, 13.4 Hz, 1F), -115.1 (dddd, *J* = 315.4, 31.6, 13.9, 5.3 Hz, 1F).

HRMS (ESI): calcd for $C_{15}H_{24}F_2NaOSi$ (M+Na) 309.1457, found 309.1459.

1-(4-Chlorophenyl)-3,3-difluoro-3-(trimethylsilyl)propan-1-ol (4d).

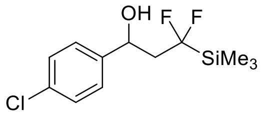

Yield 80 mg (57 %). Yellowish crystals. Mp 43 – 44 °C.

Chromatography: hexanes/EtOAc, 10/1. R_f 0.28 (hexanes/EtOAc, 10/1).

^{1}H NMR (300 MHz, $CDCl_3$) δ: 7.34 (m, 4H), 5.24 (d, *J* = 9.1 Hz, 1H), 2.59 (br d, *J* = 4.9 Hz, 1H), 2.42-1.96 (m, 2H), 0.22 (s, 9H).

^{13}C {^{1}H} NMR (75 MHz, $CDCl_3$) δ: 142.5, 133.4, 130.7 (t, *J* = 258 .3 Hz), 128.8, 127.2, 68.0 (dd, *J* = 7.9, 5.8 Hz), 45.2 (dd, *J* = 19.6, 18.1 Hz), -4.6 (t, *J* = 2.3 Hz).

^{19}F NMR (282 MHz, $CDCl_3$) δ: -114.1 (ddd, *J* = 315.4, 30.2, 12.4 Hz, 1F), -115.9 (dddd, *J* = 315.4, 31.8, 11.1, 4.9 Hz).

HRMS (ESI): calcd for $C_{12}H_{17}F_5{}^{35}ClNaOSi$ (M+Na) 301.0597, found 301.0599, $C_{12}H_{17}F_5{}^{37}ClNaOSi$ (M+Na) 303.0569, found 303.0569.

3,3-Difluoro-1-(4-fluorophenyl)-3-(trimethylsilyl)propan-1-ol (4e).

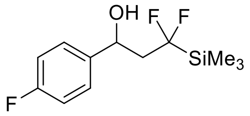

Yield 87 mg (66 %). Yellow oil.

Chromatography: hexanes/EtOAc, 5/1. R_f 0.37 (hexanes/EtOAc, 5/1).

^{1}H NMR (300 MHz, $CDCl_3$) δ: 7.35 (dd, *J* = 8.7, 5.4 Hz, 2H), 7.03 (dd, *J* = 8.7, 8.7 Hz, 2H), 5.22 (d, *J* = 8.9 Hz, 1H), 2.56 (br d, *J* = 4.4 Hz, 1H), 2.40-1.97 (m, 2H), 0.20 (s, 9H).

^{13}C {^{1}H} NMR (75 MHz, $CDCl_3$) δ: 162.3 (d, *J* = 245.5 Hz), 139.8 (d, *J* = 3.2 Hz), 130.7 (dd, *J* = 259.9, 258.0 Hz), 127.4 (d, *J* = 8.1 Hz), 115.5 (d, *J* = 21.4 Hz), 68.1 (dd, *J* = 7.9, 6.0 Hz), 45.3 (dd, *J* = 19.0, 17.7 Hz), -4.6 (d, *J* = 2.3 Hz).

^{19}F NMR (282 MHz, $CDCl_3$) δ: -113.8 (ddd, *J* = 315.4, 29.9, 12.9 Hz, 1F), -115.5 (dddd, *J* = 315.4, 31.9, 12.9, 4.4 Hz, 1F), -115.7 (tt, *J* = 8.7, 5.4 Hz, 1F).

HRMS (ESI): calcd for $C_{12}H_{17}F_3NaOSi$ (M+Na) 285.0893, found 285.0893.

3,3-Difluoro-1-(2-fluorophenyl)-3-(trimethylsilyl)propan-1-ol (4f).

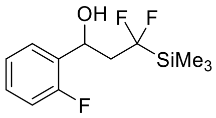

Yield 69 mg (53 %). Yellow oil.

Chromatography: hexanes/EtOAc, 5/1. R_f 0.22 (hexanes/EtOAc, 5/1).

^{1}H NMR (300 MHz, $CDCl_3$) δ: 7.58 (td, *J* = 7.6, 1.6 Hz, 1H), 7.33-7.25 (m, 1H), 7.19 (td, *J* = 7.6, 1.3 Hz, 1H), 7.05 (ddd, *J* = 10.6, 8.2, 1.2 Hz, 1H), 5.55 (d, *J* = 8.8 Hz, 1H), 2.60 (m, 1H), 2.43-2.11 (m, 2H), 0.24 (s, 9H).

^{13}C {^{1}H} NMR (75 MHz, $CDCl_3$) δ: 159.5 (d, *J* = 245.7 Hz), 130.9 (d, *J* = 13.0 Hz), 130.8 (dd, J = 259.7, 258.4 Hz), 129.1 (d, *J* = 8.2 Hz), 127.4 (d, *J* = 4.3 Hz), 124.5 (d, *J* = 3.5 Hz), 115.4 (d, *J* = 21.5 Hz), 63.1 (ddd, *J* = 9.9, 6.9, 3.0 Hz), 43.9 (td, *J* = 18.8, 1.3 Hz), -4.6 (t, *J* = 2.3 Hz).

^{19}F NMR (282 MHz, $CDCl_3$) δ: -113.7 (dddd, *J* = 314.7, 26.5, 16.7, 4.6 Hz, 1F), -114.5 (ddd, *J* = 314.7, 25.3, 18.5 Hz, 1F), -120.0 (m, 1F).

HRMS (ESI): calcd for $C_{12}H_{17}F_3NaOSi$ (M+Na) 285.0893, found 285.0891.

3,3-Difluoro-1-[4-(trifluoromethyl)phenyl]-3-(trimethylsilyl)propan-1-ol (4g).

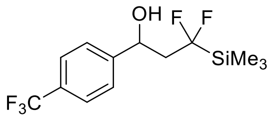

Yield 44 mg (28 %). Colorless crystals. Mp 55 – 57 °C.

Chromatography: hexanes/EtOAc, 10/1. R_f 0.32 (hexanes/EtOAc, 10/1).

^{1}H NMR (300 MHz, $CDCl_3$) δ: 7.62 (d, *J* = 8.1 Hz, 2H), 7.51 (d, *J* = 8.1 Hz, 2H), 5.33 (d, *J* = 9.1 Hz, 1H), 2.63 (d, *J* = 6.3 Hz, 1H), 2.40 – 1.97 (m, 2H), 0.20 (s, 9H).

^{13}C {^{1}H} NMR (75 MHz, $CDCl_3$) δ: 148.0, 130.7 (t, *J* = 257.6 Hz), 130.0 (q, *J* = 32.4 Hz), 126.2, 125.6 (q, *J* = 3.8 Hz), 124.3 (q, *J* = 272.1 Hz), 68.2 (dd, *J* = 7.6, 5.9 Hz), 45.2 (dd, *J* = 19.3, 17.5 Hz), -4.7 (t, *J* = 2.2 Hz).

^{19}F NMR (282 MHz, $CDCl_3$) δ: -63.3 (s, 3F), -114.1 (ddd, *J* = 315.4, 30.6, 12.4 Hz, 1F), -115.8 (dddd, *J* =315.4, 32.4, 11.6, 6.3 Hz, 1F).

HRMS (ESI): calcd for $C_{13}H_{17}F_5NaOSi$ (M+Na) 335.0861, found 335.0851.

3,3-Difluoro-1-(4-methoxyphenyl)-3-(trimethylsilyl)propan-1-ol (4h).

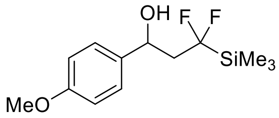

Yield 79 mg (58 %). Colorless oil.

Chromatography: hexanes/EtOAc, 4/1. R_f 0.35 (hexanes/EtOAc, 4/1).

^{1}H NMR (300 MHz, $CDCl_3$) δ: 7.33 (d, *J* = 8.7 Hz, 2H), 6.91 (d, *J* = 8.7 Hz, 2H), 5.20 (d, *J* = 9.1 Hz, 1H), 3.83 (s, 3H), 2.48 (br d, *J* = 4.4 Hz 1H), 2.43 – 2.01 (m, 2H), 0.23 (s, 9H).

^{13}C {^{1}H} NMR (75 MHz, $CDCl_3$) δ: 159.3, 136.4, 130.7 (t, *J* = 259.0 Hz), 127.0, 114.1, 68.3 (dd, *J* = 8.0, 5.9 Hz), 55.4, 45.3 (dd, *J* = 19.4, 18.2 Hz), -4.5 (t, *J* = 2.3 Hz).

^{19}F NMR (282 MHz, $CDCl_3$) δ: -113.5 (ddd, *J* = 315.7, 28.9, 13.6 Hz, 1F), -115.0 (dddd, *J* = 315.7, 31.6, 13.9, 4.4 Hz, 1F).

HRMS (ESI): calcd for $C_{13}H_{20}F_2NaO_2Si$ (M+Na) 297.1093, found 297.1091.

1-(2,3-Dihydrobenzo[*b*][1,4]dioxin-6-yl)-3,3-difluoro-3-(trimethylsilyl)propan-1-ol (4i).

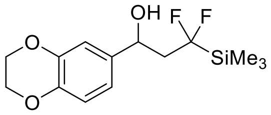

Yield 95 mg (63 %). Yellow oil. Chromatography: hexanes/EtOAc, 4/1. R_f 0.34 (hexanes/EtOAc, 4/1).

^{1}H NMR (300 MHz, $CDCl_3$) δ: 6.90 (s, 1H), 6.84-6.81 (m, 2H), 5.09 (d, *J* = 9.1 Hz,1H), 4.23 (s, 4H), 2.48 (br s, 1H), 2.41-1.95 (m, 2H), 0.20 (s, 9H).

^{13}C {^{1}H} NMR (75 MHz, $CDCl_3$) δ: 143.6, 143.1, 137.6, 130.6 (t, *J* = 258.8 Hz), 118.7, 117.3, 114.7, 68.2 (dd, *J* = 8.1, 5.8 Hz), 64.44, 64.42, 45.2 (dd, *J* = 19.3, 18.1 Hz), -4.5 (t, *J* = 2.3 Hz).

^{19}F NMR (282 MHz, $CDCl_3$) δ: -113.4 (ddd, *J* = 315.7, 29.1, 13.4 Hz, 1F), -115.2 (dddd, *J* = 315.7, 45.2, 13.8, 4.0 Hz, 1F).

HRMS (ESI): calcd for $C_{14}H_{20}F_2NaO_3Si$ (M+Na) 325.1042, found 325.1034.

3,3-Difluoro-1-(naphthalen-2-yl)-3-(trimethylsilyl)propan-1-ol (4j).

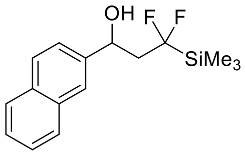

Yield 91 mg (62 %). Yellow oil.

Chromatography: hexanes/EtOAc, 10/1. R_f 0.28 (hexanes/EtOAc, 10/1).

^{1}H NMR (300 MHz, $CDCl_3$) δ: 7.90 – 7.82 (m, 4H), 7.54 – 7.48 (m, 3H), 5.44 (d, *J* = 9.0 Hz, 1H), 2.67 (br d, *J* = 5.0 Hz, 1H), 2.54-2.12 (m, 2H), 0.25 (s, 9H).

$^{13}C\ \{^1H\}$ NMR (75 MHz, $CDCl_3$) δ: 141.4, 133.5, 133.1, 130.8 (t, J = 258.6), 128.5, 128.1, 127.8, 126.3, 126.0, 124.4, 123.9, 68.8 (dd, J = 8.0, 5.8 Hz), 45.3 (dd, J = 19.4, 18.2 Hz), -4.5 (t, J = 2.3 Hz).

^{19}F NMR (282 MHz, $CDCl_3$) δ: -113.3 (ddd, J = 315.2, 29.6, 12.7 Hz, 1F), -115.2 (dddd, J = 315.2, 32.0, 13.2, 5.0 Hz, 1F).

HRMS (ESI): calcd for $C_{16}H_{20}F_2NaOSi$ (M+Na) 317.1144, found 317.1136.

3,3-Difluoro-1-(naphthalen-1-yl)-3-(trimethylsilyl)propan-1-ol (4k).

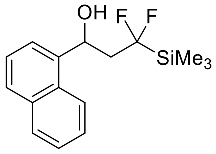

Yield 38 mg (26 %). Yellow oil.

Chromatography: hexanes/EtOAc, 10/1. R_f 0.26 (hexanes/EtOAc, 10/1).

^1H NMR (300 MHz, $CDCl_3$) δ: 8.08 (d, J = 8.1 Hz, 1H), 7.92 (d, J = 7.7 Hz, 1H), 7.85 – 7.79 (m, 2H), 7.61-7.50 (m, 3H), 6.11 (d, J = 8.2 Hz, 1H), 2.66 (dd, J = 6.4, 2.1 Hz, 1H), 2.48 – 2.29 (m, 2H), 0.24 (s, 9H).

$^{13}C\ \{^1H\}$ NMR (75 MHz, $CDCl_3$) δ: 139.5, 133.9, 131.2 (dd, J = 259.3, 258.0 Hz), 129.9, 129.1, 128.2, 126.4, 125.68, 125.70, 122.8, 122.8, 65.2 (dd, J = 8.6, 5.2 Hz), 44.7 (dd, J = 19.4, 18.0 Hz), -4.5 (t, J = 2.3 Hz).

^{19}F NMR (282 MHz, $CDCl_3$) δ: -113.8 (ddd, J = 314.9, 29.5, 13.4 Hz, 1F), -116.2 (dddd, J = 314.9, 31.8, 14.7, 6.4 Hz, 1F).

HRMS (ESI): calcd for $C_{16}H_{20}F_2NaOSi$ (M+Na) 317.1144, found 317.1141.

3,3-Difluoro-1-(pyridin-3-yl)-3-(trimethylsilyl)propan-1-ol (4l).

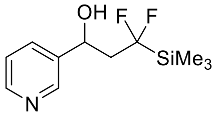

Yield 55 mg (45 %). Yellow oil.

Chromatography: EtOAc. R_f 0.30 (EtOAc).

^1H NMR (300 MHz, $CDCl_3$) δ: 8.48 (d, J = 2.2 Hz, 1H), 8.40 (dd, J = 4.8, 1.7 Hz, 1H), 7.74 (ddd, J = 8.0, 2.2, 1.7 Hz, 1H), 7.26 (dd, J = 8.0, 4.8 Hz, 1H), 5.23 (dd, J = 8.7, 3.2 Hz, 1H), 4.03 (br s, 1H), 2.45 – 1.99 (m, 2H), 0.19 (s, 9H).

$^{13}C\ \{^1H\}$ NMR (75 MHz, $CDCl_3$) δ: 148.6, 147.5, 140.0, 133.8, 130.3 (t, J = 258.7 Hz), 123.6, 66.3 (dd, J = 8.2, 5.9 Hz), 45.1 (dd, J = 19.6, 18.5 Hz), -4.6 (t, J = 2.3 Hz).

^{19}F NMR (282 MHz, $CDCl_3$) δ: -113.2 (ddd, J = 316.3, 29.9, 11.7 Hz, 1F), -115.2 (ddd, J = 316.3, 32.2, 12.9 Hz, 1F).

HRMS (ESI): calcd for $C_{11}H_{18}F_2NOSi$ (M+H) 246.1120, found 246.1127.

3,3-Difluoro-1-(furan-2-yl)-3-(trimethylsilyl)propan-1-ol (4m).

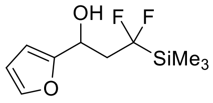

Yield 81 mg (69 %). Yellow oil.

Chromatography: hexanes/EtOAc, 4/1. R_f 0.28 (hexanes/EtOAc, 4/1).

^{1}H NMR (300 MHz, $CDCl_3$) δ: 7.37 (dd, *J* = 1.8, 0.8 Hz, 1H), 6.33 (dd, *J* = 3.2, 1.8 Hz, 1H), 6.27 (dd, *J* = 3.2, 0.8 Hz, 1H), 5.21 (d, *J* = 8.4 Hz, 1H), 2.52-2.20 (m, 3H), 0.20 (s, 9H).

^{13}C {^{1}H} NMR (75 MHz, $CDCl_3$) δ: 155.8, 142.2, 130.3 (t, *J* = 259.2 Hz), 110.4, 106.1, 62.5 (dd, *J* = 8.6, 6.5 Hz), 41.5(dd, *J* = 19.5, 18.5 Hz), -4.6 (t, *J* = 2.3 Hz).

^{19}F NMR (282 MHz, $CDCl_3$) δ: -113.4 (ddd, *J* = 316.6, 27.5, 14.3 Hz, 1F), -115.5 (dddd, *J* = 316.6, 29.8, 15.3, 3.6 Hz, 1F).

HRMS (ESI): calcd for $C_{10}H_{16}F_2NaO_2Si$ (M+Na) 257.0780, found 257.0784.

3,3-Difluoro-1-(thiophen-2-yl)-3-(trimethylsilyl)propan-1-ol (4n).

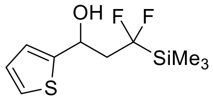

Yield 70 mg (56 %). Colorless oil.

Chromatography: hexanes/EtOAc, 6/1. R_f 0.34 (hexanes/EtOAc, 6/1).

^{1}H NMR (300 MHz, $CDCl_3$) δ: 7.27 (dd, *J* = 4.8, 1.5 Hz, 1H), 7.03 – 6.97 (m, 2H), 5.52 (d, *J* = 8.8 Hz, 1H), 2.65 (br s, 1H), 2.57 – 2.18 (m, 2H), 0.23 (s, 9H).

^{13}C {^{1}H} NMR (75 MHz, $CDCl_3$) δ: 147.9, 130.4 (t, *J* = 258.8 Hz), 126.8, 124.7, 123.5, 65.0 (dd, *J* = 8.7, 6.2 Hz), 45.4 (dd, *J* = 19.3, 18.3 Hz), -4.6 (t, *J* = 2.3 Hz).

^{19}F NMR (282 MHz, $CDCl_3$) δ: -113.8 (ddd, *J* = 316.3, 25.3, 16.3 Hz, 1F), -115.2 (dddd, *J* = 316.3, 33.7, 12.8, 4.6 Hz, 1F).

HRMS (ESI): calcd for $C_{10}H_{16}F_2NaO_2SSi$ (M+Na) 273.0551, found 273.0548.

1-(Benzofuran-2-yl)-3,3-difluoro-3-(trimethylsilyl)propan-1-ol (4o).

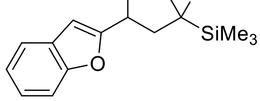

Yield 53 mg (37 %). Yellow oil.

Chromatography: hexanes/EtOAc, from 10/1 to 5/1. R_f 0.35 (hexanes/EtOAc, 5/1).

^{1}H NMR (300 MHz, $CDCl_3$) δ: 7.57 (d, *J* = 7.6 Hz, 1H), 7.50 (d, *J* = 7.9 Hz, 1H), 7.34 – 7.21 (m, 2H), 6.70 (s, 1H), 5.45-5.39 (m, 1H), 2.65 (br s, 1H), 2.58 – 2.39 (m, 2H), 0.26 (s, 9H).

^{13}C {^{1}H} NMR (75 MHz, $CDCl_3$) δ: 158.4, 155.0, 130.4 (d, *J* = 259.6, 258.7 Hz), 128.2, 124.4, 123.0, 121.3, 111.4, 102.9, 63.2 (dd, *J* = 8.5, 6.8 Hz), 41.6 (t, *J* = 19.0 Hz), -4.6 (t, *J* = 2.3 Hz)

^{19}F NMR (282 MHz, $CDCl_3$) δ: -114.0 (ddd, *J* = 315.7, 26.1, 15.9 Hz, 1F), -115.4 (dddd, *J* = 315.7, 27.2, 16.8, 4.5 Hz, 1F).

HRMS (ESI): calcd for $C_{14}H_{18}F_2NaO_2Si$ (M+Na) 307.0936, found 307.0932.

3,3-Difluoro-1-(1-methyl-1*H*-pyrrol-2-yl)-3-(trimethylsilyl)propan-1-one (3p).

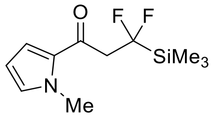

Yield 39 mg (32 %). Yellow oil.

Chromatography: hexanes/EtOAc, from 7/1 to 5/1. R_f 0.21 (hexanes/EtOAc, 7/1).

^{1}H NMR (300 MHz, $CDCl_3$) δ: 6.98 (dd, *J* = 4.2, 1.5 Hz, 1H), 6.83 (dd, *J* = 2.5, 1.5 Hz, 1H), 6.14 (dd, *J* = 4.2, 2.5 Hz, 1H), 3.93 (s, 3H), 3.29 (t, *J* = 20.3 Hz, 2H), 0.21 (s, 9H).

^{13}C {^{1}H} NMR (75 MHz, $CDCl_3$) δ: 184.4 (t, *J* = 7.4 Hz), 132.1, 128.5 (t, *J* = 261.9 Hz), 121.4, 108.4, 46.9 (t, *J* = 21.8 Hz), 37.9, -3.8 (t, *J* = 2.4 Hz).

^{19}F NMR (282 MHz, $CDCl_3$) δ: -107.7 (t, *J* = 20.1 Hz).

HRMS (ESI): calcd for $C_{11}H_{18}F_2NOSi$ (M+H) 246.1120, found 246.1125.

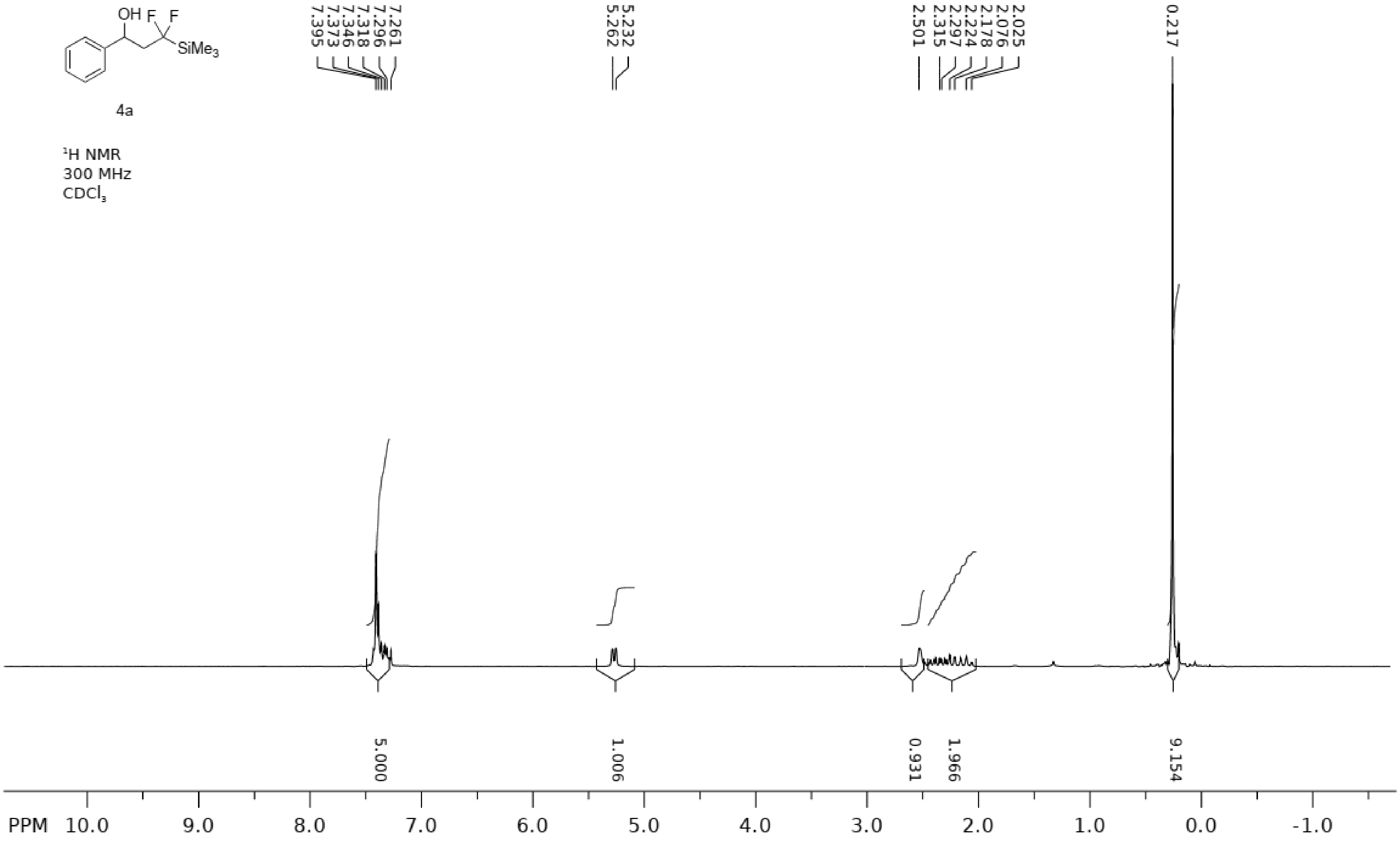

OH F F
SiMe3
4a
1H NMR
300 MHz
CDCl3
7.395
7.373
7.346
7.318
7.296
7.261
5.232
5.262
2.501
2.315
2.297
2.224
2.178
2.076
2.025
0.217
5.000
1.006
0.931
1.966
9.154
PPM 10.0 9.0 8.0 7.0 6.0 5.0 4.0 3.0 2.0 1.0 0.0 -1.0

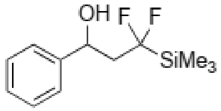

4a

$^{13}C\{^{1}H\}$ NMR
75 MHz
$CDCl_3$

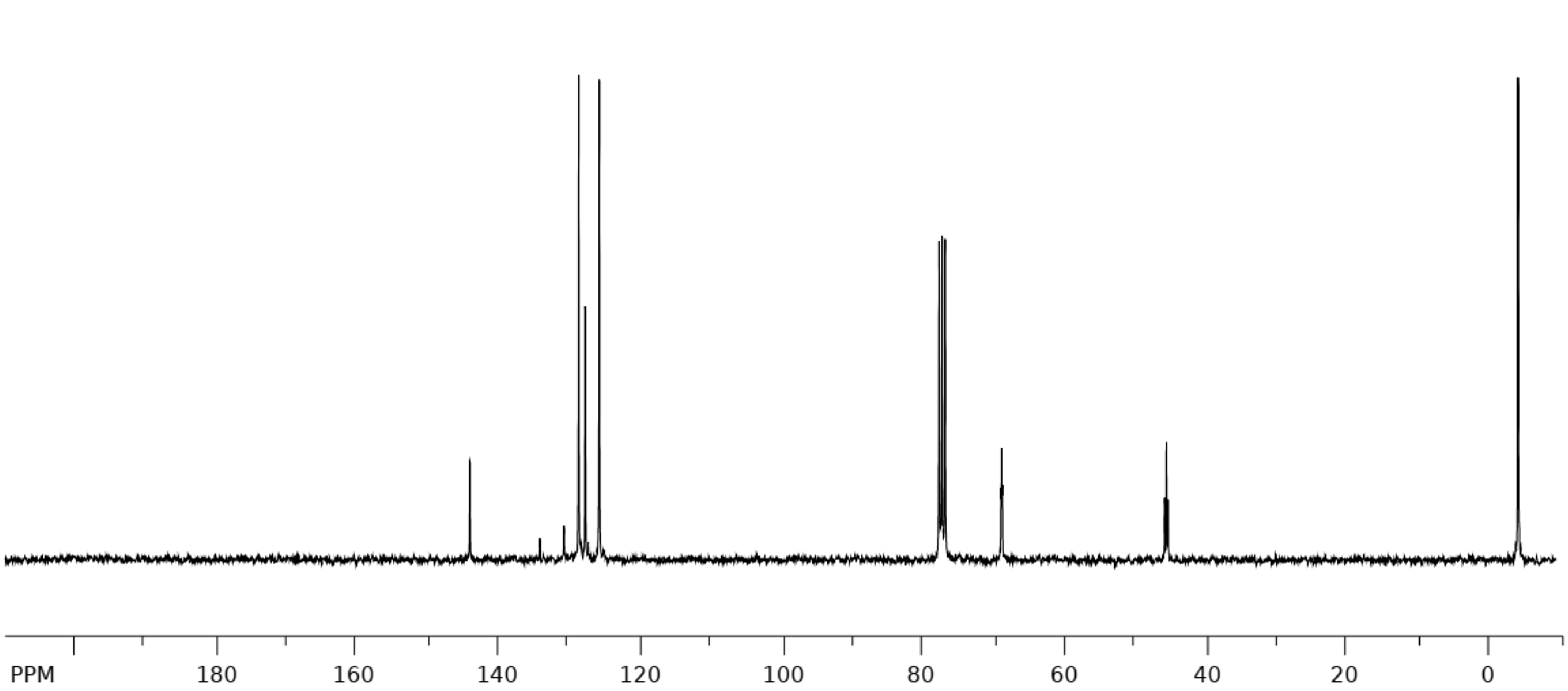

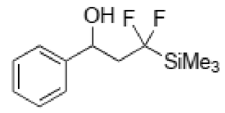

4a

^{19}F NMR
282 MHz
$CDCl_3$

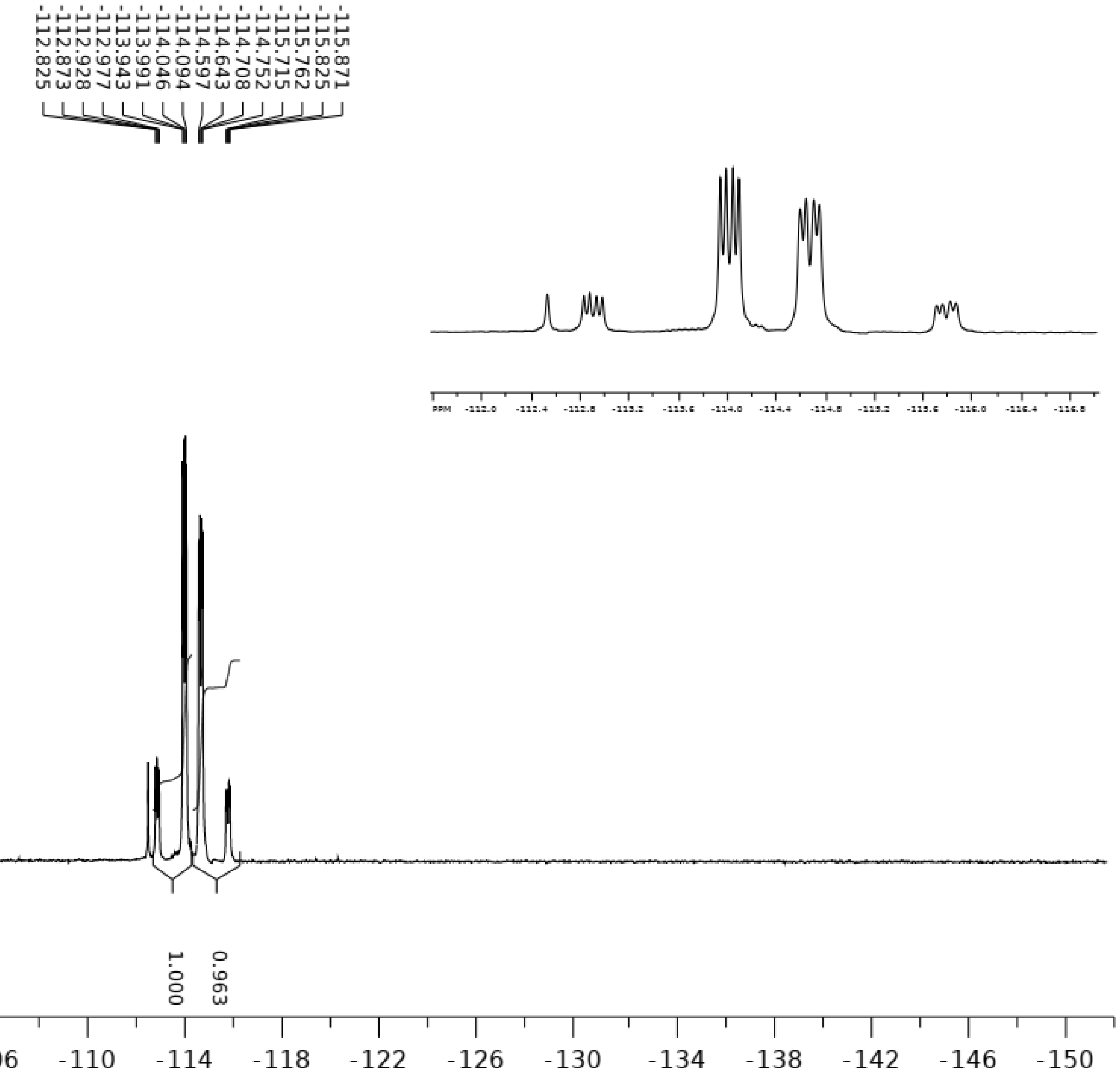

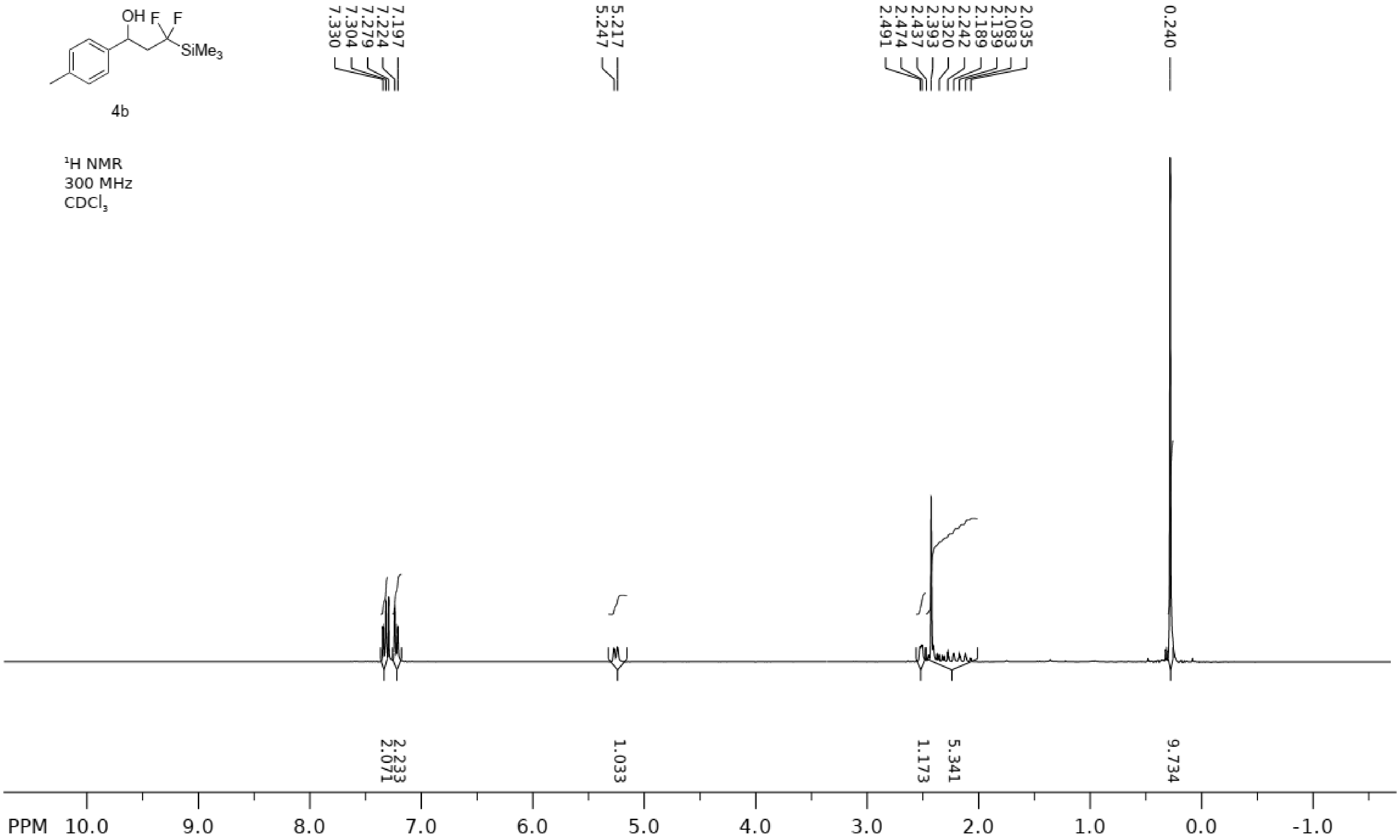
OH F F
SiMe3
4b
1H NMR
300 MHz
CDCl3
7.330
7.304
7.279
7.224
7.197
5.247
5.217
2.491
2.474
2.437
2.393
2.320
2.242
2.189
2.139
2.083
2.035
0.240
2.071
2.233
1.033
1.173
5.341
9.734
PPM 10.0 9.0 8.0 7.0 6.0 5.0 4.0 3.0 2.0 1.0 0.0 -1.0

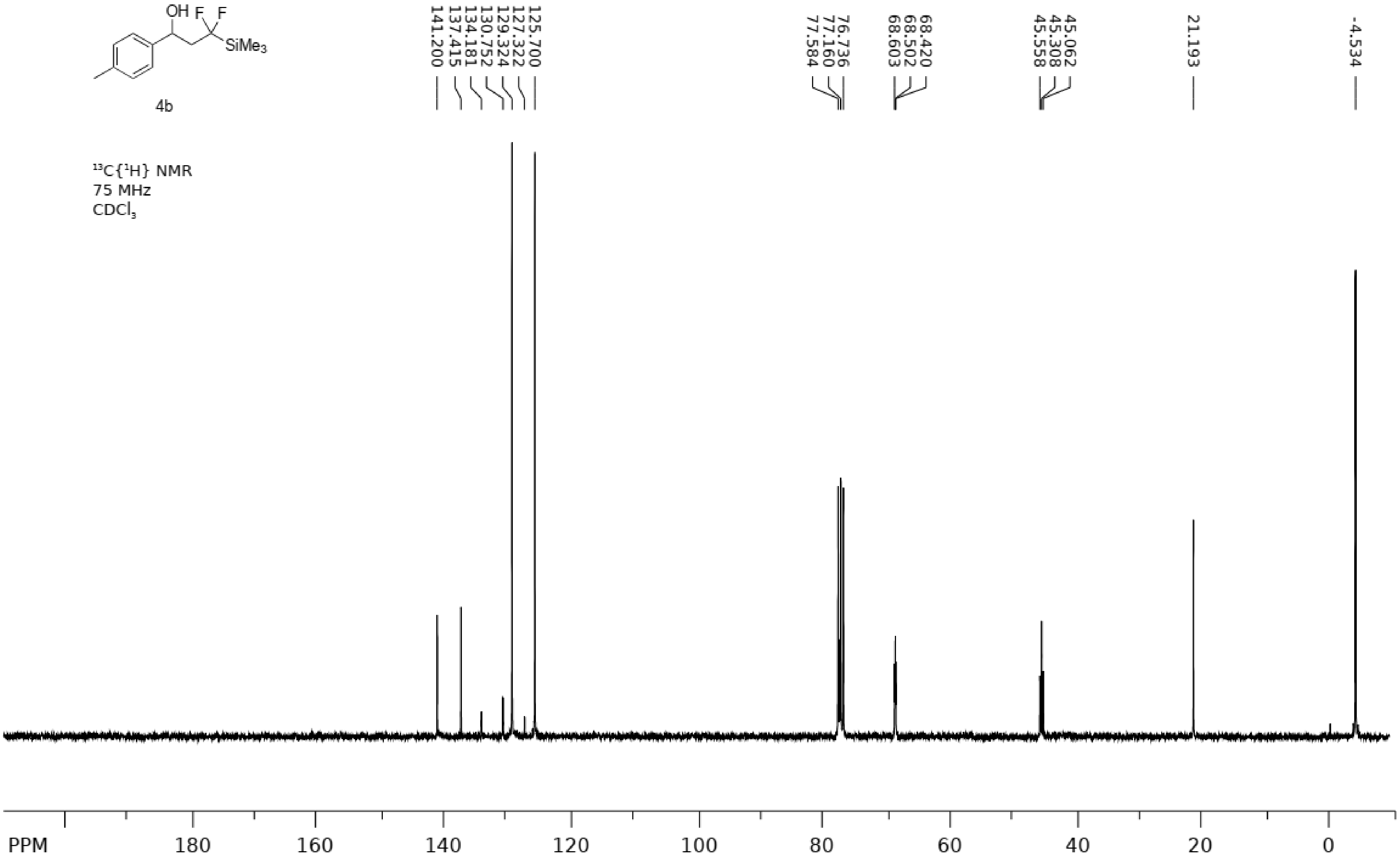
OH F F
SiMe3
4b
13C{1H} NMR
75 MHz
CDCl3
141.200
137.415
134.181
130.752
129.324
127.322
125.700
77.584
77.160
76.736
68.603
68.502
68.420
45.558
45.308
45.062
21.193
-4.534
PPM
180
160
140
120
100
80
60
40
20
0

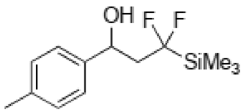

4b

^{19}F NMR
282 MHz
$CDCl_3$

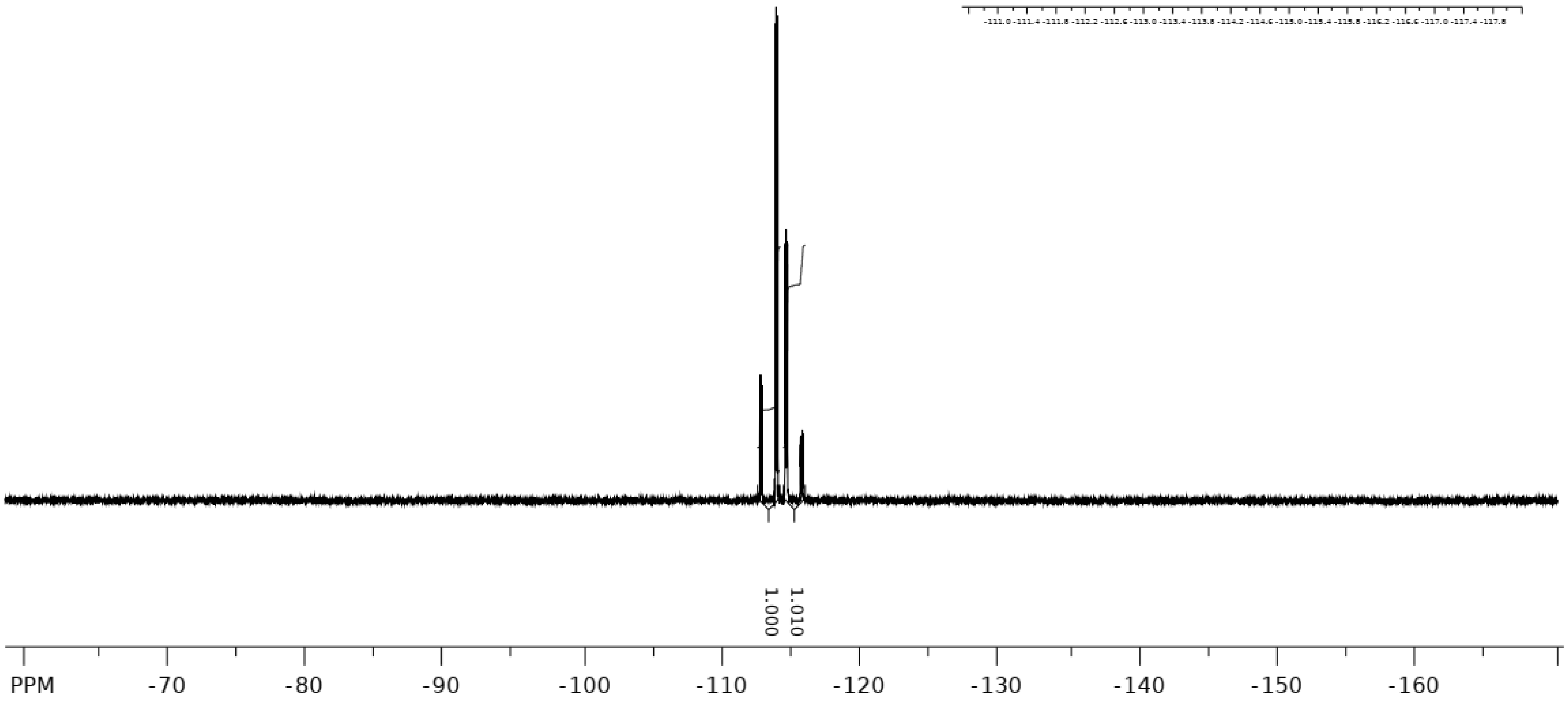

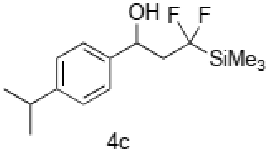

4c

^{1}H NMR
300 MHz
$CDCl_3$

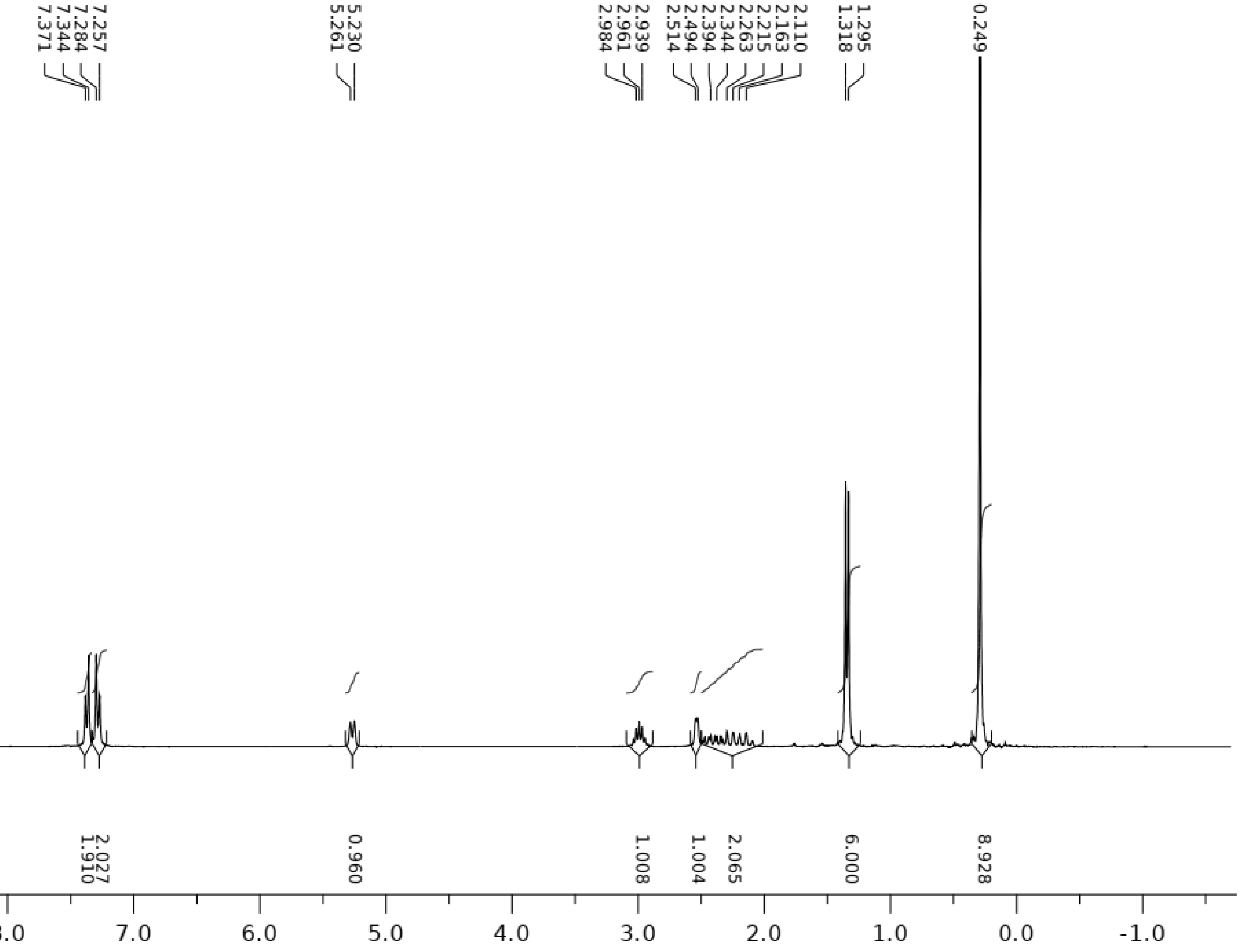

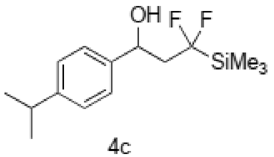

4c

$^{13}C\{^{1}H\}$ NMR
75 MHz
$CDCl_3$

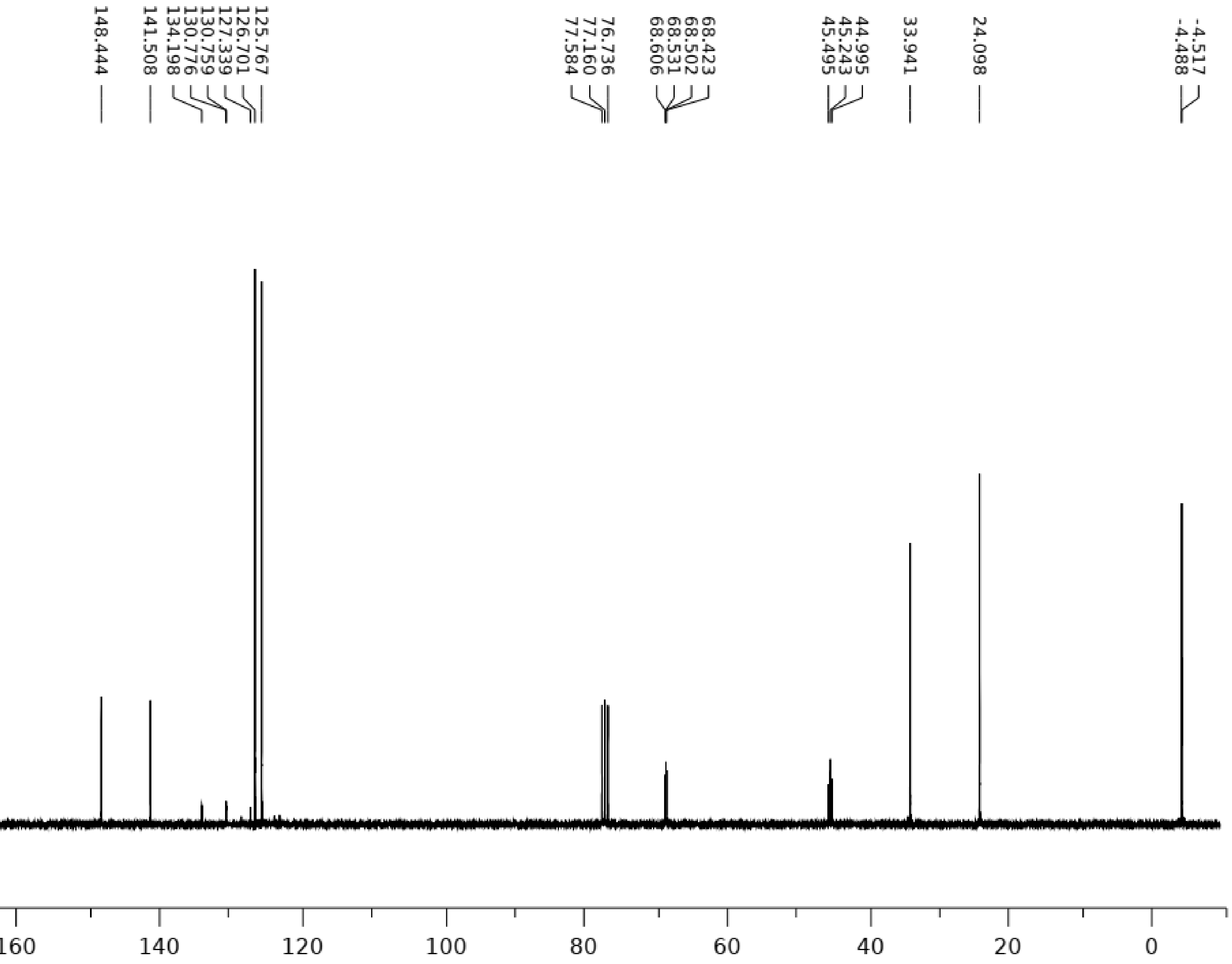

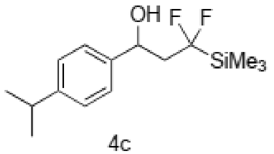

4c

^{19}F NMR
282 MHz
$CDCl_3$

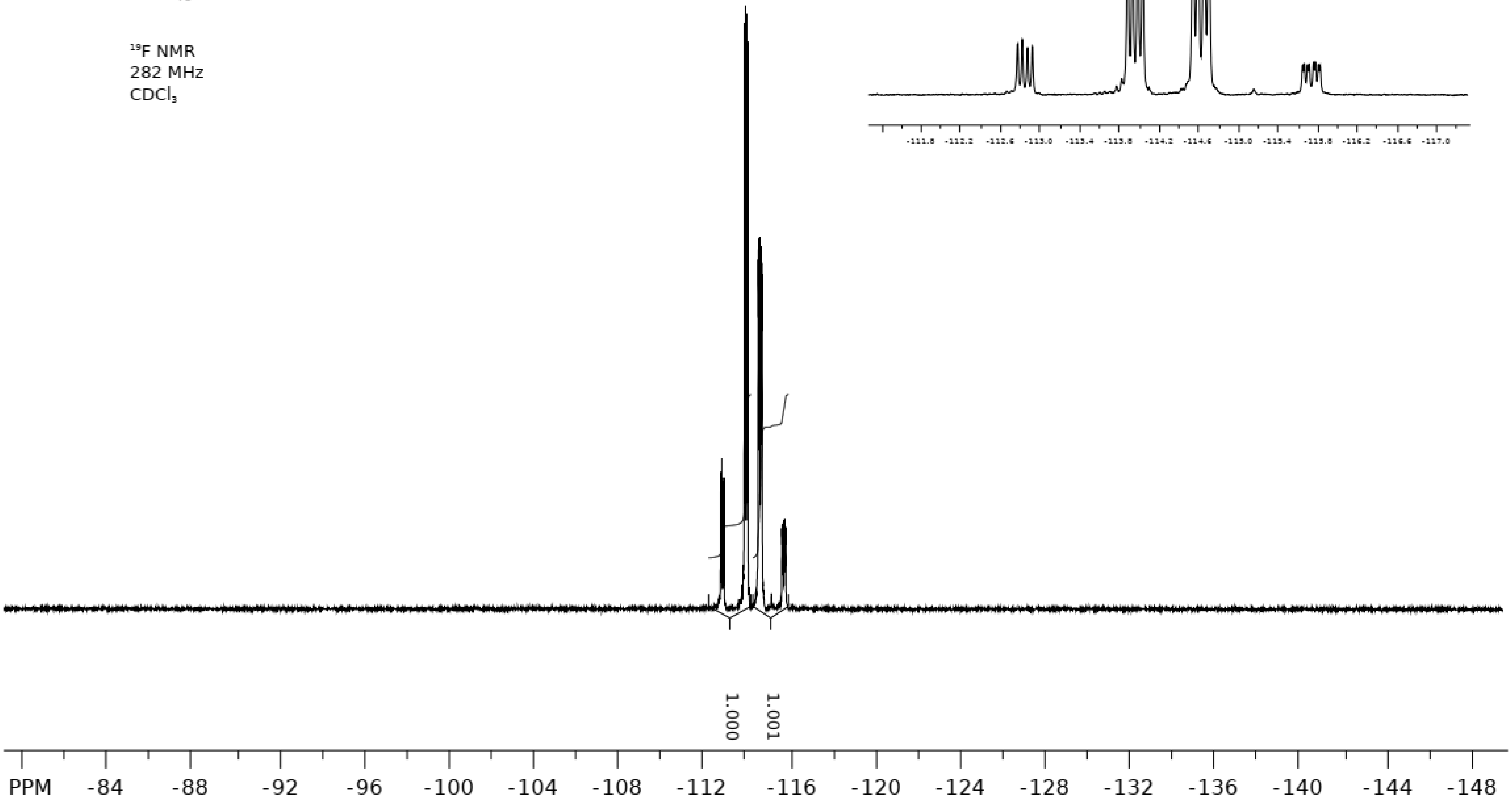

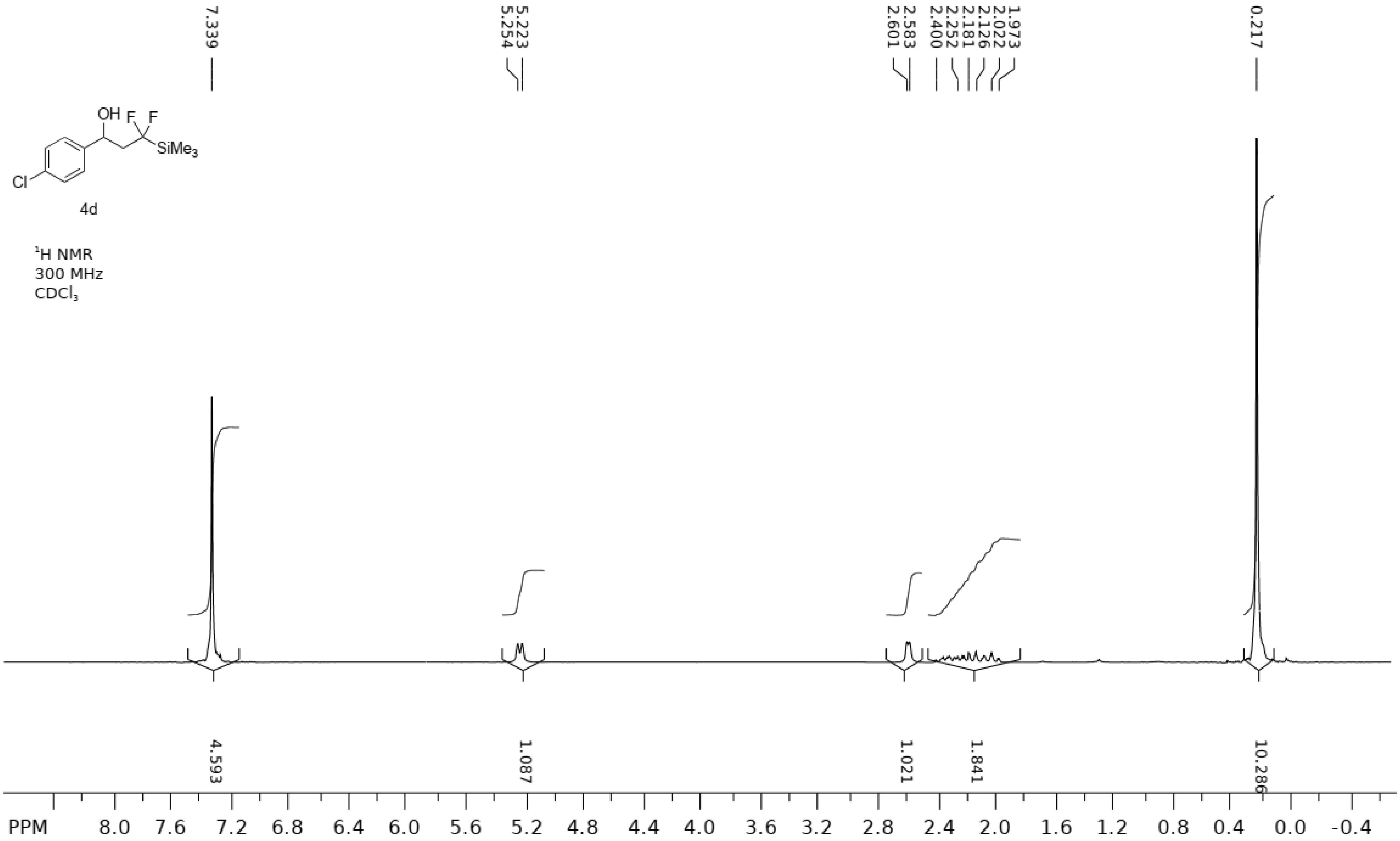

OH
F
F
SiMe3
Cl
4d
1H NMR
300 MHz
CDCl3
7.339
5.223
5.254
2.583
2.601
2.400
2.252
2.181
2.126
2.022
1.973
0.217
4.593
1.087
1.021
1.841
10.286
PPM
8.0
7.6
7.2
6.8
6.4
6.0
5.6
5.2
4.8
4.4
4.0
3.6
3.2
2.8
2.4
2.0
1.6
1.2
0.8
0.4
0.0
-0.4

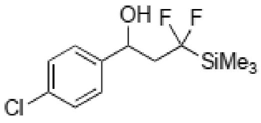

4d

$^{13}C\{^{1}H\}$ NMR
75 MHz
$CDCl_3$

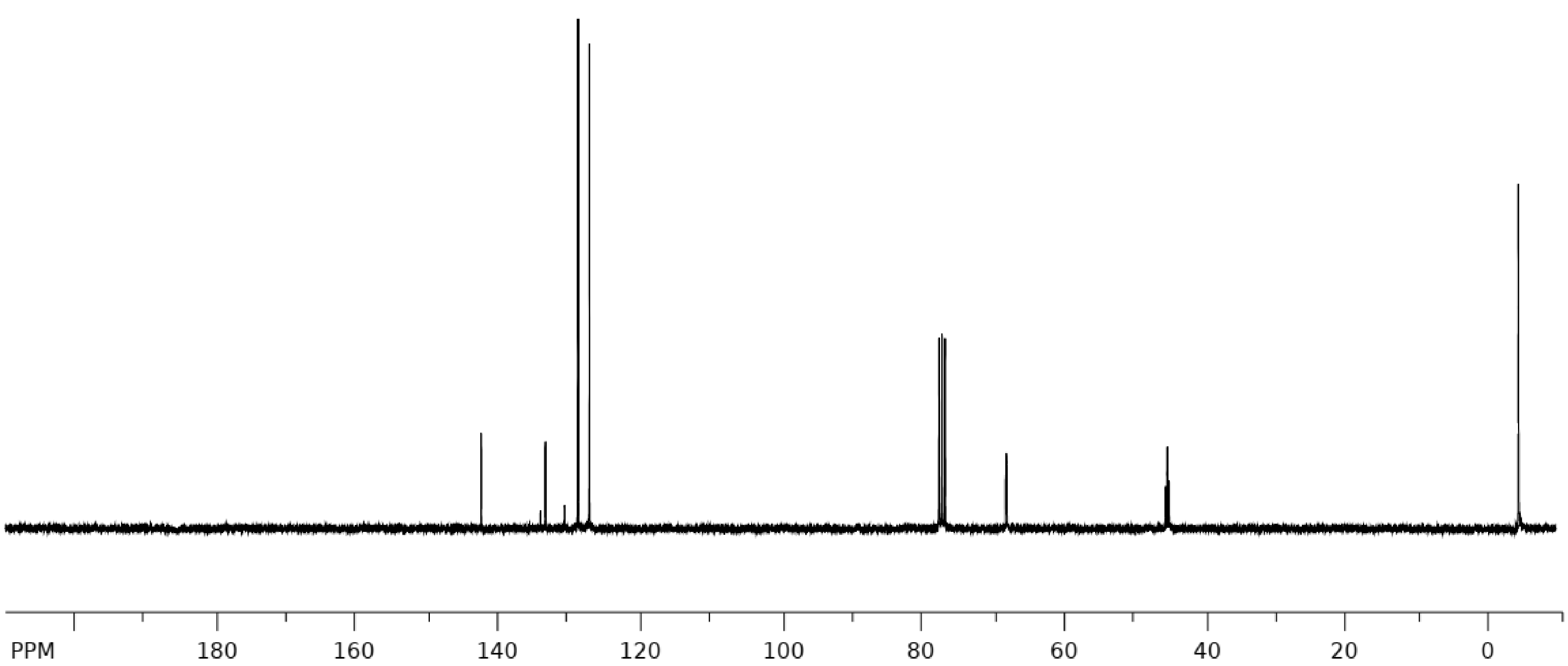

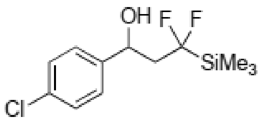

4d

^{19}F NMR
282 MHz
$CDCl_3$

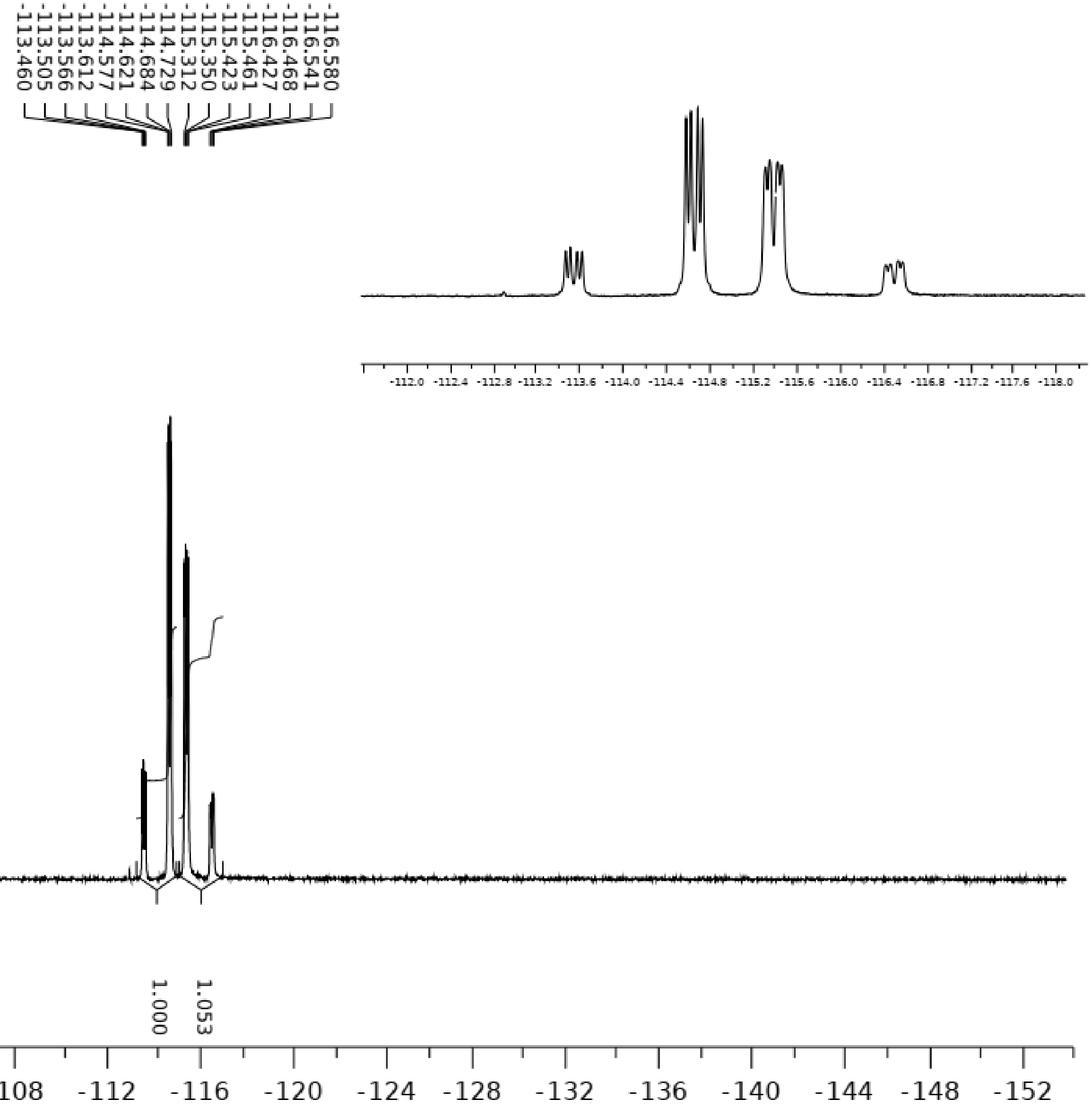

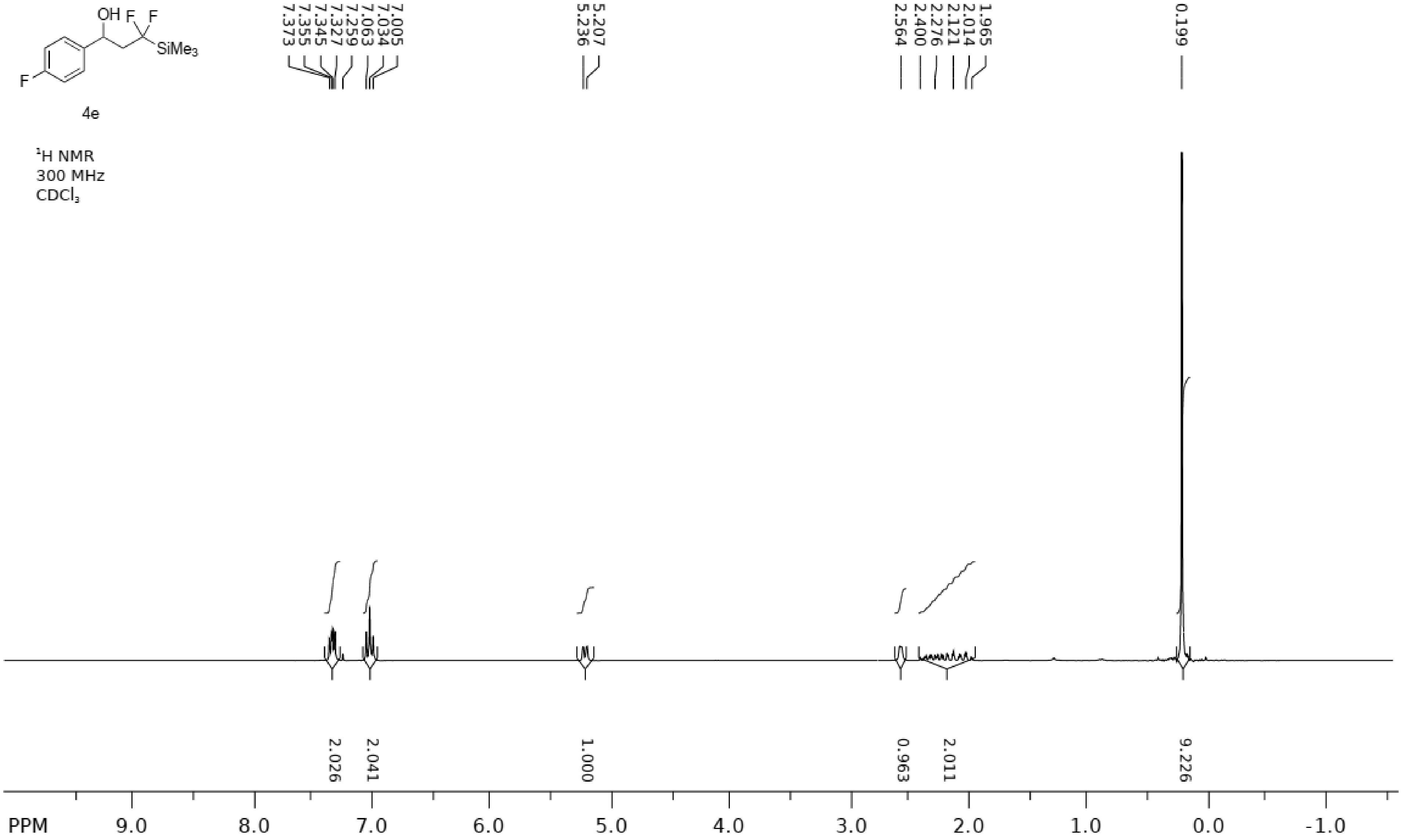

OH
F
F
SiMe3
4e
¹H NMR
300 MHz
$CDCl_3$
7.373
7.355
7.345
7.327
7.259
7.063
7.034
7.005
5.236
5.207
2.564
2.400
2.276
2.121
2.014
1.965
0.199
2.026
2.041
1.000
0.963
2.011
9.226
PPM
9.0
8.0
7.0
6.0
5.0
4.0
3.0
2.0
1.0
0.0
-1.0

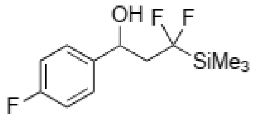

4e

$^{13}C\{^{1}H\}$ NMR
75 MHz
$CDCl_3$

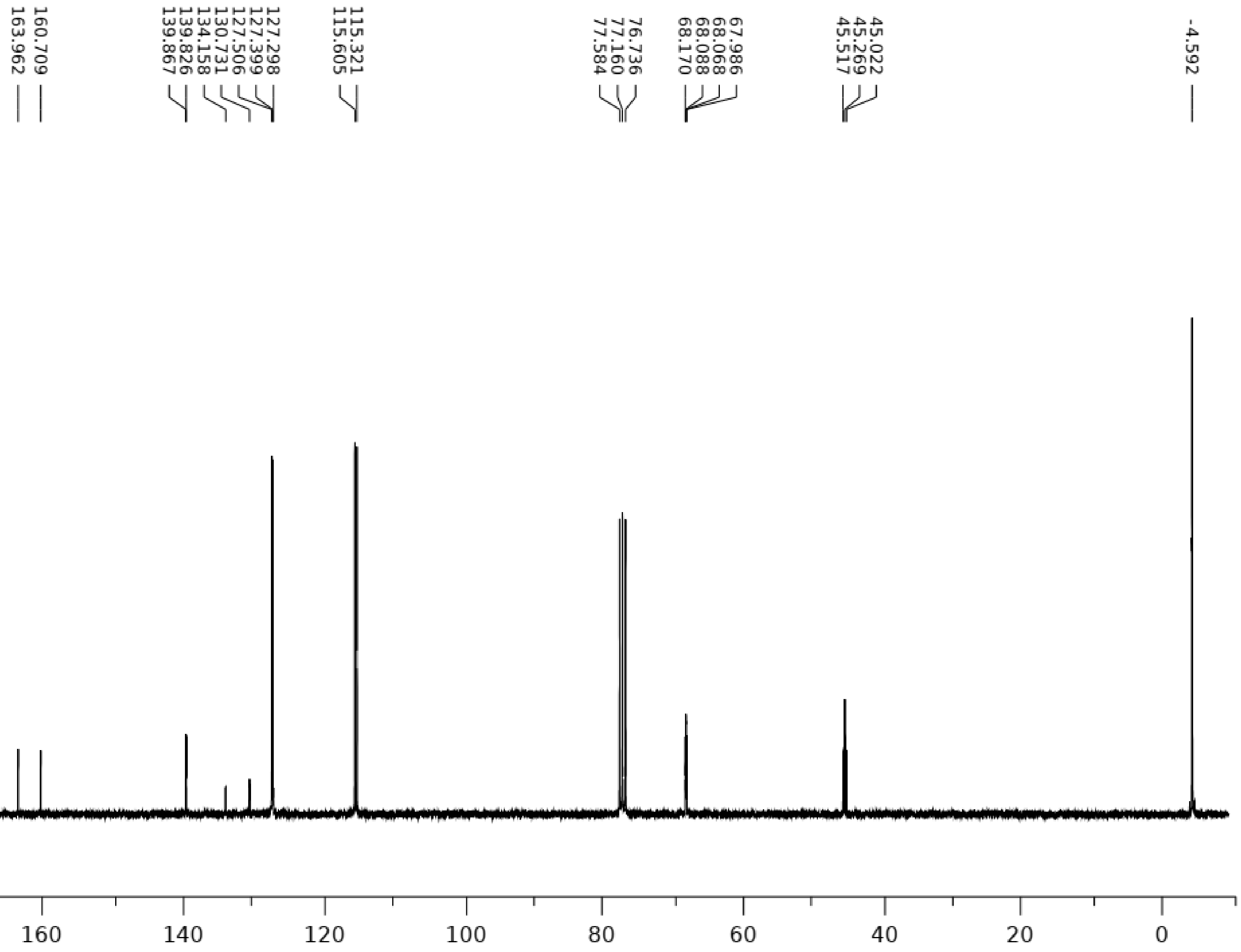

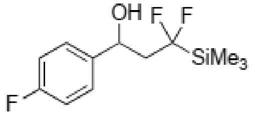

4e

^{19}F NMR
282 MHz
$CDCl_3$

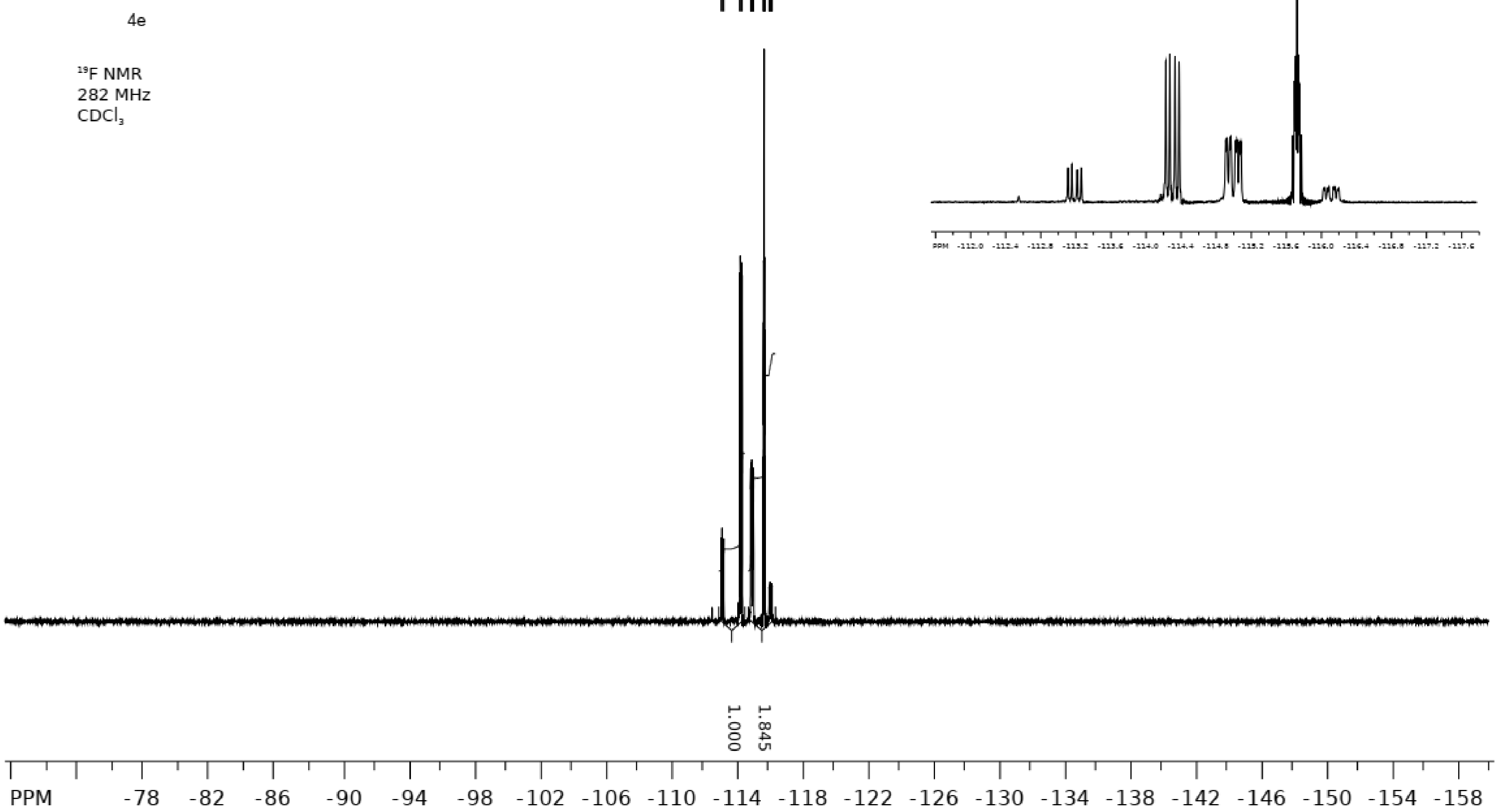

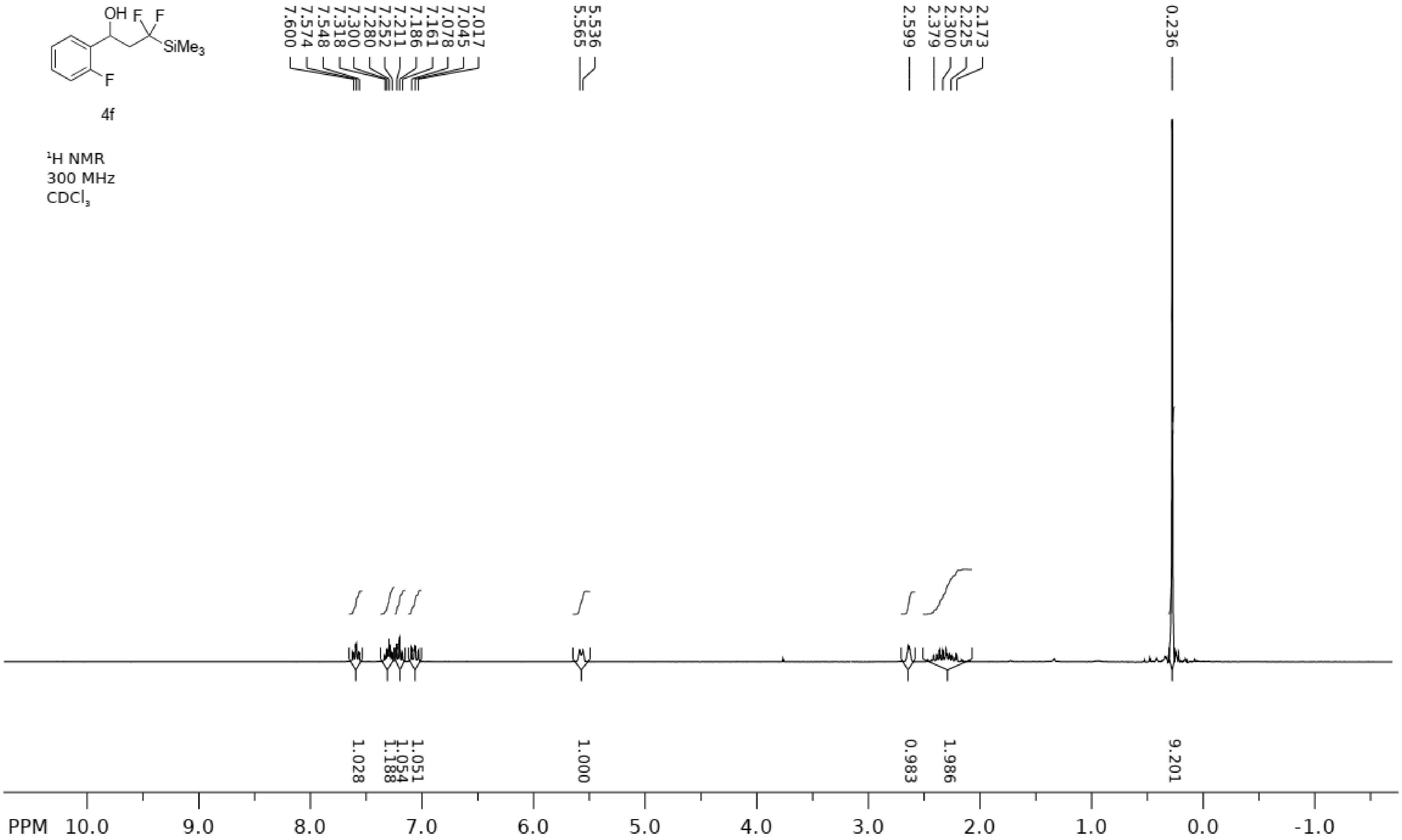
OH F F
SiMe3
F
4f
¹H NMR
300 MHz
CDCl3
7.600 7.574 7.548 7.318 7.300 7.280 7.252 7.211 7.186 7.161 7.078 7.045 7.017
5.536 5.565
2.599 2.379 2.300 2.225 2.173
0.236
1.028 1.188 1.054 1.051
1.000
0.983 1.986
9.201
PPM 10.0 9.0 8.0 7.0 6.0 5.0 4.0 3.0 2.0 1.0 0.0 -1.0

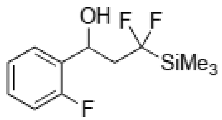

4f

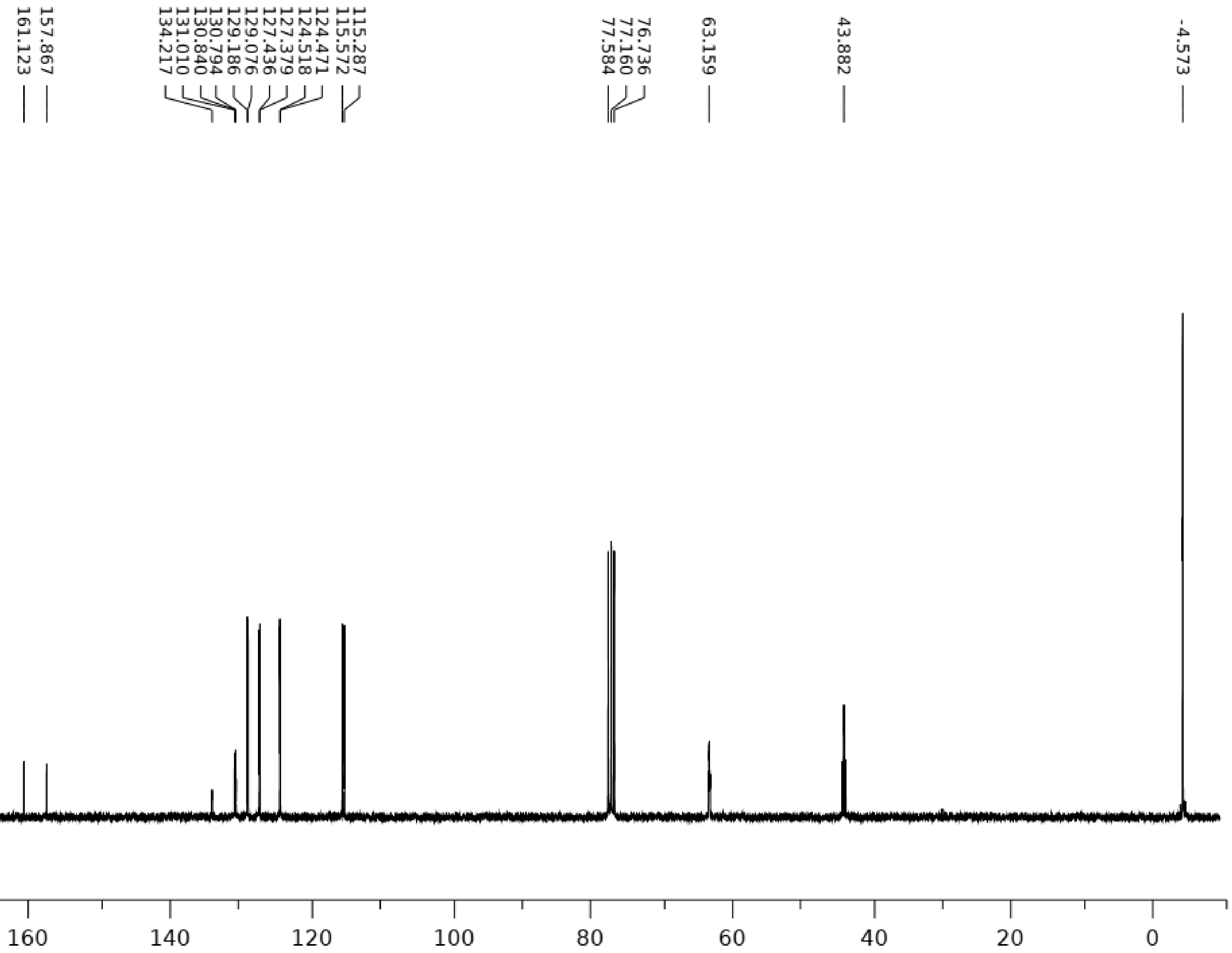

13C{1H} NMR
75 MHz
CDCl3
161.123
157.867
134.217
131.010
130.840
130.794
129.186
129.076
127.436
127.379
124.518
124.471
115.572
115.287
77.584
77.160
76.736
63.159
43.882
-4.573
PPM
180
160
140
120
100
80
60
40
20
0

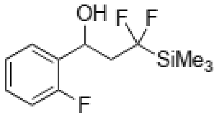

4f

^{19}F NMR
282 MHz
$CDCl_3$

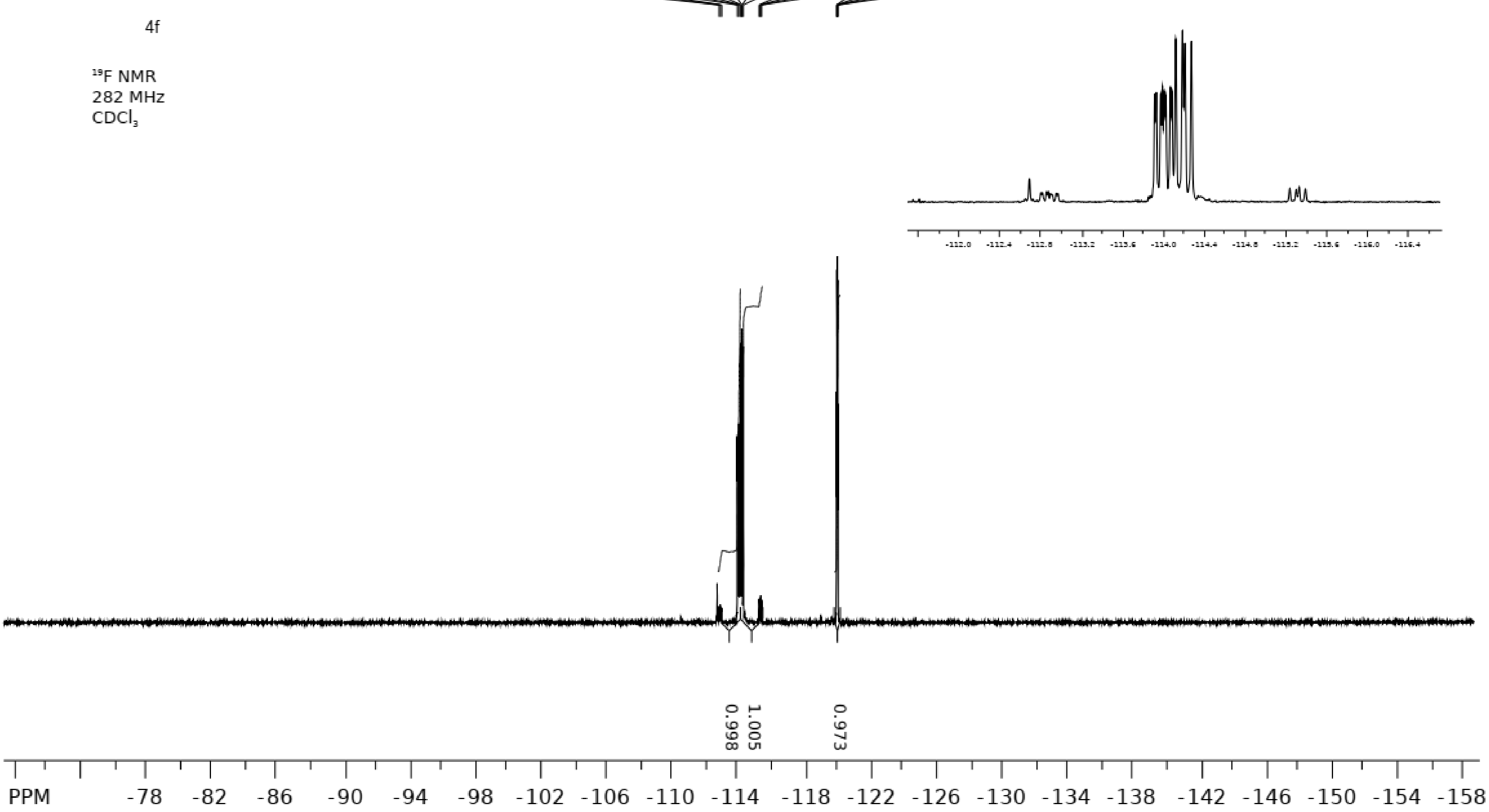

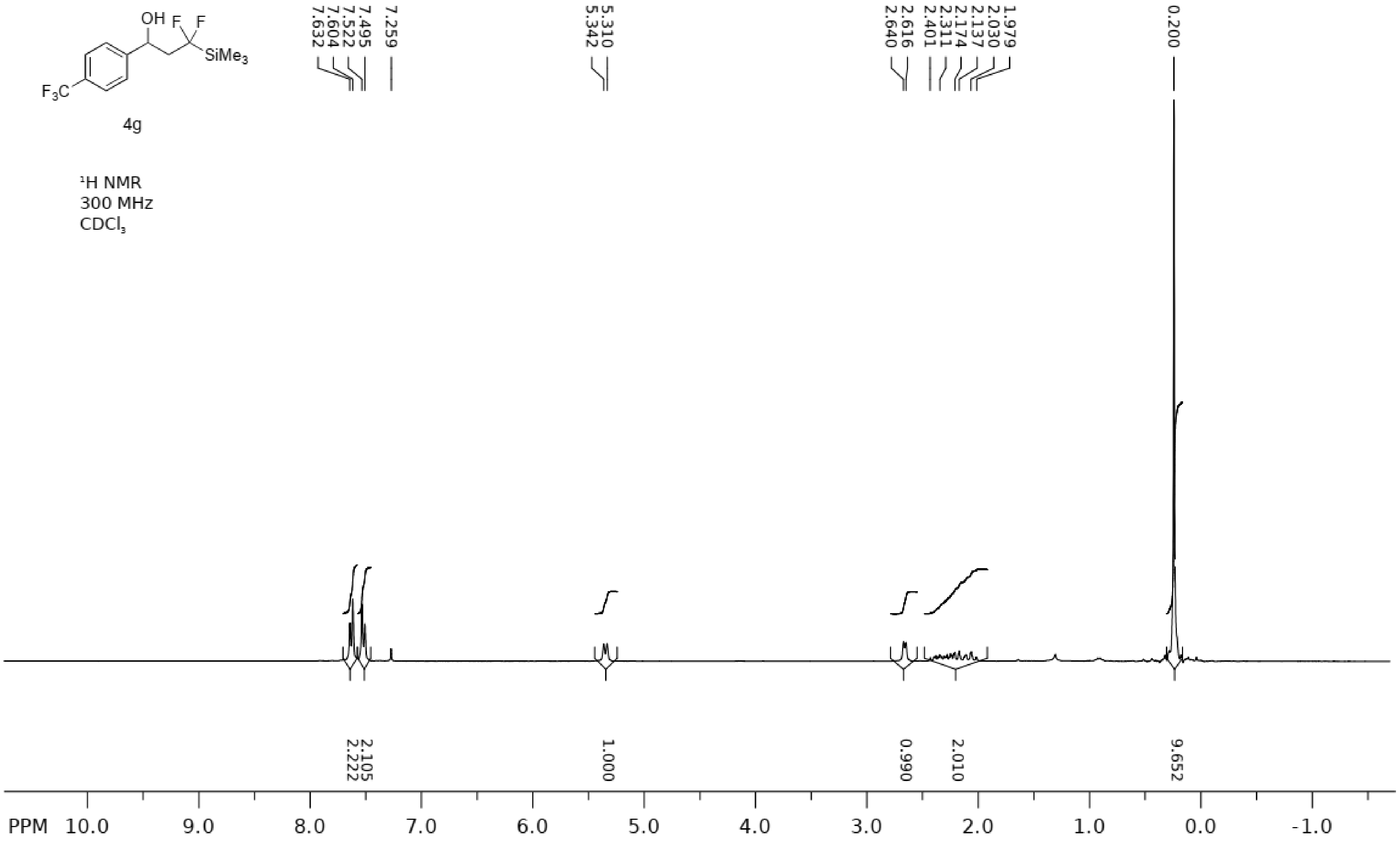
4g
^{1}H NMR
300 MHz
$CDCl_3$
7.632
7.604
7.522
7.495
7.259
5.342
5.310
2.640
2.616
2.401
2.311
2.174
2.137
2.030
1.979
0.200
2.222
2.105
1.000
0.990
2.010
9.652
PPM 10.0 9.0 8.0 7.0 6.0 5.0 4.0 3.0 2.0 1.0 0.0 -1.0

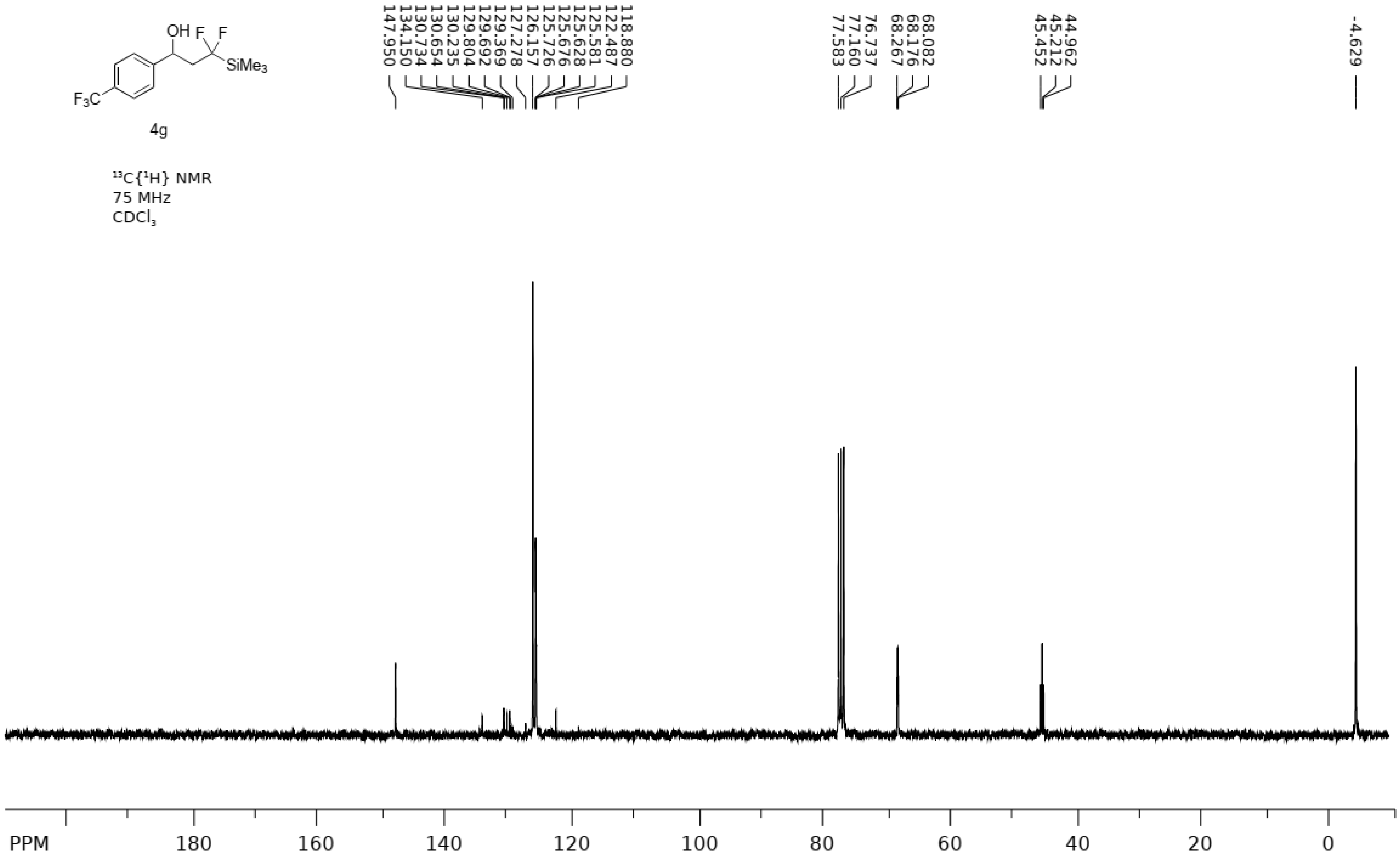
OH F F
SiMe3
F3C
4g
13C{1H} NMR
75 MHz
CDCl3
147.950
134.150
130.734
130.654
130.235
129.804
129.692
129.369
127.278
126.157
125.726
125.676
125.628
125.581
122.487
118.880
77.583
77.160
76.737
68.267
68.176
68.082
45.452
45.212
44.962
-4.629
PPM
180
160
140
120
100
80
60
40
20
0

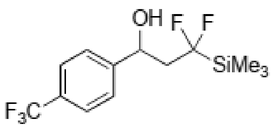

4g

^{19}F NMR
282 MHz
$CDCl_3$

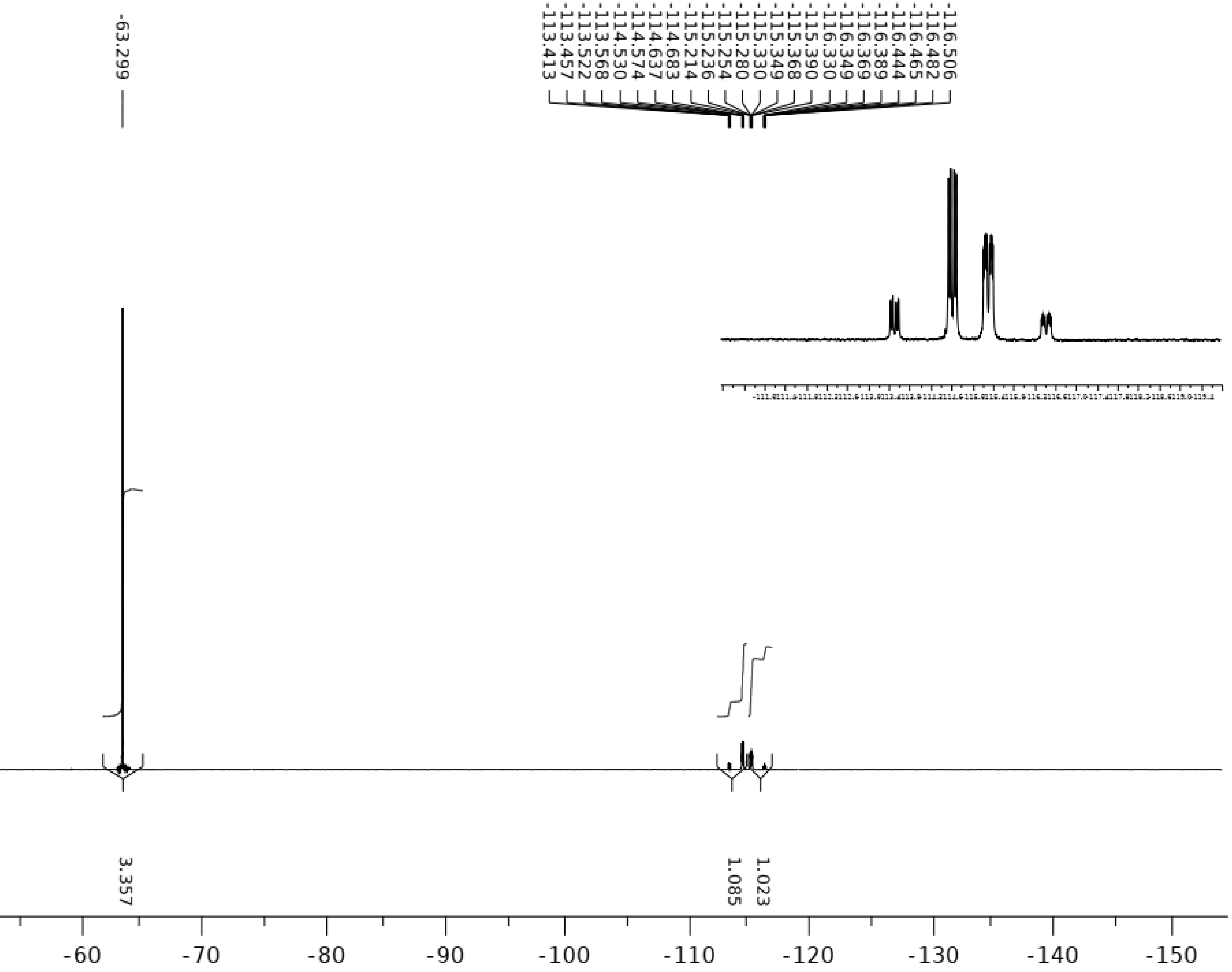

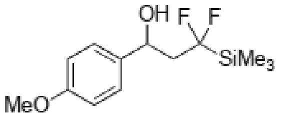

4h

^{1}H NMR
300 MHz
$CDCl_3$

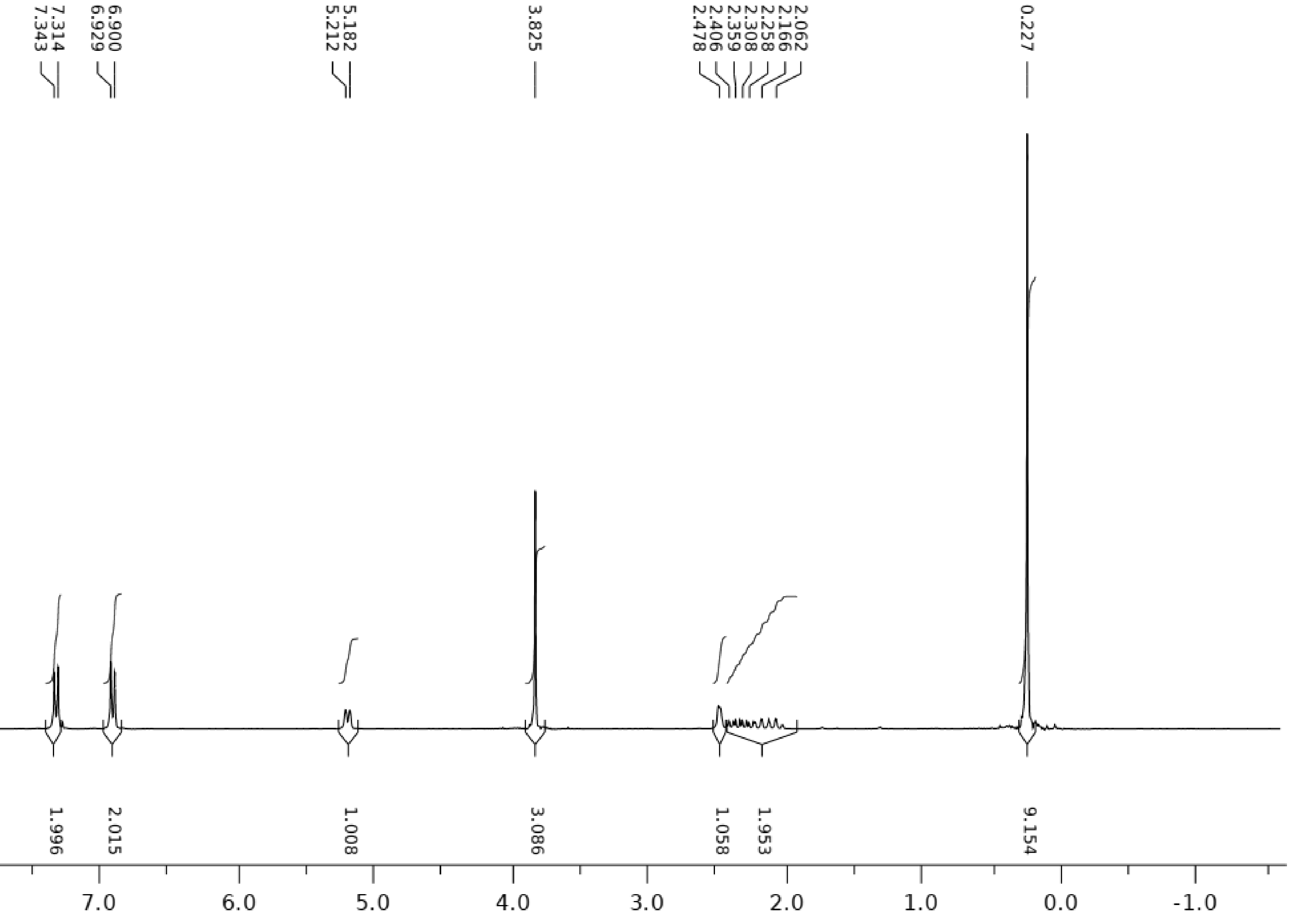

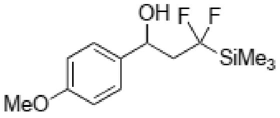

4h

$^{13}C\{^1H\}$ NMR
75 MHz
$CDCl_3$

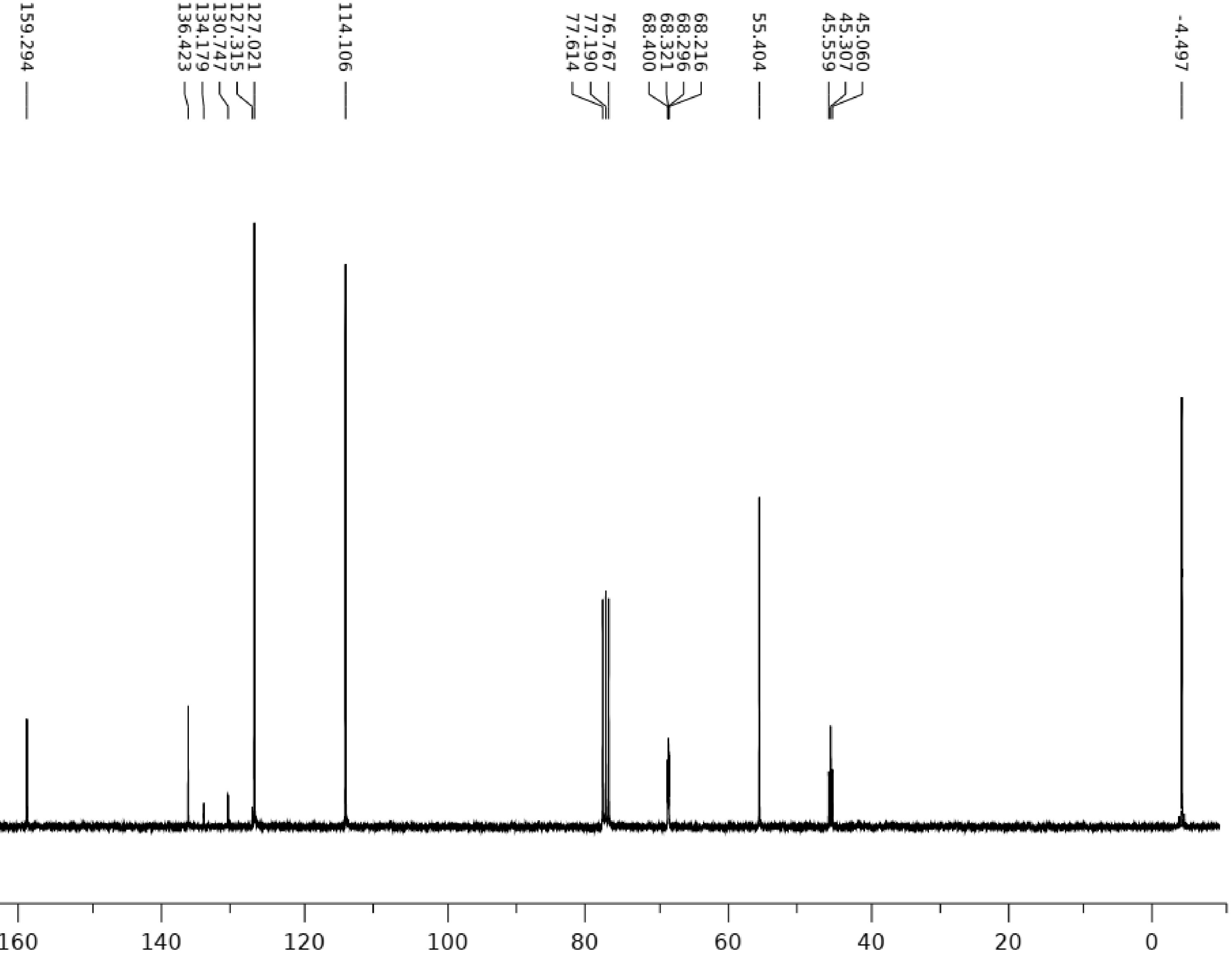

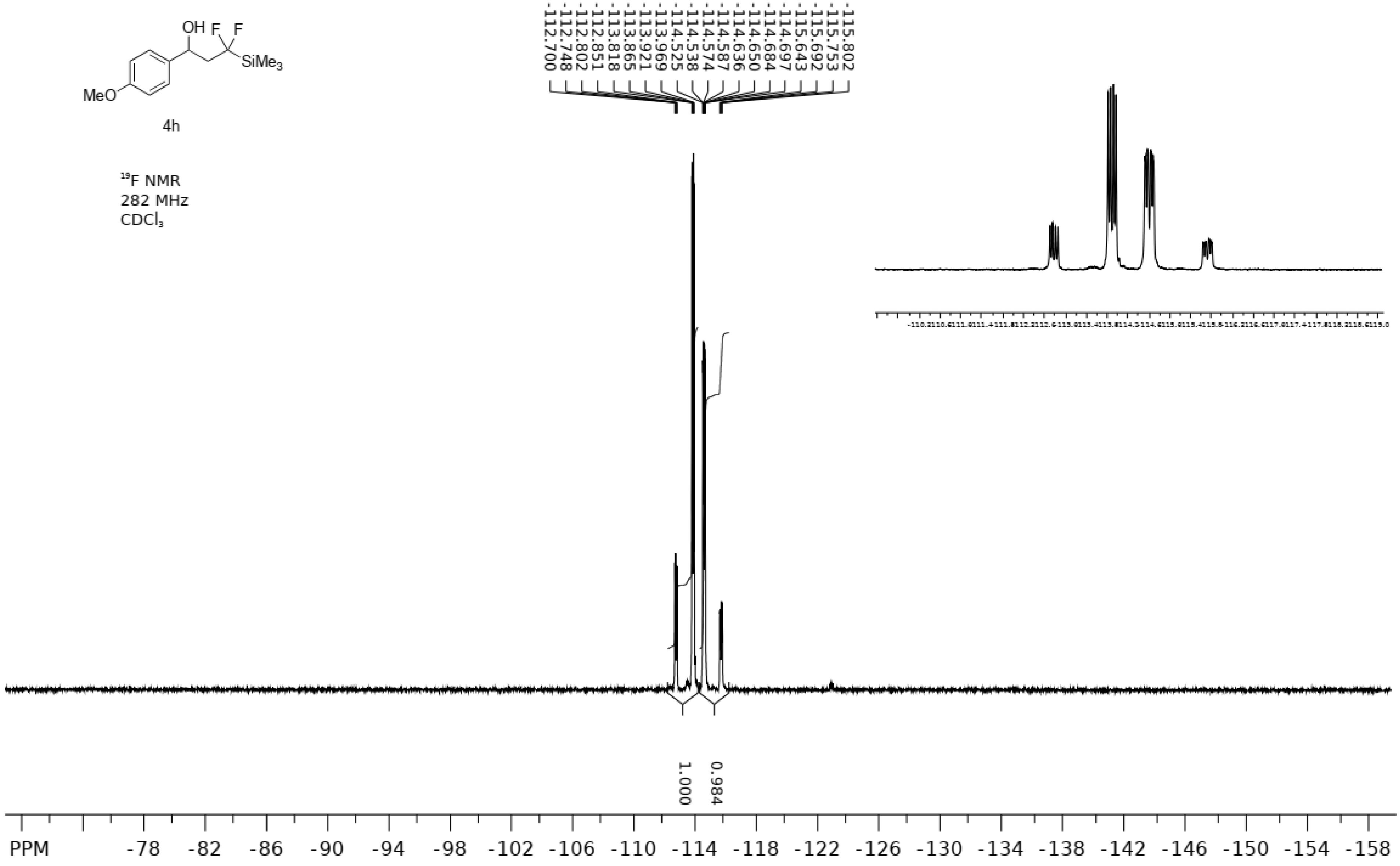

4h
19F NMR
282 MHz
CDCl3
-112.700
-112.748
-112.802
-112.851
-113.818
-113.865
-113.921
-113.969
-114.525
-114.538
-114.574
-114.587
-114.636
-114.650
-114.684
-114.697
-115.643
-115.692
-115.753
-115.802
1.000
0.984
PPM
-78 -82 -86 -90 -94 -98 -102 -106 -110 -114 -118 -122 -126 -130 -134 -138 -142 -146 -150 -154 -158

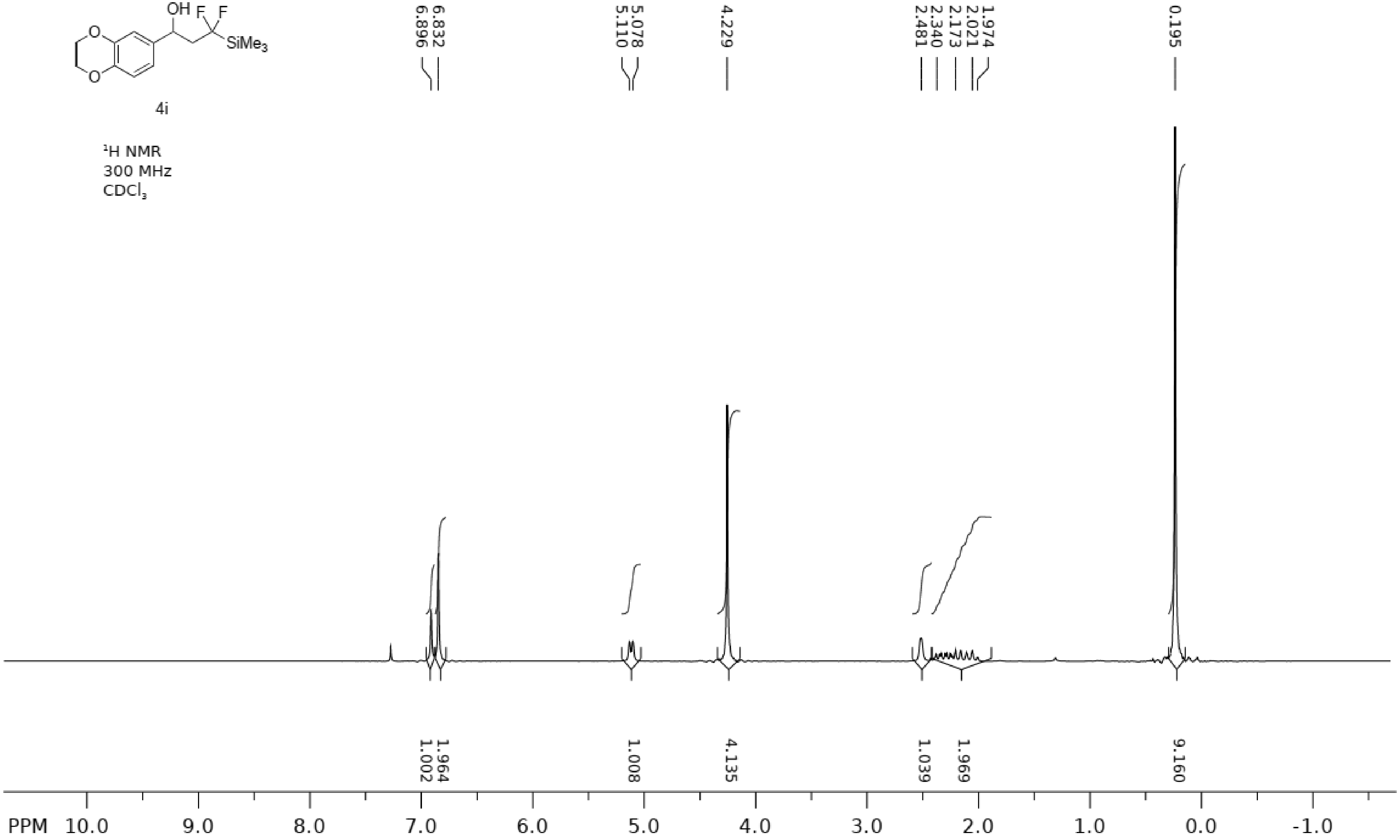
OH F F
O
O
SiMe3
4i
¹H NMR
300 MHz
CDCl3
6.832
6.896
5.078
5.110
4.229
1.974
2.021
2.173
2.340
2.481
0.195
1.964
1.002
1.008
4.135
1.039
1.969
9.160
PPM 10.0 9.0 8.0 7.0 6.0 5.0 4.0 3.0 2.0 1.0 0.0 -1.0

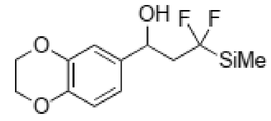

4i

$^{13}C\{^{1}H\}$ NMR
75 MHz
$CDCl_3$

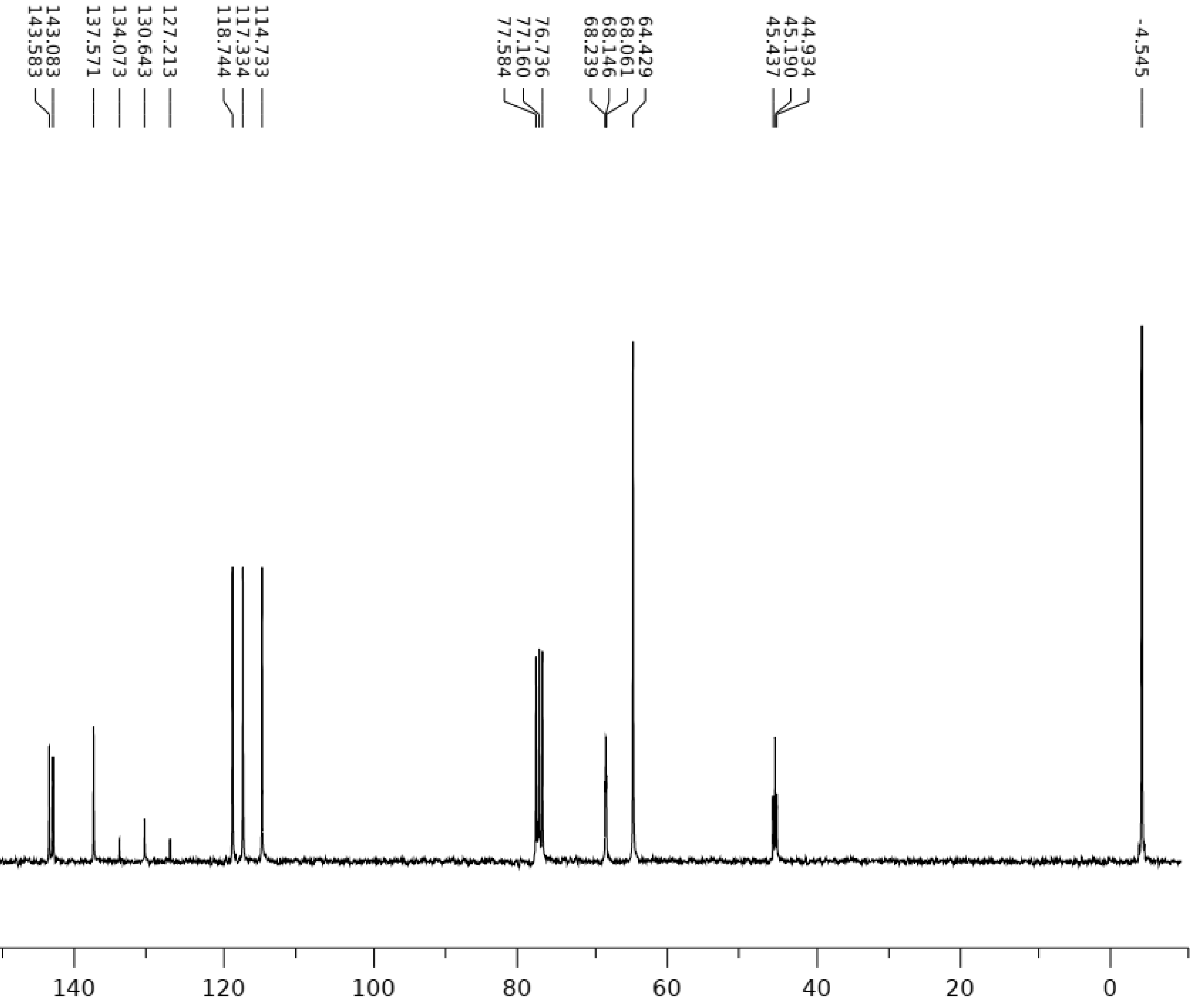

4i

^{19}F NMR
282 MHz
$CDCl_3$

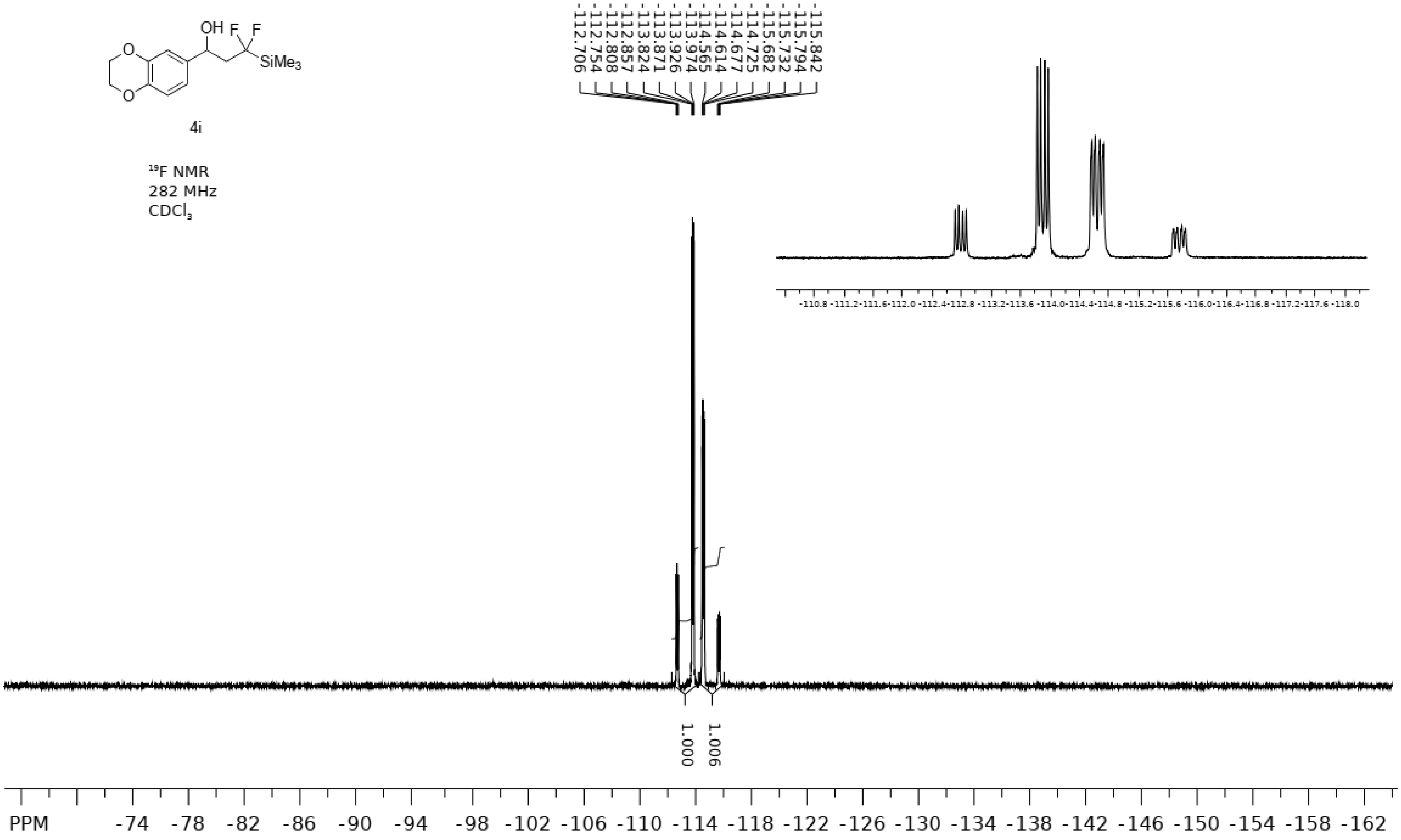

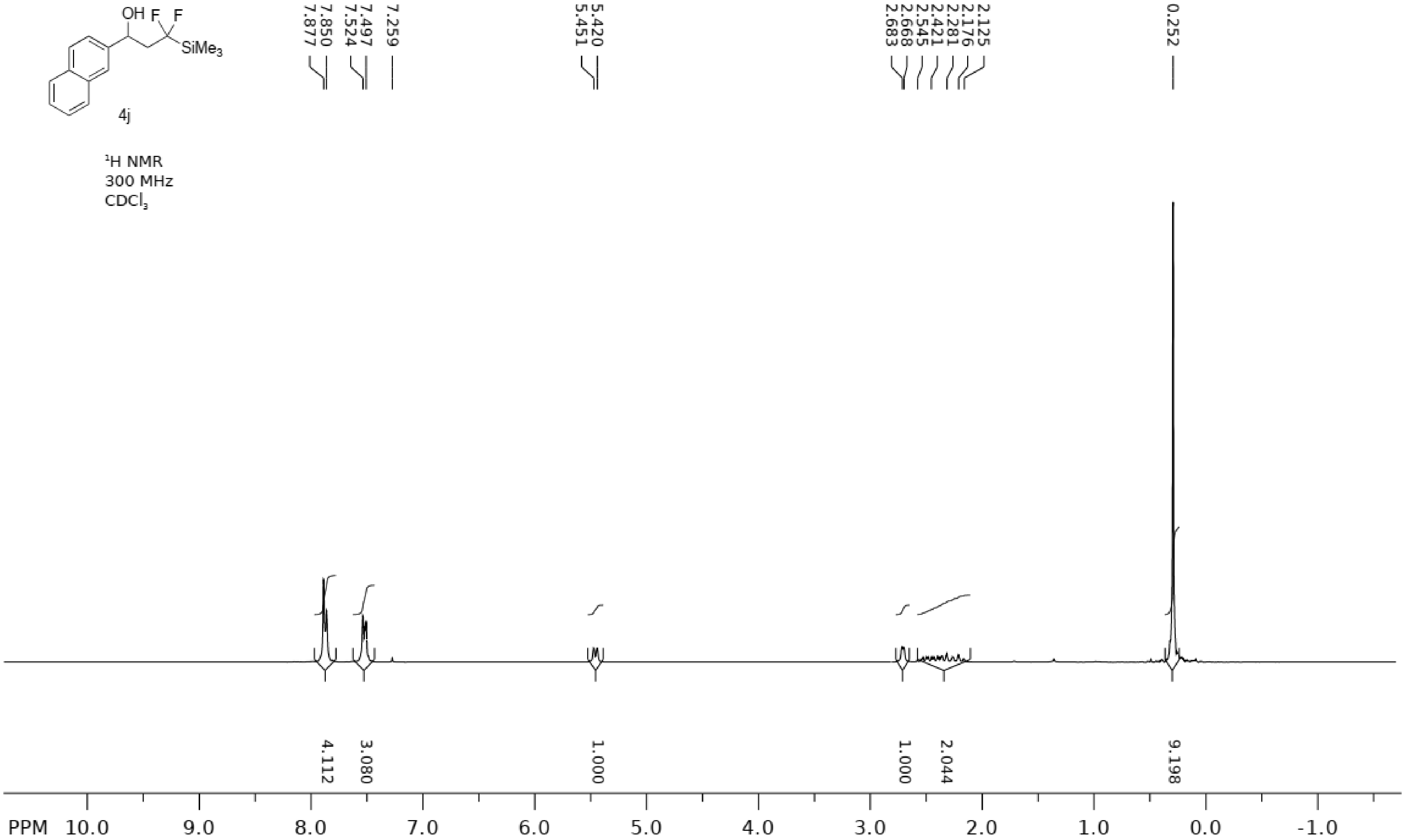
OH
F F
SiMe3
4j
¹H NMR
300 MHz
CDCl3
7.877
7.850
7.524
7.497
7.259
5.451
5.420
2.683
2.668
2.545
2.421
2.281
2.176
2.125
0.252
4.112
3.080
1.000
1.000
2.044
9.198
PPM 10.0 9.0 8.0 7.0 6.0 5.0 4.0 3.0 2.0 1.0 0.0 -1.0

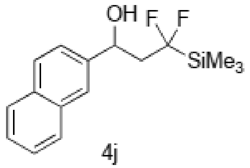

4j

$^{13}C\{^{1}H\}$ NMR
75 MHz
$CDCl_3$

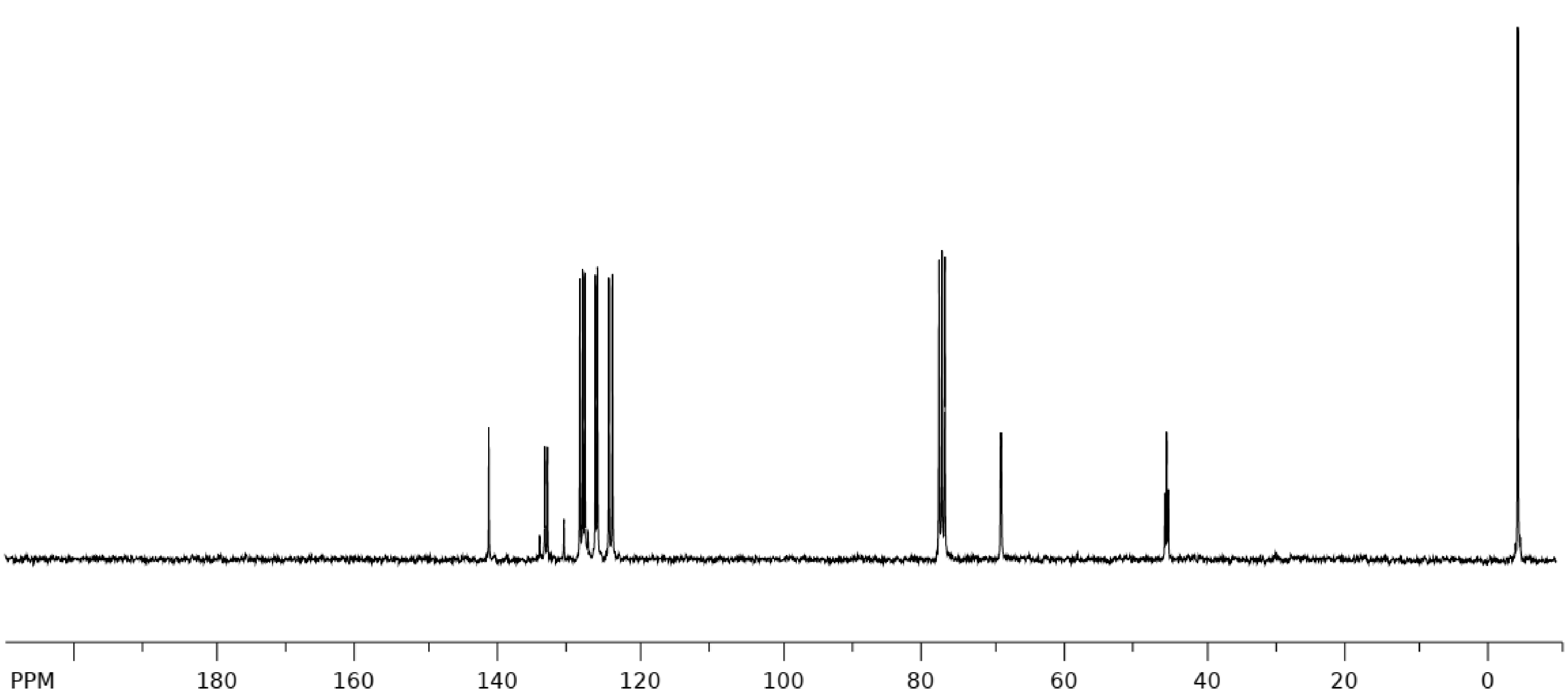

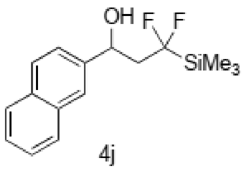

4j

^{19}F NMR
282 MHz
$CDCl_3$

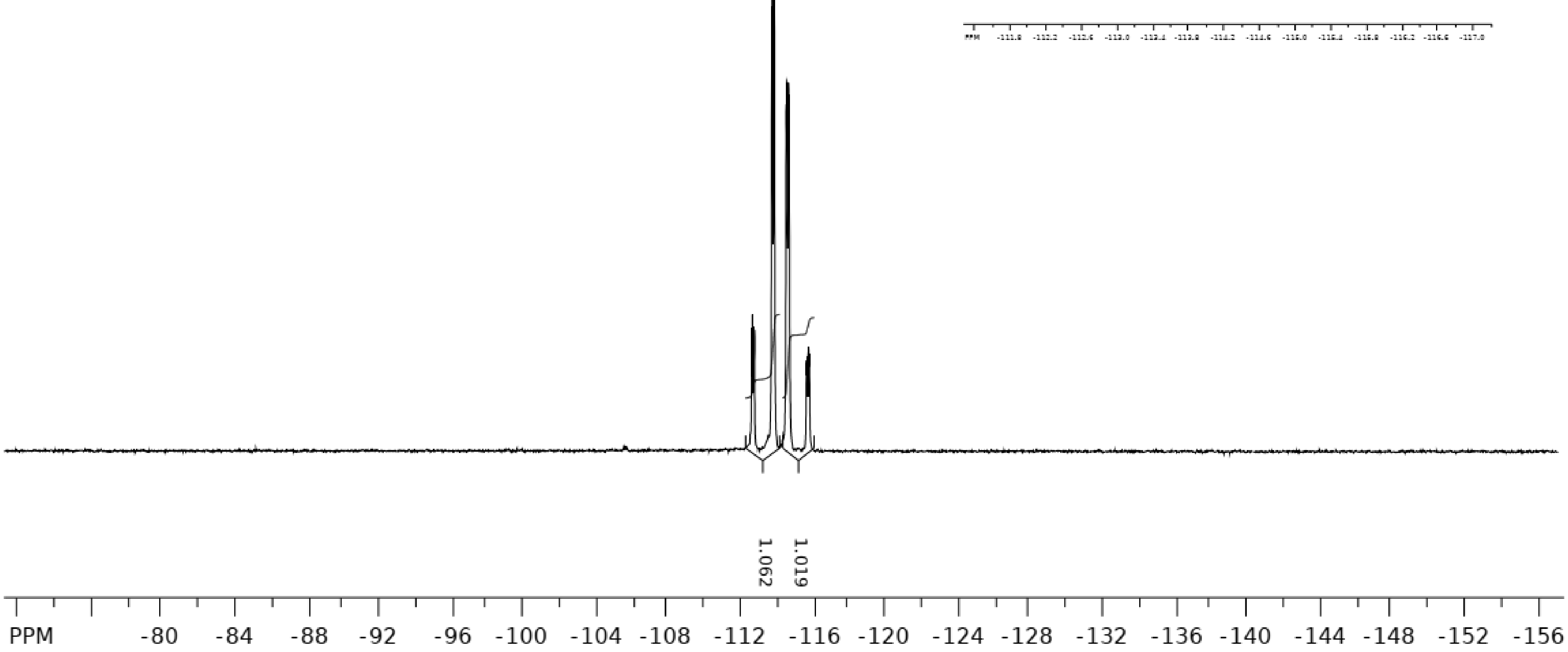

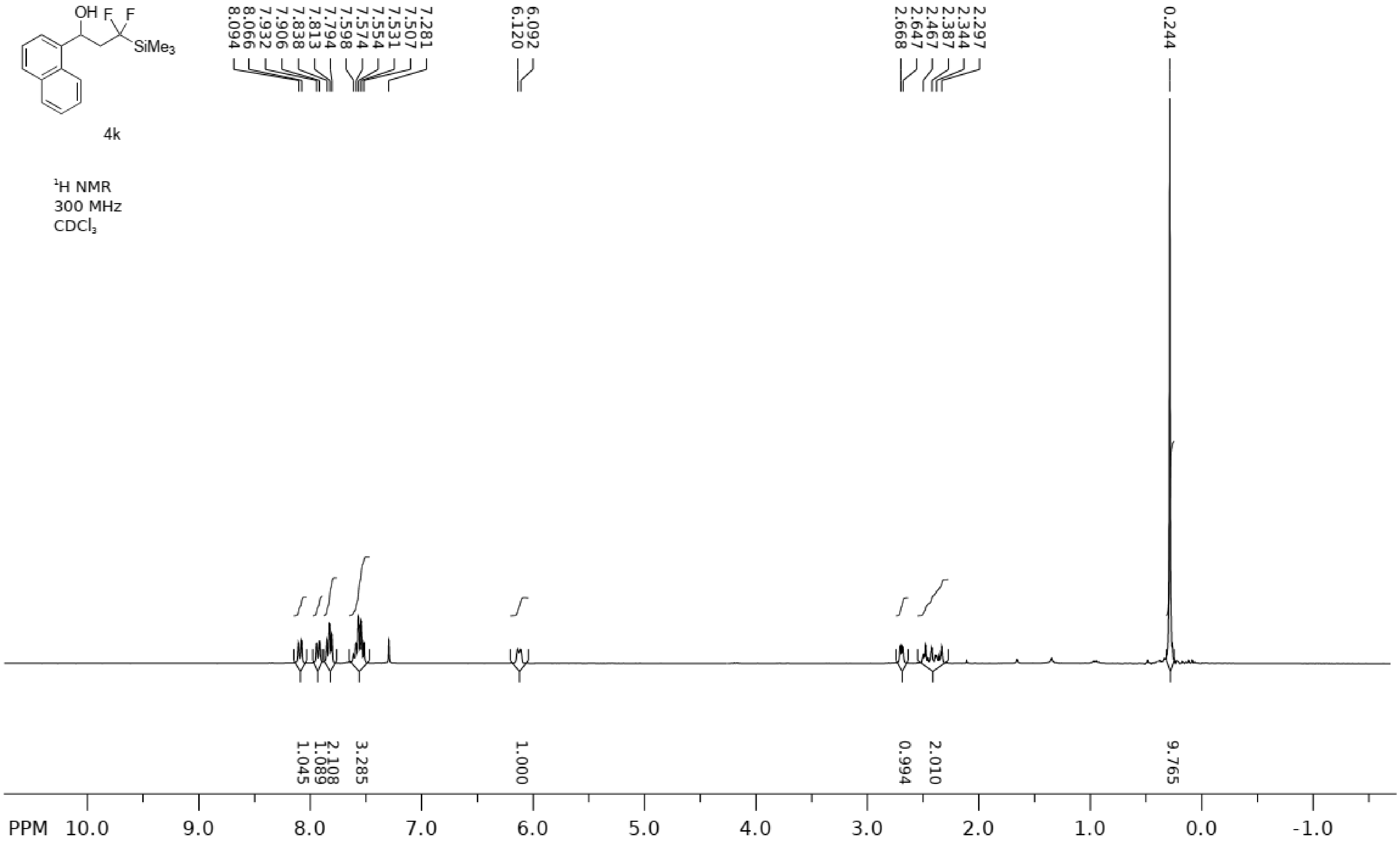
OH
F
F
SiMe3
4k
1H NMR
300 MHz
CDCl3
8.094
8.066
7.932
7.906
7.838
7.813
7.794
7.598
7.574
7.554
7.531
7.507
7.281
6.120
6.092
2.668
2.647
2.467
2.387
2.344
2.297
0.244
1.045
1.089
2.108
3.285
1.000
0.994
2.010
9.765
PPM
10.0
9.0
8.0
7.0
6.0
5.0
4.0
3.0
2.0
1.0
0.0
-1.0

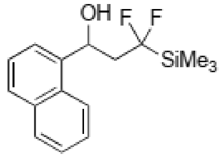

4k

$^{13}C\{^{1}H\}$ NMR
75 MHz
$CDCl_3$

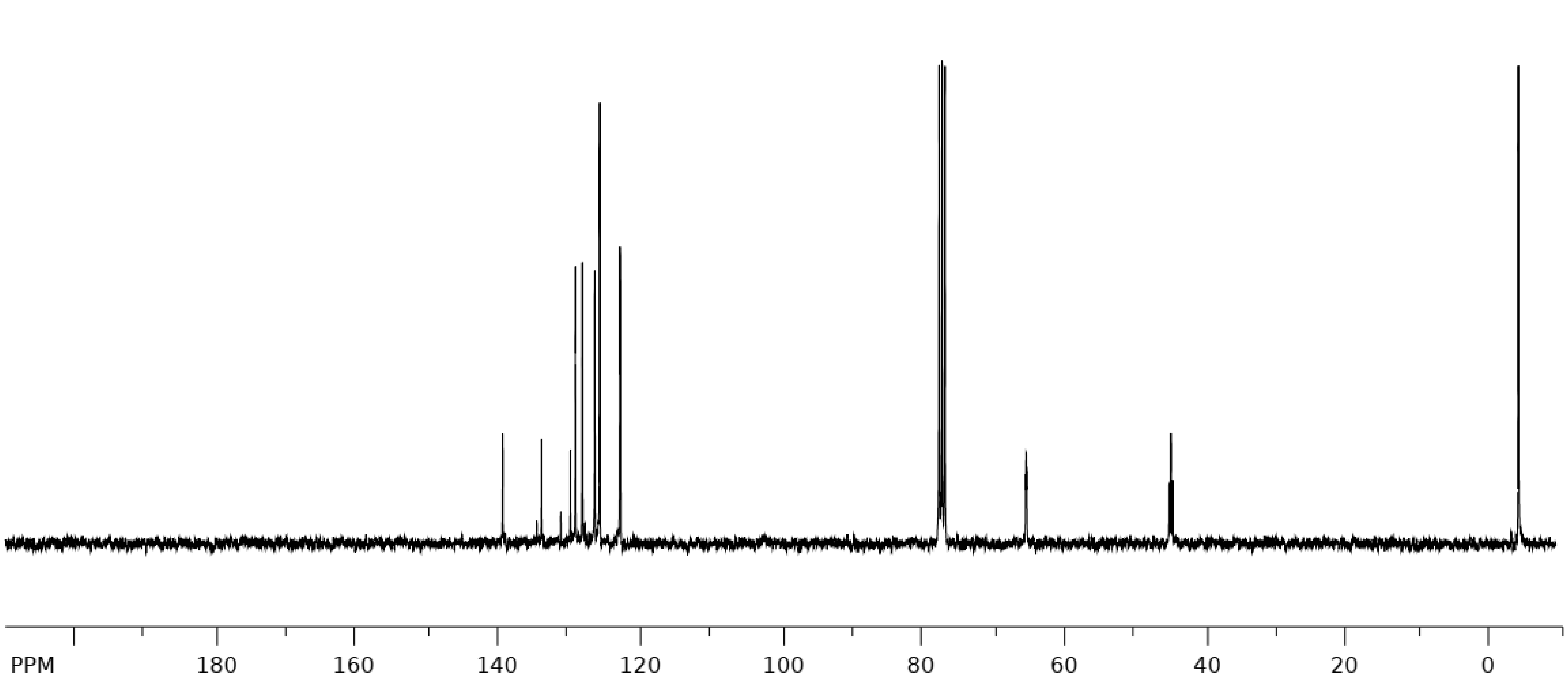

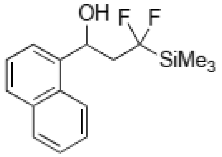

4k

¹⁹F NMR
282 MHz
$CDCl_3$

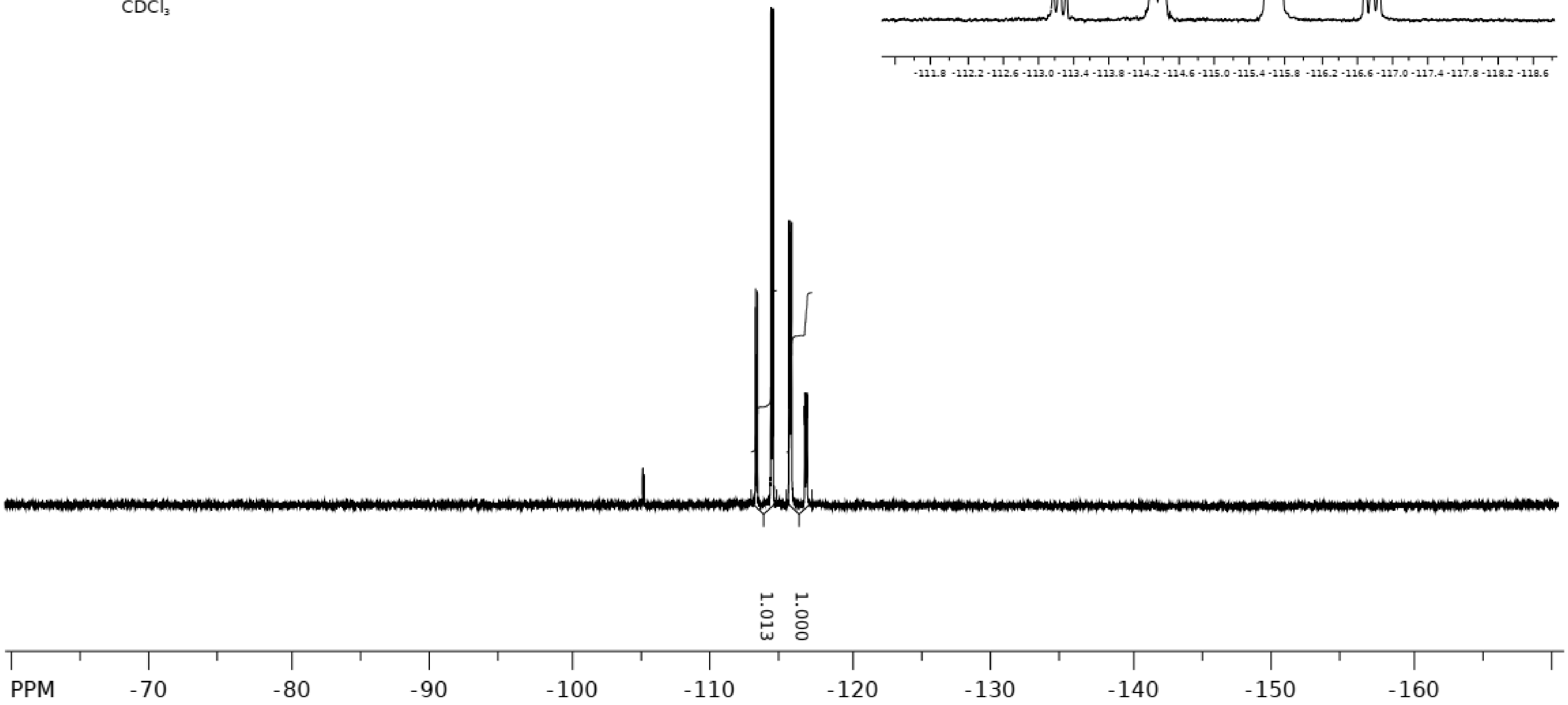

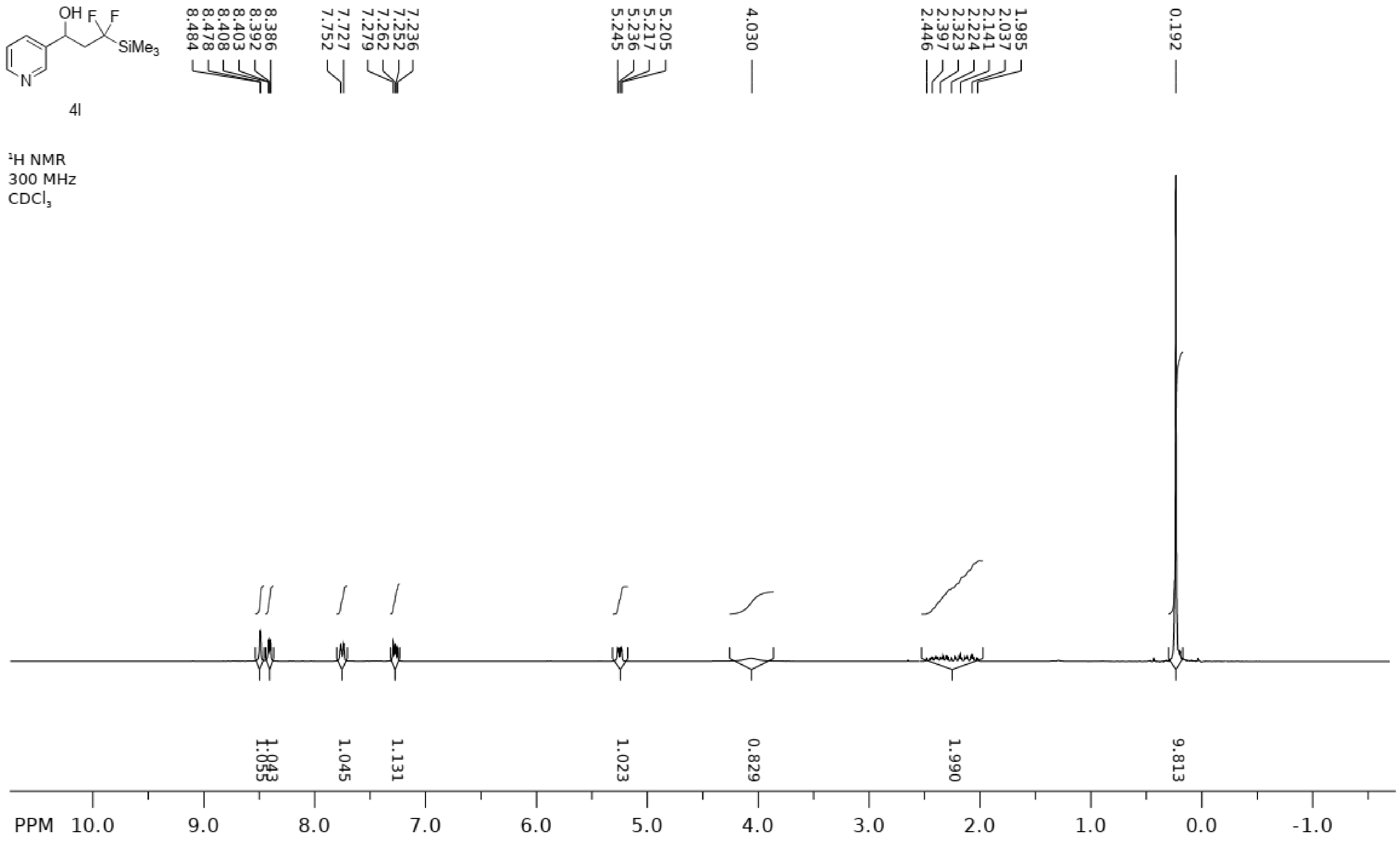

4l

^{1}H NMR
300 MHz
$CDCl_3$

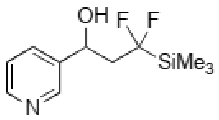

4l

$^{13}C\{^{1}H\}$ NMR
75 MHz
$CDCl_3$

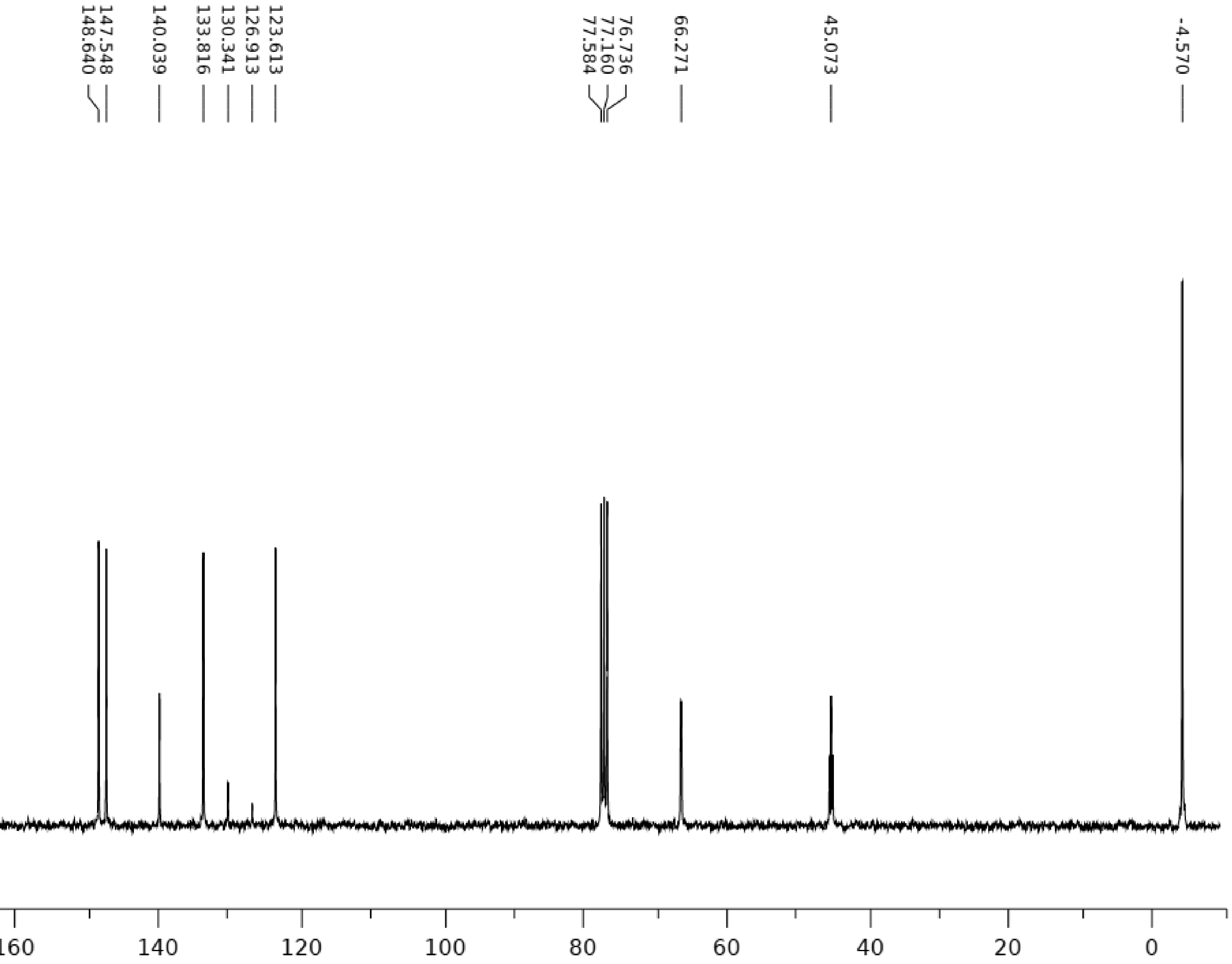

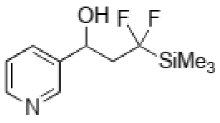

4l

^{19}F NMR
282 MHz
$CDCl_3$

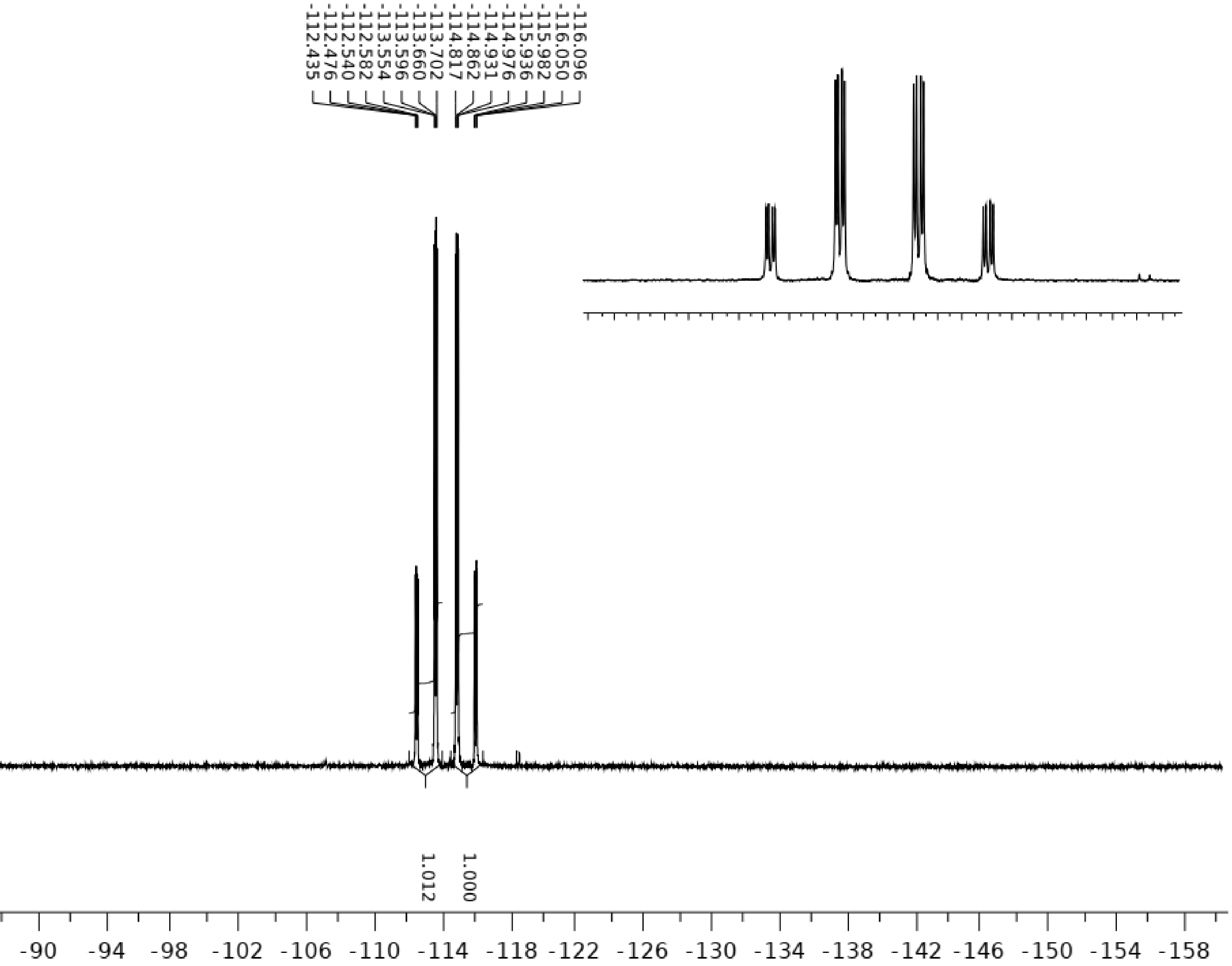

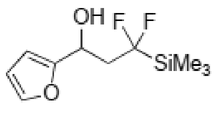

4m

^{1}H NMR
300 MHz
$CDCl_3$

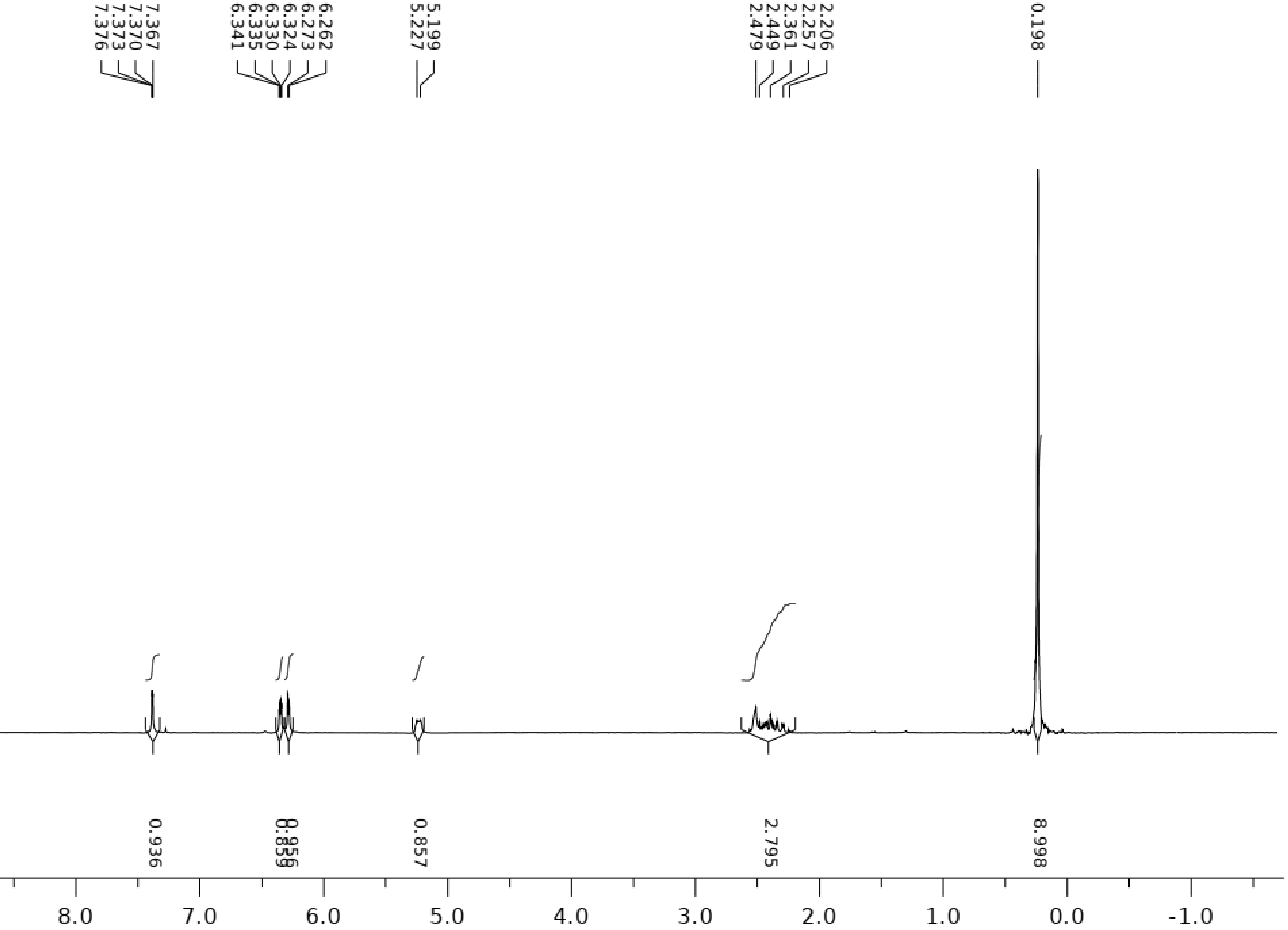

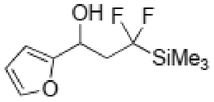

4m

$^{13}C\{^1H\}$ NMR
75 MHz
$CDCl_3$

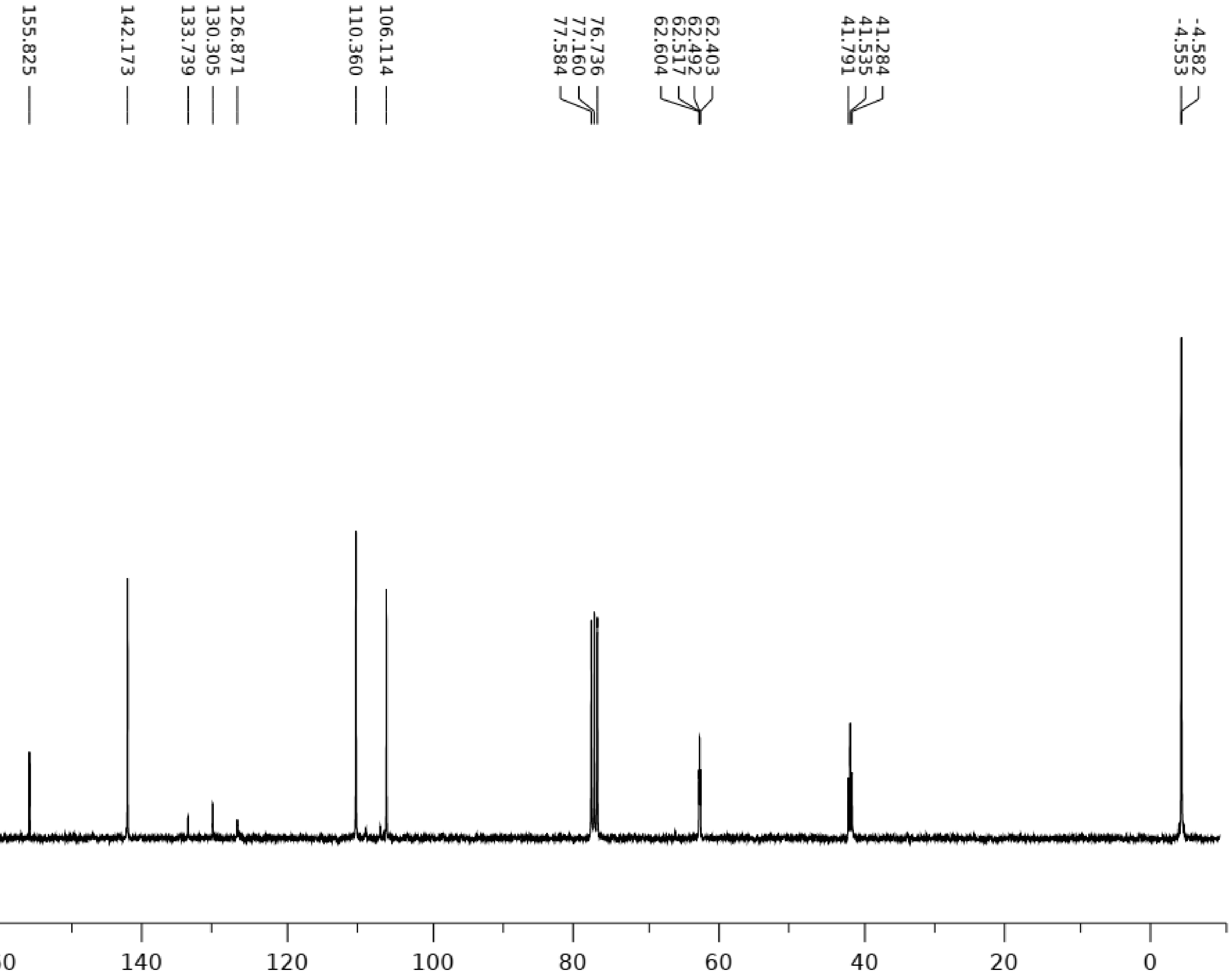

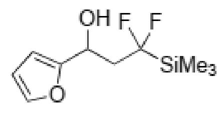

4m

^{19}F NMR
282 MHz
$CDCl_3$

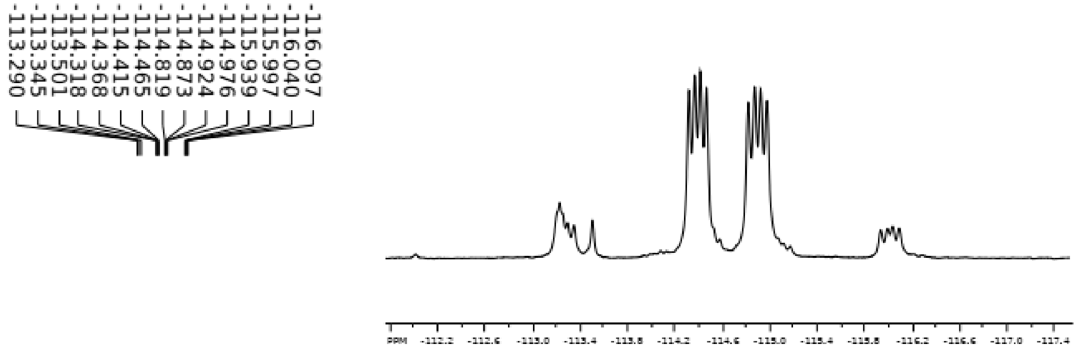

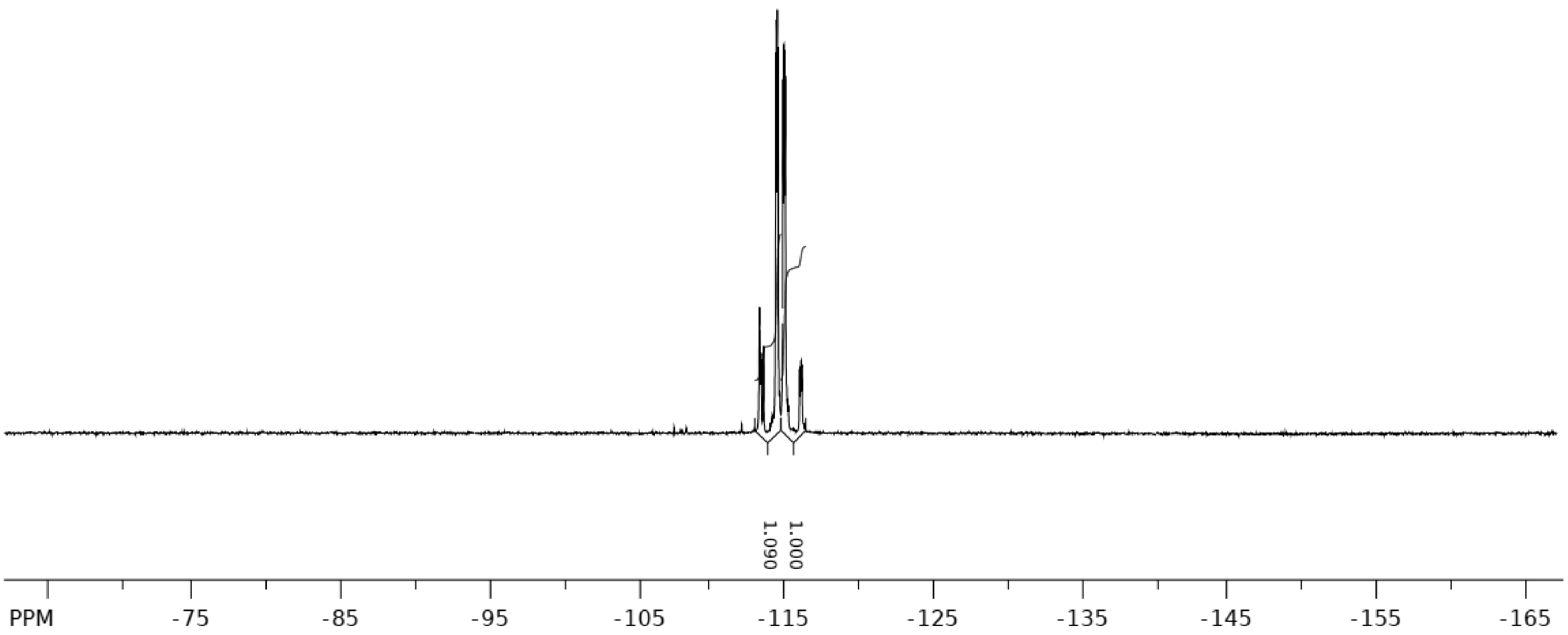

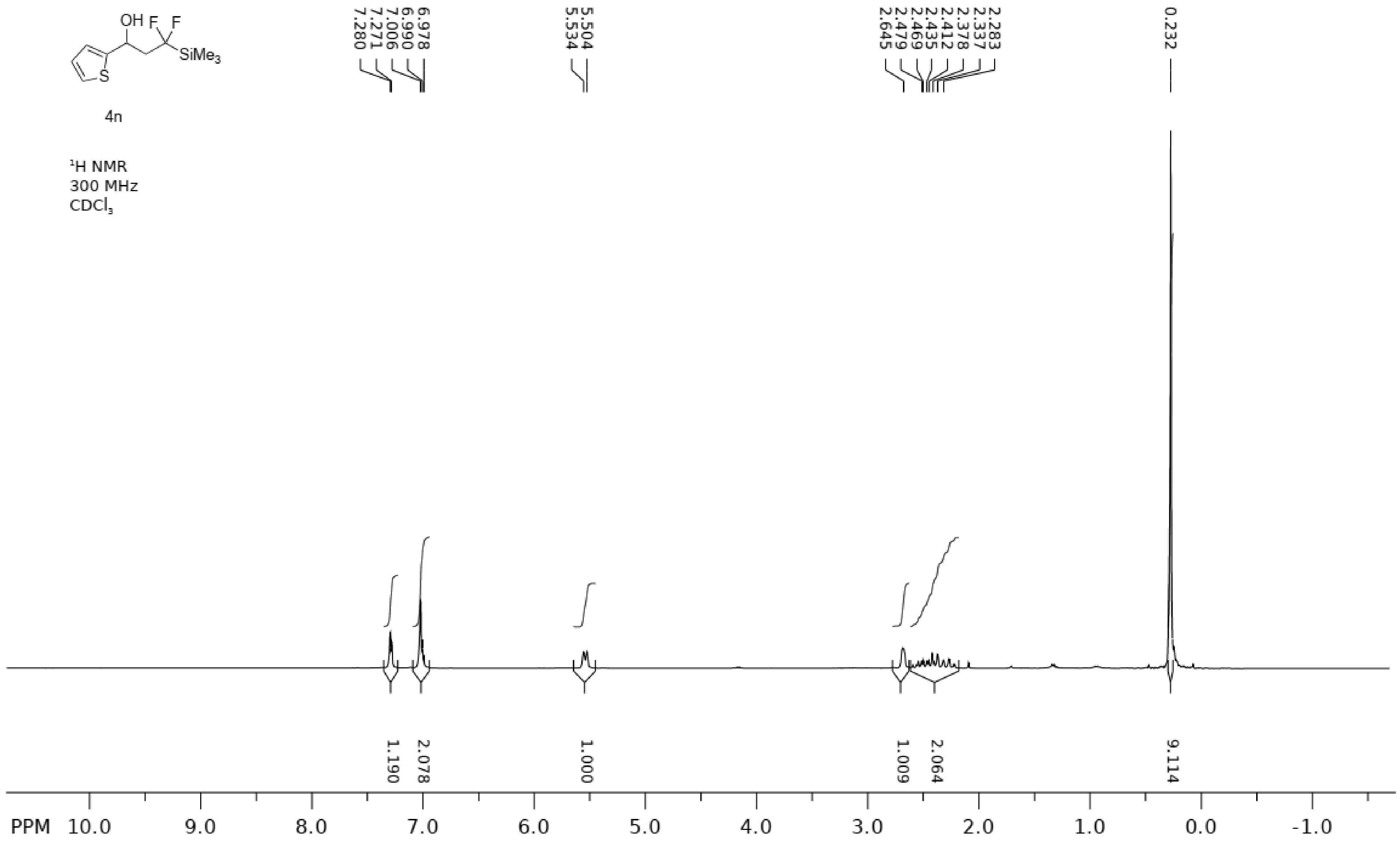
OH F F
SiMe3
S
4n
1H NMR
300 MHz
CDCl3
7.280
7.271
7.006
6.990
6.978
5.534
5.504
2.645
2.479
2.469
2.435
2.412
2.378
2.337
2.283
0.232
1.190
2.078
1.000
1.009
2.064
9.114
PPM 10.0 9.0 8.0 7.0 6.0 5.0 4.0 3.0 2.0 1.0 0.0 -1.0

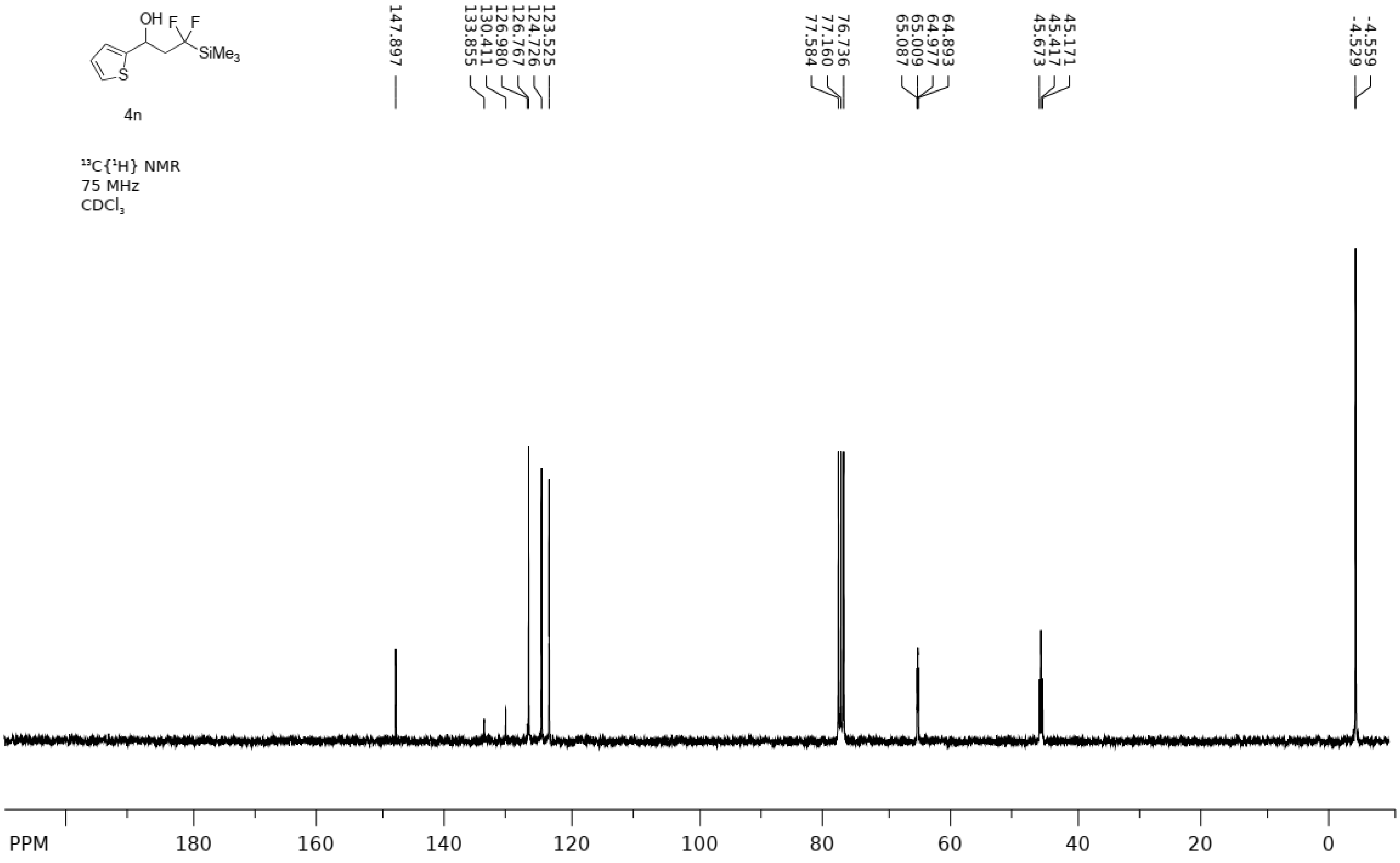
OH
F
F
SiMe3
S
4n
13C{1H} NMR
75 MHz
CDCl3
147.897
133.855
130.411
126.980
126.767
124.726
123.525
77.584
77.160
76.736
65.087
65.009
64.977
64.893
45.673
45.417
45.171
-4.529
-4.559
PPM
180
160
140
120
100
80
60
40
20
0

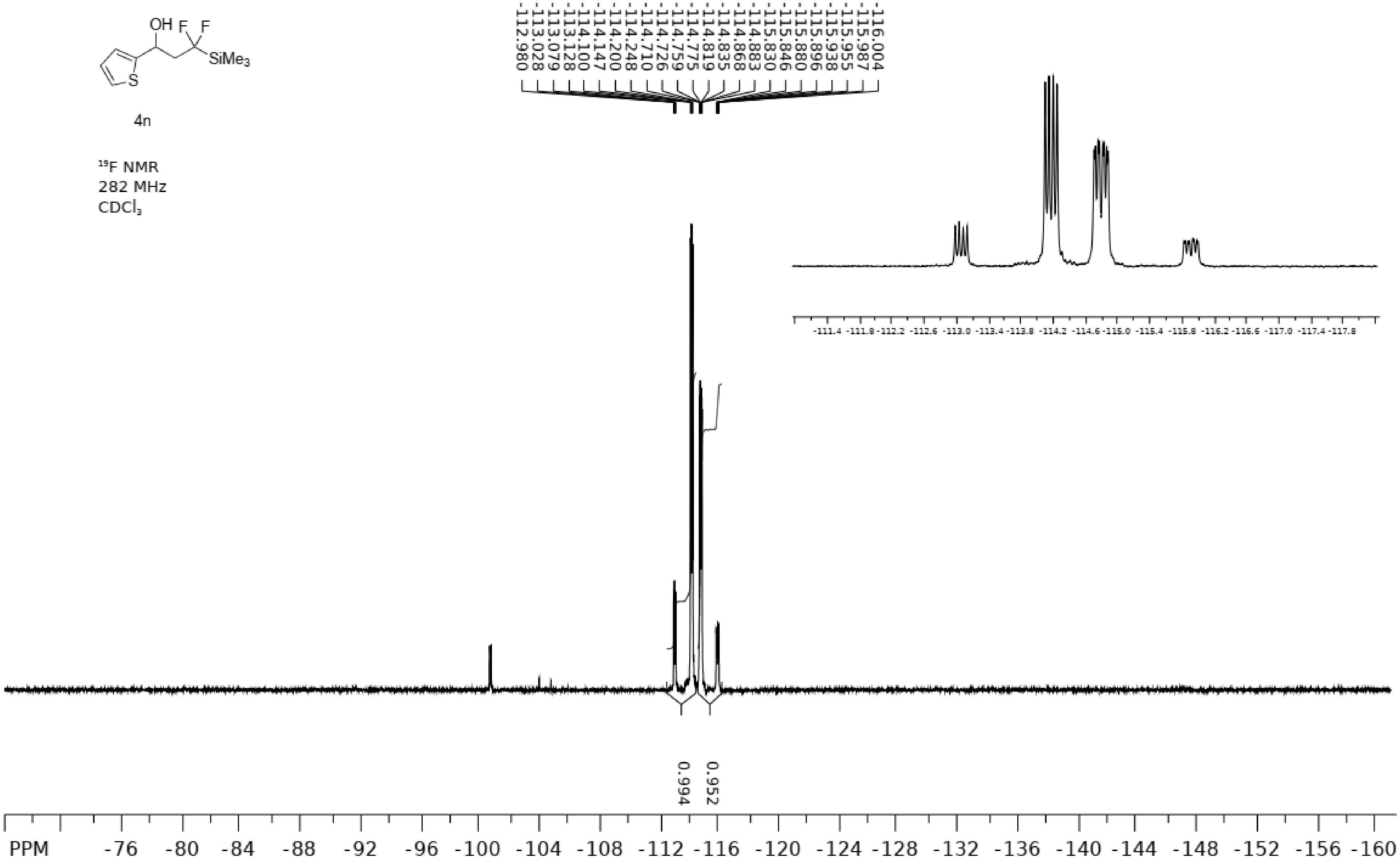
OH F F
SiMe3
S
4n
19F NMR
282 MHz
CDCl3
-112.980
-113.028
-113.079
-113.128
-114.100
-114.147
-114.200
-114.248
-114.710
-114.726
-114.759
-114.775
-114.819
-114.835
-114.868
-114.883
-115.830
-115.846
-115.880
-115.896
-115.938
-115.955
-115.987
-116.004
0.994
0.952
PPM -76 -80 -84 -88 -92 -96 -100 -104 -108 -112 -116 -120 -124 -128 -132 -136 -140 -144 -148 -152 -156 -160

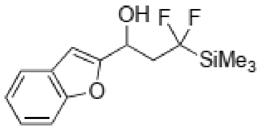

4o

^{1}H NMR
300 MHz
$CDCl_3$

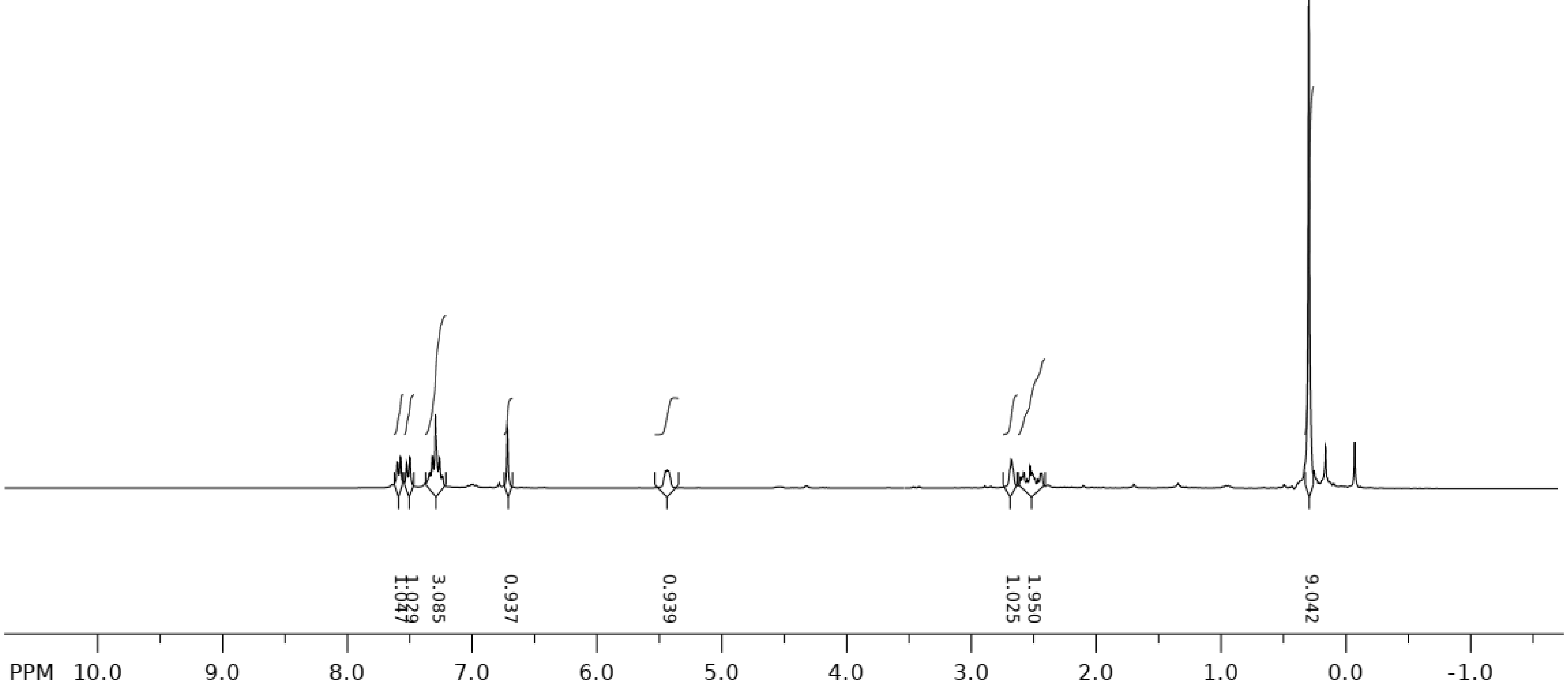

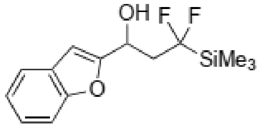

4o

$^{13}C\{^{1}H\}$ NMR
75 MHz
$CDCl_3$

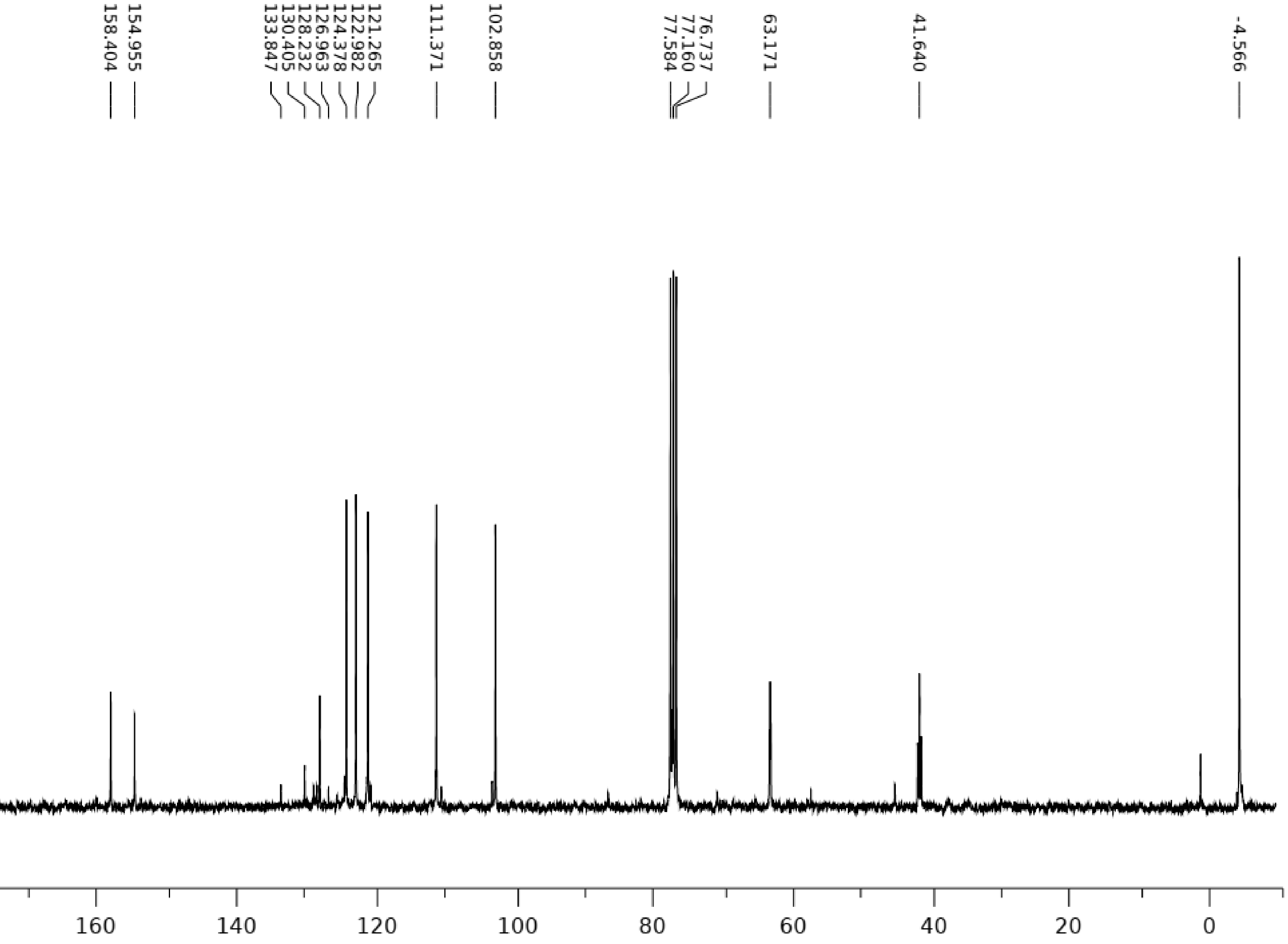

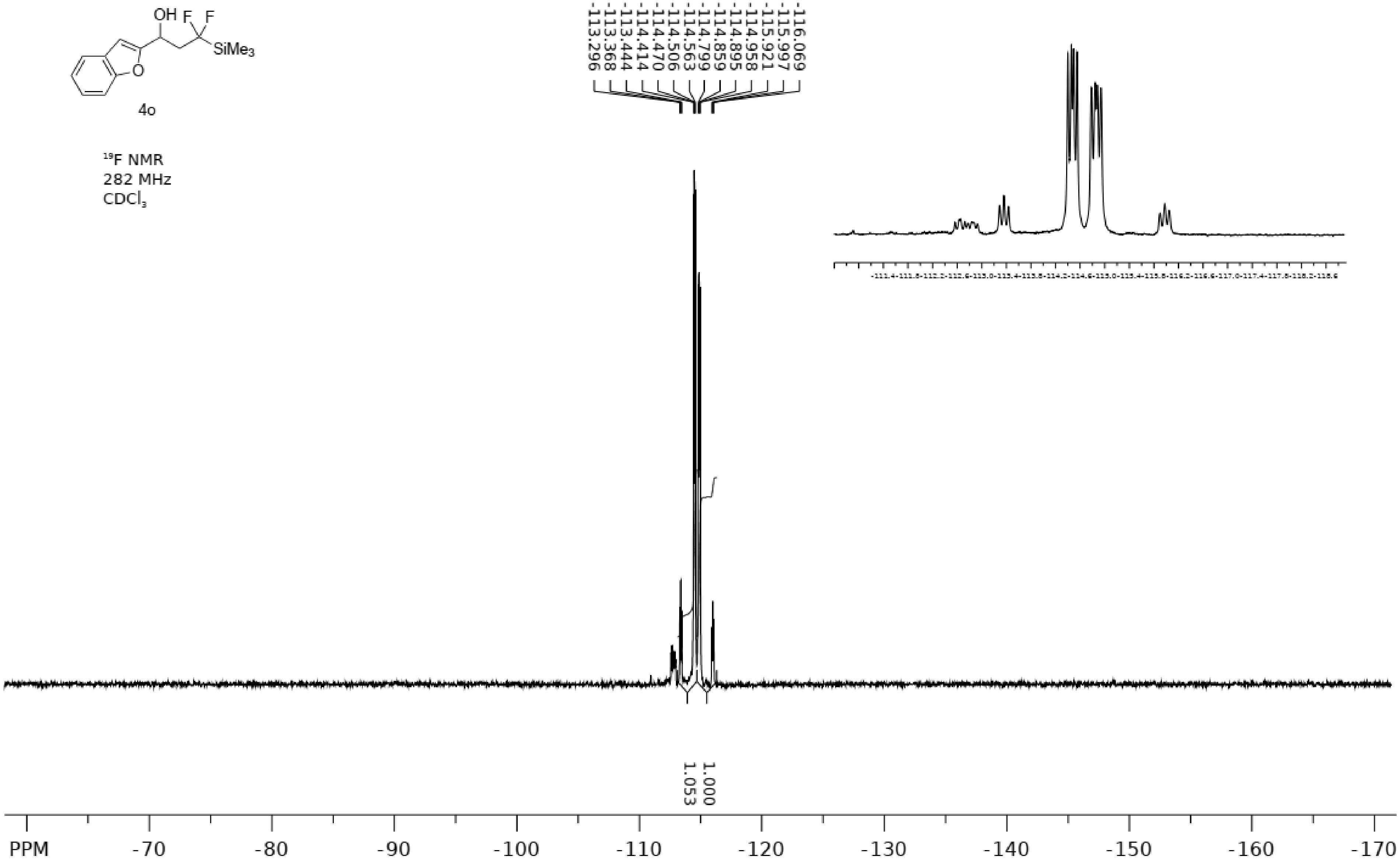

OH
F
F
SiMe3
O
4o
19F NMR
282 MHz
CDCl3
-113.296
-113.368
-113.444
-114.414
-114.470
-114.506
-114.563
-114.799
-114.859
-114.895
-114.958
-115.921
-115.997
-116.069
1.053
1.000
PPM
-70
-80
-90
-100
-110
-120
-130
-140
-150
-160
-170

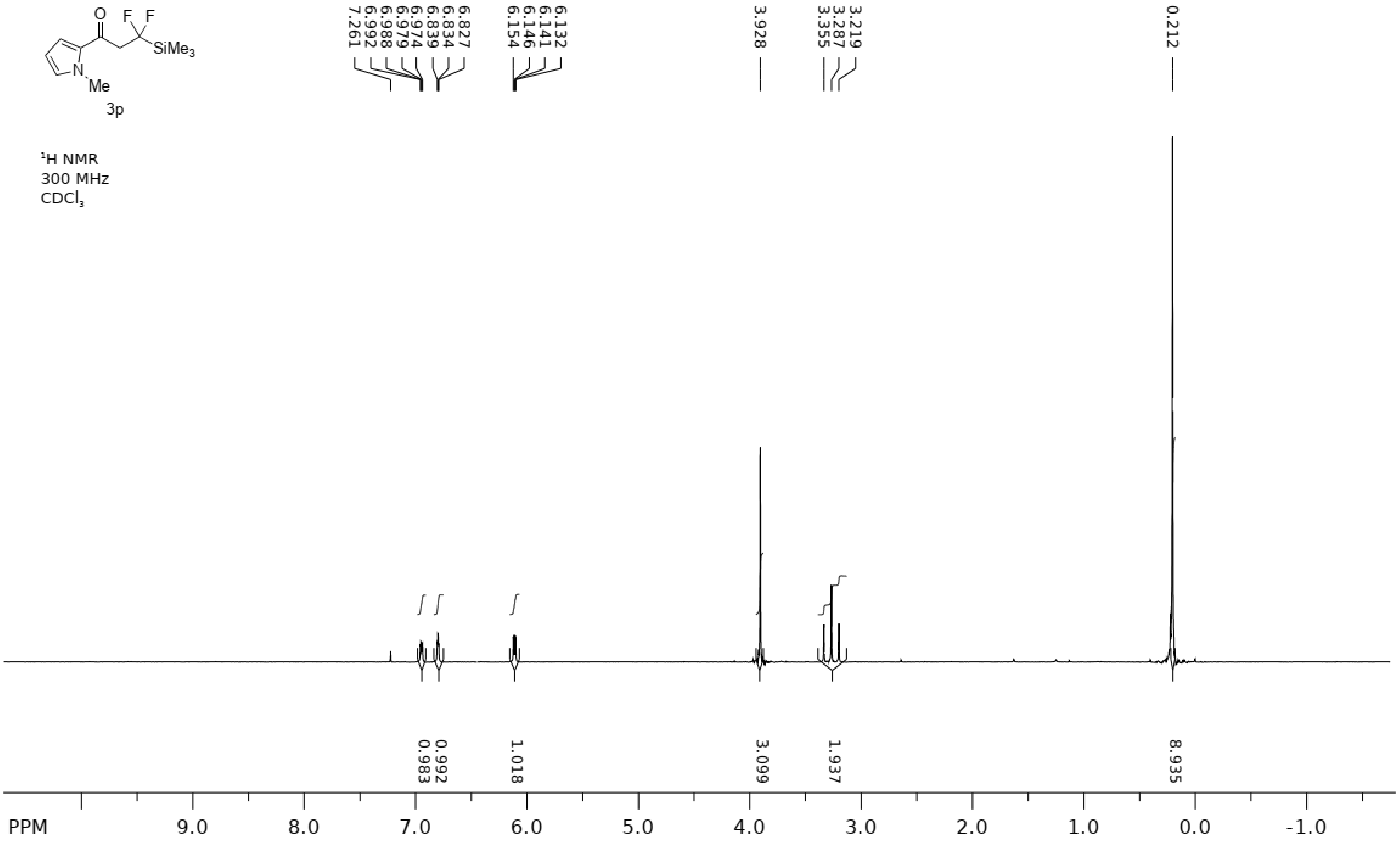
O
F F
SiMe3
N
Me
3p
1H NMR
300 MHz
CDCl3
7.261
6.992
6.988
6.979
6.974
6.839
6.834
6.827
6.154
6.146
6.141
6.132
3.928
3.355
3.287
3.219
0.212
0.983
0.992
1.018
3.099
1.937
8.935
PPM
9.0
8.0
7.0
6.0
5.0
4.0
3.0
2.0
1.0
0.0
-1.0

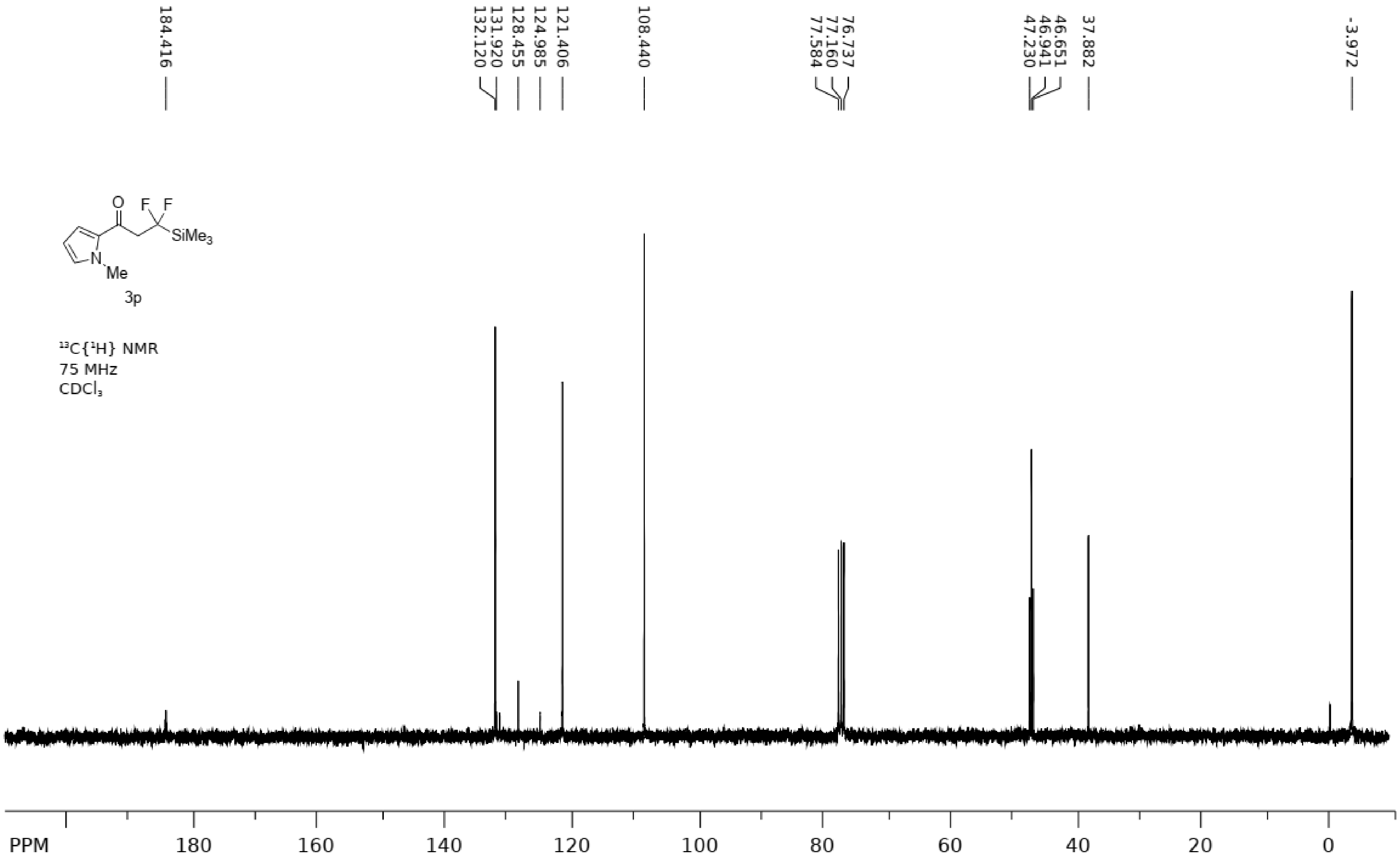
184.416
132.120
131.920
128.455
124.985
121.406
108.440
77.584
77.160
76.737
47.230
46.941
46.651
37.882
-3.972
3p
13C{1H} NMR
75 MHz
CDCl3
PPM
180
160
140
120
100
80
60
40
20
0

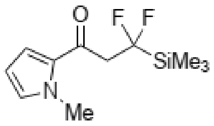

3p

^{19}F NMR
282 MHz
$CDCl_3$

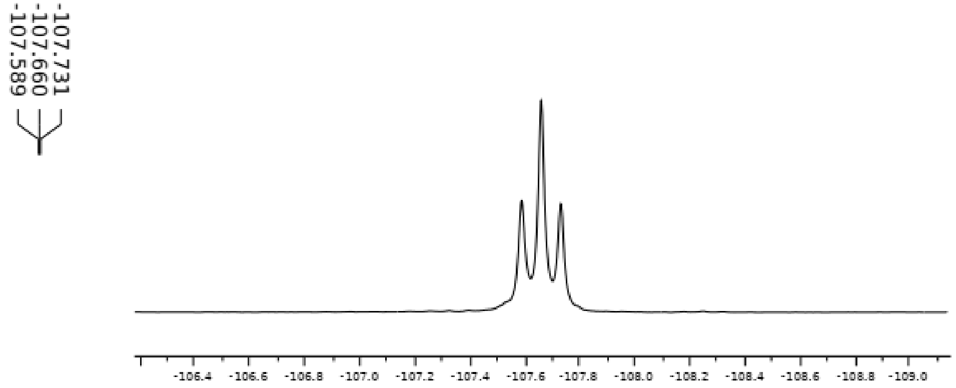

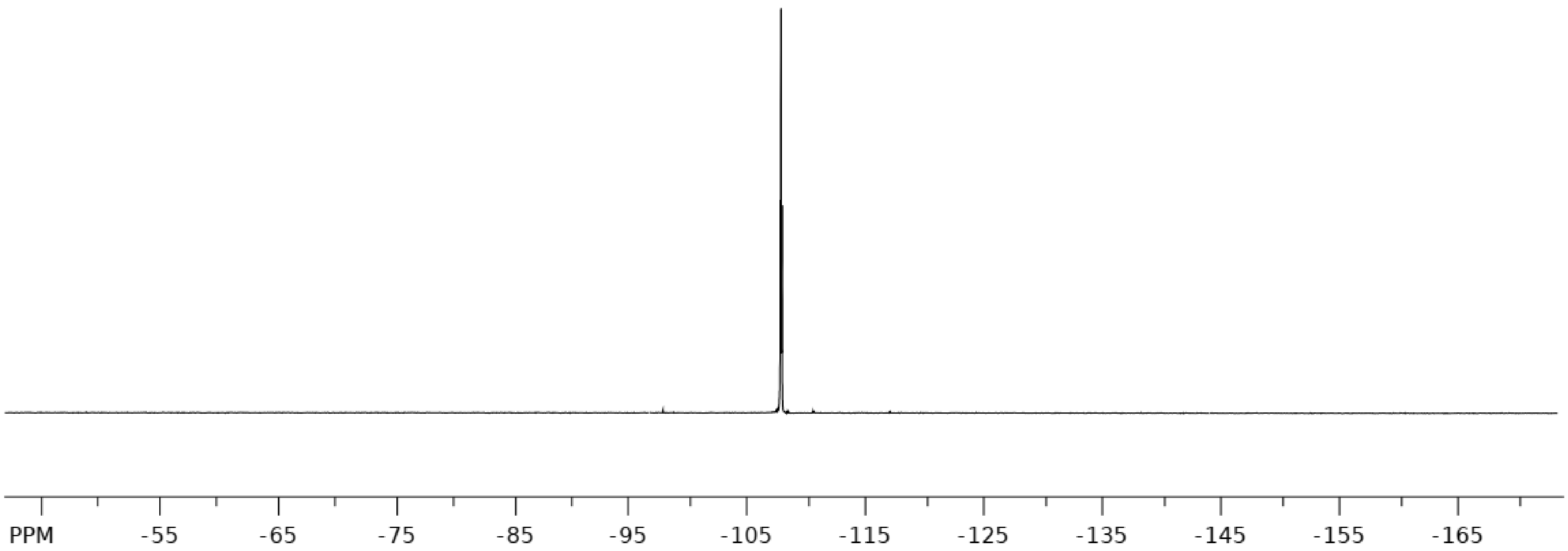

Supplement: File 1 — Full experimental details, compound characterization, and copies of NMR spectra. [file Beilstein_J_Org_Chem-16-1550-s001.pdf]
